# Supplementary material for: Adversarial testing of global neuronal workspace and integrated information theories of consciousness
Source: Nature. 2025 Apr 30;642(8066):133–42. doi: 10.1038/s41586-025-08888-1 (PMC12137136; doi:10.1038/s41586-025-08888-1)
Supplement: Supplementary file 1 — Supplementary Methods and Results, Supplementary Discussion and Supplementary Notes. [file 41586_2025_8888_MOESM1_ESM.pdf]

nature portfolio

https://doi.org/10.1038/s41586-025-08888-1

Supplementary information

Adversarial testing of global neuronal workspace and integrated information theories of consciousness

In the format provided by the
authors and unedited

Supplementary Information

Table of Contents

Supplementary Methods and Results
1. Behavioral analysis 4
Pre-registered analyses 4
1.1 Overall Behavioral and Eye Tracking Performance in the task across modalities 4
1.2 d' analysis 5
1.3 Hit rate analysis 8
1.4 False Alarm analysis 8
1.5 Reaction times analysis 11
2. Eye movements analyses 13
Pre-registered analyses 13
2.1 First 0.5 s: Fixation distance from center 13
2.2 First 0.5 s: Number of blinks 14
2.3 First 0.5 s: Saccade amplitude 16
2.4 First 0.5 s: Pupil size 17
2.5 Long trials analysis: Fixation distance from center 19
2.6 Long trials analysis: Number of blinks 19
2.7 Long trials analysis: Saccade amplitude 22
2.8 Long trials analysis: Pupil size 23
3. Control experiment: Surprise memory test 25
3.1 Methods 25
3.1.1 Participants 25
3.1.2 Apparatus 25
3.1.3 Stimuli and procedure 25
3.1.4 Analysis 26
3.2 Results 27

3.2.1 Exposure phase 27
3.2.2 Memory phase 27
3.3 Conclusions 29
4. Replicability of findings: Optimization vs. replication results 30
4.1 Behavioral analysis 30
4.2 Eye movements analysis 30
4.3 Decoding analysis 32
4.3.1 MEG 32
4.3.2 fMRI 34
4.4 Levels of activation analysis 35
4.4.1 MEG 35
4.5 Synchrony analysis 37
4.5.1 MEG 37
4.5.2 fMRI 39
4.6 Putative NCC analysis 40
4.6.1 fMRI 40
5. Prediction #1: Decoding of conscious content 41
5.1 Pre-registered analyses 41
5.1.1 Category Decoding 41
5.1.2 Orientation decoding 47
5.1.3 Category and orientation decoding with and without PFC (including inferior frontal sulcus) 51
6. Prediction #2: Maintenance of conscious content over time 52
6.1 Pre-registered analyses: Tracking of duration 52
6.1.1 iEEG: Duration tracking in the different signals 52
6.1.2 iEEG: Onset responsiveness and category selectivity 56
6.1.3 MEG: Duration tracking in the different signals 58
6.2 Exploratory analyses: Duration predictions 65
6.2.1 iEEG: Exploratory decoding analysis, with unrestricted temporal profiles and time windows 65
6.2.2 iEEG: Onset/offset analysis 68
6.2.3 iEEG: Sustained duration tracking 69
6.2.4 MEG: Gamma power in unified ROIs 70

6.2.5 MEG: Alpha power in unified ROIs 71
6.2.6 MEG: ERFs in unified ROIs 73
6.2.7 MEG: Alpha power using delayed time-windows on the unified ROIs 74
6.2.8 MEG: Onset/offset analysis 75
6.2.9 MEG: Task relevant (non-target) analysis 77
6.3 iEEG Pre-registered analyses: Representational Similarity Analysis (RSA) 79
6.3.1 iEEG: RSA analysis 79
6.4 iEEG Exploratory analyses: RSA 81
6.4.1 Feature selection 81
6.4.2 GNWT extended windows 85
6.4.3 GNWT model including only onset 86
6.4.4 iEEG cross-task decoding at stimulus offset 88
6.5 MEG Pre-registered analyses: Representational Similarity Analysis (RSA) 88
6.5.1 MEG RSA analysis 88
7. Prediction #3: Interareal functional connectivity 92
7.1 Pre-registered analyses 92
7.1.1 fMRI Generalized Psycho-Physiological Interaction (gPPI) Table 92
7.1.2 Task Relevant condition 93
7.1.3 Task Relevant and Task Irrelevant combined 97
8. Putative Neural Correlates of (Visual) Consciousness (pNCC) 100
8.1. Pre-registered analyses 100
8.1.1 Univariate pNCC analysis 104
8.1.2 Univariate Participant-level pNCC analysis 109
8.1.3 Multivariate pNCC analysis 111
8.1.4 Summary pNCC analysis 113
9. Participants 114
9.1 iEEG demographics 114
10. Anatomical Regions-of-interest (ROIs) 117
10.1 Process for the definition of the ROIs 117

Supplementary Discussion **118**
11. Cogitate Consortium 118
12. Integrated Information Theory: Melanie Boly, Christof Koch, Giulio Tononi 119

12.1 Further observations 121
13. Global Neuronal Workspace Theory: Stanislas Dehaene 122

Supplementary Notes **124**
14. Deviations from the preregistration document 124
15. Author Contributions Grid 126
References 127

Supplementary Methods and Results

1. Behavioral analysis

Analyses were done with R 4.3.1 (ordinal 2023.12.4, tidyR 1.3.0, dplyr 1.1.4, lmerTest 3.1.3, bayestestR 0.14.0, emmeans 1.10.4) and Python (3.9, pandas 1.5.2, numpy 1.21.2, matplotlib 3.6.2, seaborn 0.12.1, scipy 1.7.1)

Pre-registered analyses

1.1 Overall Behavioral and Eye Tracking Performance in the task across modalities

Participants' performance in the task was excellent, with high hit rates (M=96.84%, SD=4.19%), low false alarm rates (M=1.45%, SD=4.30%), and high fixation stability (mean accuracy <2°=89.62%, SD=10.61%; Supplementary Figure 1a-d). Participants' performance across laboratories within each data modality was similar (all p=1.000 after multiple comparison correction, BF_{01}=8.33). Epilepsy patients showed slightly lower behavioral performance compared to neurotypical participants, yet, behavior was still comparatively high (hit rate 93.90%, SD=12.29; false alarm rate M=4.25%, SD=20.17). We confirmed that participants were conscious of the stimuli both in the task-relevant and irrelevant trials in a separate experiment which included a surprise memory test (see Supplementary Section 3). Below, we report the results of each preregistered analysis.

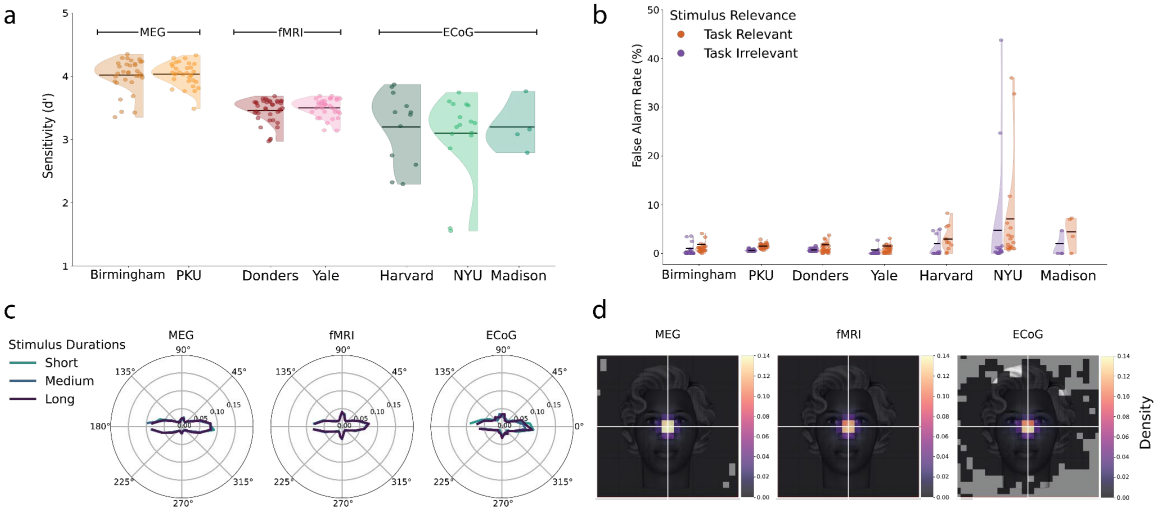

Supplementary Figure 1. a. Distribution of behavioral sensitivity scores (d') separate per data modality and acquisition site. Crossing lines depict average d' per site/modality. Dots depict individual participants d's. Colors depict data modality: MEG N=65 (orange), fMRI N=73 (red), and iEEG N=32 (green), while the hue depicts each site within a modality. **b**. Distributions of false alarm (FA) rates per site and data modality, separated by task condition: Orange-red depicts task relevant stimuli. Purple depicts task irrelevant stimuli. Dots are individual participants' FA rates. Other conventions as in **a**. **c**. Average saccadic direction maps per data modality. The three stimulus durations are shown separately. **d**. Average fixation heatmaps computed over a 0.5 s window after stimulus onset. Heatmaps are displayed per data modality, zoomed into the stimulus area (Face stimuli were created using FaceGen Modeler 3.1.).

1.2 d' analysis

A linear mixed model was used to test if d' is modulated by stimulus category (Faces, Objects, Letters, False Fonts), stimulus duration (0.5, 1, 1.5 s) or modality (iEEG, fMRI, MEG). These factors were defined as fixed effects, and participant was defined as a random effect[1]. The dependent variable was an adjusted d' score (using a log-linear correction). A main effect of modality was found ($F(2,167.04)=102.39$, $p<0.001$, Bayes Factor (BF_{10})=7.80×10^{26}), with MEG participants showing the highest adjusted d' (M=4.02, SD=0.43), followed by the fMRI sample (M=3.48, SD=0.34) which in turn was higher than that of iEEG patients (M=3.15, SD=0.72). The difference in d' between the MEG and fMRI participants likely stems from the different numbers of trials which affects the d' correction, while the lowest d' in iEEG patients was expected given the clinical setting (Supplementary Figure 2; all post-hoc contrasts $p<0.001$). A main effect of category was also found ($F(3, 238.33)=32.17$, $p<0.001$, $BF_{10}=4.34\times10^{14}$), with faces showing a slightly lower d' (M=3.49, SD=0.57) than all other categories (Objects: M=3.68, SD=0.53; Letters: M=3.68, SD=0.58; False Fonts: M=3.64, SD=0.60, all p values <0.001). In addition, d' was lower for false fonts compared to objects (p=0.017). No significant differences were found between the other categories: false fonts vs. letters: p=0.633, letters vs. objects, p=1.000). The slightly lower

d' found for faces could potentially reflect the fact that target faces were harder to individualize and remember compared to the stimuli within the other categories. Notably though, this effect seemed to differ by modality, as revealed by an interaction between modality and category ($F(6, 232.01)=7.47$, $p<0.001$, $BF_{10}=2.94x10^4$). Follow up analyses showed that the general difference between faces and all other categories stemmed from the MEG sample (all three p values <0.001), while for the iEEG sample it was observed for faces vs. objects ($p<0.001$) and faces vs. letters ($p=0.012$), but not false fonts ($p=0.334$), and an additional difference was found in the iEEG sample between false fonts and objects ($p<0.001$). No category differences were found for the fMRI population (p values range between 0.124 and 1.000). Finally, a main effect was also found for stimulus duration ($F(2, 767.32)=11.74$, $p<0.001$, $BF_{10}=776.74$), with the longest duration stimuli evoking a slightly higher d' ($M=3.67$, $SD=0.55$) than the shortest ($M=3.58$, $SD=0.59$, $p<0.001$) but not the intermediate duration stimuli ($M=3.63$, $SD=0.58$, $p=0.053$). The shortest and intermediate duration stimuli also differed from one another ($p=0.019$). No additional interactions were found (p values range between 0.898 ($BF_{01}=16.67$) and 1.000 ($BF_{01}=50$)).

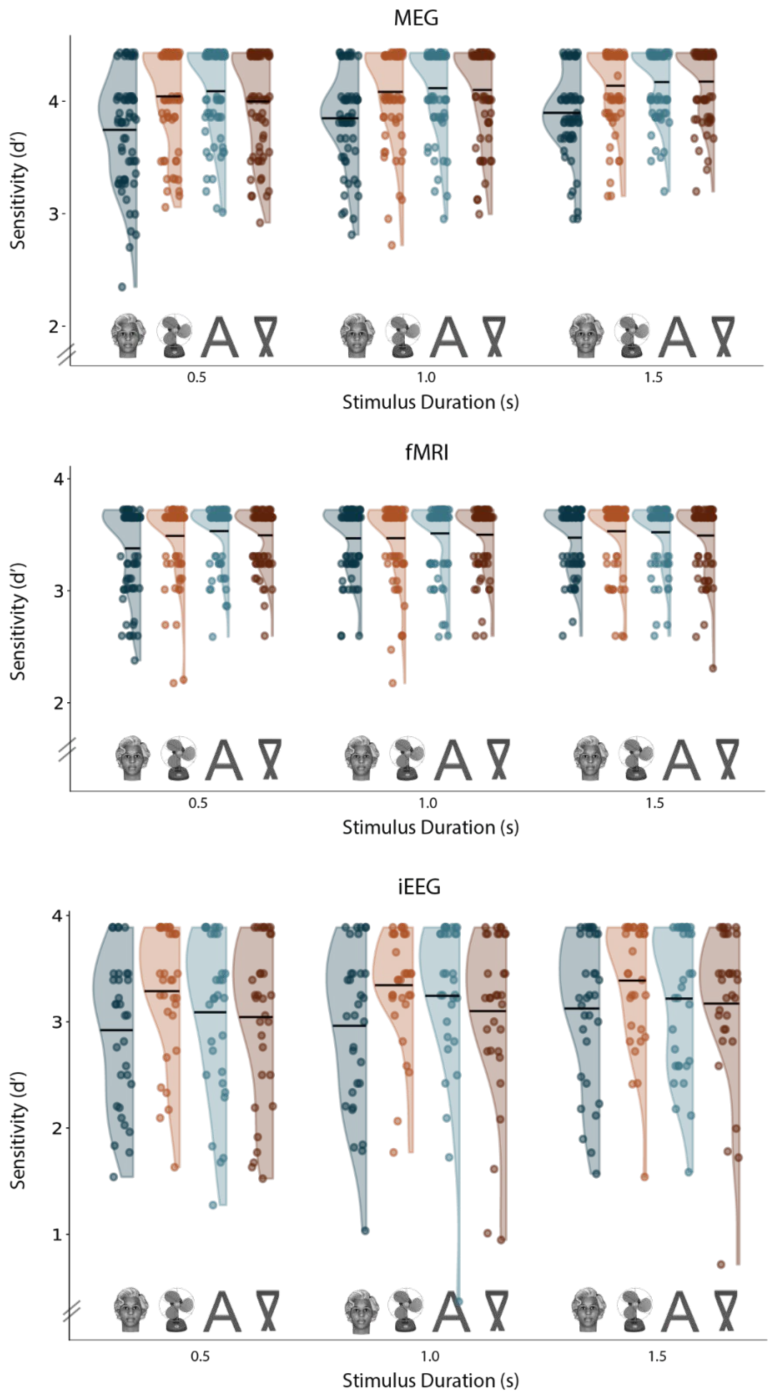

Supplementary Figure 2. d’ for MEG (N=65; upper panel), fMRI (N=73; middle panel) and iEEG (N=32; lower panel), for each one of the categories (faces, objects, letters, false fonts, marked with exemplary stimuli, and drawn in blue, orange, turquoise and brown, respectively) and each duration (horizontal axis; 0.5, 1.0 and 1.5 s). Each dot is an individual participant, plotted together with the overall distribution. Black horizontal lines depict the mean for each condition. LMM of d’ by modality, stimulus category, and duration showed a main effect of modality ($p<0.001$) and

category ($p<0.001$), and duration ($p<0.001$) as well as an interaction effect of modality and category ($p<0.001$). Face stimulus were created using FaceGen Modeler 3.1. Object stimulus are courtesy of Michael J Tart, Carnegie Melloni University, http://www.tarrlab.org

Importantly, there were no differences in performance, measured using d' between labs within each modality (iEEG: $F(2, 15.15)=0.11$, $p=1.000$ (all p values were Bonferroni corrected), $BF_{01}=20$; fMRI: $F(1, 70.87)=0.96$, $p=1.000$, $BF_{01}=20$; MEG: $F(1, 62.22)=0.08$, $p=1.000$, $BF_{01}=25$).

1.3 Hit rate analysis

To test whether the modality effect in sensitivity was affected by the different number of trials in each modality, we conducted an exploratory analysis where we ran the same model but with hit rate as a dependent variable, instead of d'. A small difference in hit rates (~3.5%) between different modalities was found ($p<0.001$, $BF_{10}=77.71$), such that hit rates in the iEEG modality (M=93.90, SD=12.29) were found to be lower than both fMRI (M=97.47, SD=7.26, $p<0.001$) and MEG (M=97.54, SD=5.20, $p<0.001$). No difference was found between the fMRI and MEG modalities ($p=1.000$), which further strengthens the conclusion that the higher d' values reported above for MEG stemmed from the higher number of trials (and the log-linear correction); see also False Alarms analysis below, where no difference was found between the MEG and fMRI samples). In addition, hit rates did not differ between different categories ($p=1.000$), and no interactions were found (p values range between 0.330 and 1.000). No difference in hit rates was found between labs within each modality (fMRI: $F(1, 72.81)=0.23$, $p=0.633$, $BF_{01}=25$; MEG: $F(1, 54.69)=3.99$, $p=0.356$, $BF_{01}=4.35$; iEEG: $F(2, 8.91)=0.71$, $p=1.000$, $BF_{01}=16.67$). Stimulus duration did modulate hit rates ($p<0.001$, $BF_{10}=66.98$), with the short duration stimuli (M=96.05, SD=8.92) showing slightly lower hit rates than both the intermediate (M=96.91, SD=7.78; $p=0.041$) and the long duration stimuli (M=97.51, SD=6.98; $p<0.001$); the intermediate and long duration stimuli did not differ from one another in terms of hit rates ($p=0.102$).

1.4 False Alarm analysis

A logistic mixed model was used to test if false alarms were modulated by task relevance (Relevant, Irrelevant), stimulus category (Faces, Objects, Letters, False Fonts), or modality (iEEG, fMRI, MEG), defined as fixed effects. Participant was again defined as a random effect.[1] As expected, task relevance affected false alarms ($\chi^2(1)=241.05$, $p<0.001$, $BF_{10}=3.89 \times 10^{51}$), such that more false alarms were found in the task relevant condition (M=1.73%, SD=3.92) compared with the task irrelevant one (M=0.73%, SD=3.90; $p<0.001$, Supplementary Figure 3). This finding reinforces the effectiveness of our task manipulation.

A main effect of category was also found ($\chi^2(3)=182.23$, $p<0.001$, $BF_{10}=3.11 \times 10^{36}$), such that faces (M=1.78%, SD=4.01) led to higher false alarm rates compared with letters (M=0.97%, SD=3.71, $p<0.001$) and objects (M=0.98%, SD=3.59, $p<0.001$), but not compared to false fonts

[1] Here, we first ran the model with Item as a random effect, as preregistered, yet the model failed to converge, probably due to the very low number of false alarms.

(M=1.43%, SD=4.63, p=0.130), which in turn also evoked more false alarms than letters (p=0.002). Modality was also found to affect false alarms ($\chi^2(2)=64.05$, p<0.001, $BF_{10}=7.33 \times 10^{11}$), with iEEG (M=4.22%, SD=8.22) patients having higher false alarm rates compared to both MEG (M=0.63%, SD=0.62, p<0.001) and fMRI (M=0.59%, SD=0.54, p<0.001) participants, which, in turn, did not differ from each other (p=0.480). In addition, the interaction between category and modality was significant ($\chi^2(6)=35.61$, p<0.001, $BF_{10}=2.75 \times 10^3$) such that in the fMRI sample there were no differences between the stimulus categories (p values range between 0.070 and 1.000); In the iEEG sample, false fonts (M=4.95%, SD=9.94) led to more false alarms than both letters (M=3.48%, SD=8.08; p=0.005) and objects (M=3.14%, SD=7.91; p<0.001). In addition, faces had significantly higher false alarm rates compared to both letters (p=0.018) and objects (p<0.001), but did not differ from false fonts (p=1.000). Objects and letters did not differ (p=1.000). This result strengthens the interpretation that the MEG modality was driving the category face effect, with faces (M=1.18%, SD=0.87) showing a higher false alarm rate compared to letters (M=0.31%, SD=0.53; p<0.001), false fonts (M=0.59%, SD=0.77; p=0.013) and objects (M=0.45%, SD=0.70; p<0.001). Notably, the difference between false fonts and letters in the MEG modality was found to be significant as well (p=0.002; false fonts vs. objects p=1.000, letters vs. objects p=0.088).

The interaction between modality and task relevance was also significant ($\chi^2(2)=29.48$, p<0.001, $BF_{10}=2.28 \times 10^4$), such that while in all modalities participants made more false alarms to task relevant stimuli, the magnitude of such differences changed between modalities (iEEG relevant M=5.21%, SD=8.12, irrelevant M=2.98%, SD=8.67; MEG relevant M=0.92%, SD=0.65, irrelevant M=0.27%, SD=0.68; fMRI relevant M=0.93%, SD=0.81, irrelevant M=0.16%, SD=0.37; p<0.001 in all modalities). The interaction between stimulus category and relevance was also significant ($\chi^2(3)=56.15$, p<0.001, $BF_{10}=2.32 \times 10^9$) with no difference in false alarm rate between different categories when task irrelevant (all p-values range between 0.173 and 1.000). When task relevant, faces evoked higher false alarm rates (M=2.65%, SD=5.00) compared to all other stimuli (p<0.001 for all contrasts; false fonts: M=1.94%, SD=5.07, letters: M=1.17%, SD=3.59, objects: M=1.17%, SD=3.51). In addition, false fonts had higher false alarm rates compared to both letters and objects (p<0.001 for both). Letters and objects did not differ from each other (p=1.000). The triple interaction was not significant (p=0.696, $BF_{01}=11.11$).

Akin to the d' analysis, no difference in false alarm rates was found between labs within each modality (fMRI: $\chi^2(1)=1.26$, p=1.000, $BF_{01}=12.5$; MEG: $\chi^2(1)=3.20$, p=0.517, $BF_{01}=5$; iEEG: $\chi^2(2)=1.34$, p=1.000, $BF_{01}=14.29$).

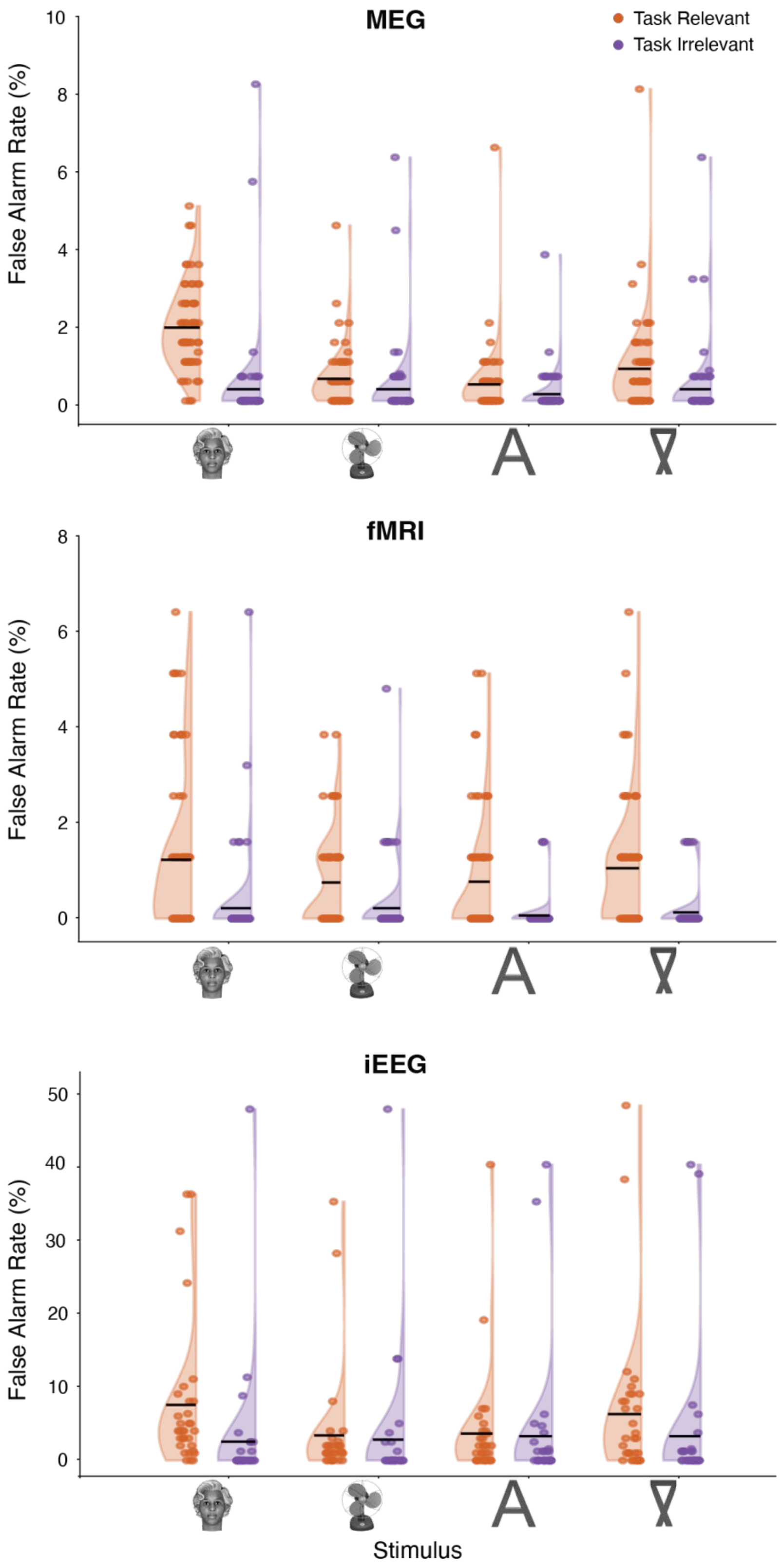
MEG
Task Relevant
Task Irrelevant
False Alarm Rate (%)
0
2
4
6
8
10
fMRI
False Alarm Rate (%)
0
2
4
6
8
A
iEEG
False Alarm Rate (%)
0
10
20
30
40
50
Stimulus

Supplementary Figure 3. FAs for MEG (N=65; upper panel), fMRI (N=73; middle panel) and iEEG (N=32; lower panel), for each one of the categories (faces, objects, letters, false fonts) in the task relevant (orange) and task irrelevant (purple) conditions. Each dot is an individual participant, plotted together with the overall distribution. Note the different scales for the different modalities, with some iEEG patients having a substantially larger number of false alarms compared to the other modalities. Black horizontal lines depict the mean for each condition. A logistic mixed-effects model (two-tailed) of false alarms by modality, task relevance and stimulus category showed a main effect of modality ($p<0.001$), task relevance ($p<0.001$) and category ($p<0.001$), as well as interactions between modality and category ($p<0.001$), modality and task relevance ($p<0.001$), and between task relevance and category ($p<0.001$). Face stimulus were created using FaceGen Modeler 3.1. Object stimulus are courtesy of Michael J Tart, Carnegie Melloni University, http://www.tarrlab.org.

1.5 Reaction times analysis

A linear mixed model was used to test if reaction times for hits were modulated by stimulus category (Faces, Objects, Letters, False Fonts), stimulus duration (0.5 s, 1.0 s, 1.5 s) or modality (iEEG, fMRI, MEG). These factors were defined as fixed effects, and participant and item were defined as random effects. A main effect of category was found ($F(3, 221.3)=40.23$, $p<0.001$, $BF_{10}=2.95x10^{17}$), with letters - which are arguably the easiest and most automated to identify - evoking the fastest responses (M=0.58s, SD=0.20), compared with faces (M=0.63, SD=0.21, $p<0.001$), objects (M=0.62, M=0.19, $p<0.001$) and false fonts (M=0.64, SD=0.21, $p<0.001$; Supplementary Figure 4). In addition, reaction times for objects were faster compared to both faces and false fonts ($p<0.001$ for both). Faces and false fonts did not differ from each other ($p=1.000$).

A main effect of modality was also found ($F(2, 164.9)=14.99$, $p<0.001$, $BF_{10}=2.44x10^{3}$), with the MEG sample showing shorter reaction times (M=0.59s, SD=0.06) than the fMRI (M=0.67, SD=0.11; $p<0.001$) and iEEG samples (M=0.65, SD=0.10; $p=0.006$). Reaction times between the iEEG and fMRI samples were not found to be different ($p=0.693$). An interaction between modality and category was also found ($F(6, 14480.8)=3.45$, $p=0.014$, though $BF_{10}=1.23$), with MEG showing a letter advantage (for all contrasts, $p<0.001$; faces: M=0.60, SD=0.08; false fonts: M=0.60, SD=0.06; objects: M=0.59, SD=0.07; letters: M=0.56, SD=0.07), the rest of the contrasts were not significant in the MEG sample (objects vs. false fonts $p=0.961$, faces vs. false fonts=1.000, faces vs. objects $p=1.000$). In the iEEG sample, the letter advantage (M=0.62, SD=0.11) was found when compared to faces (M=0.67, SD=0.12; $p<0.001$) and false fonts (M=0.66, SD=0.12; $p<0.001$), but not to objects (M=0.65, SD=0.10; $p=0.094$). Faces did not differ from false fonts ($p=1.000$) and objects ($p=0.393$), and objects did not differ from false fonts ($p=0.760$). In the fMRI data, faces (M=0.69, SD=0.13) were different from both letters (M=0.64, SD=0.11) and objects (M=0.65, SD=0.11; $p<0.001$ in both cases), and the same was found for false fonts (M=0.70, SD=0.13; again, $p<0.001$). No other effects survived the Bonferroni correction (p values range between 0.302 ($BF_{01}=16.67$) and 1.000 ($BF_{01}=100$)).

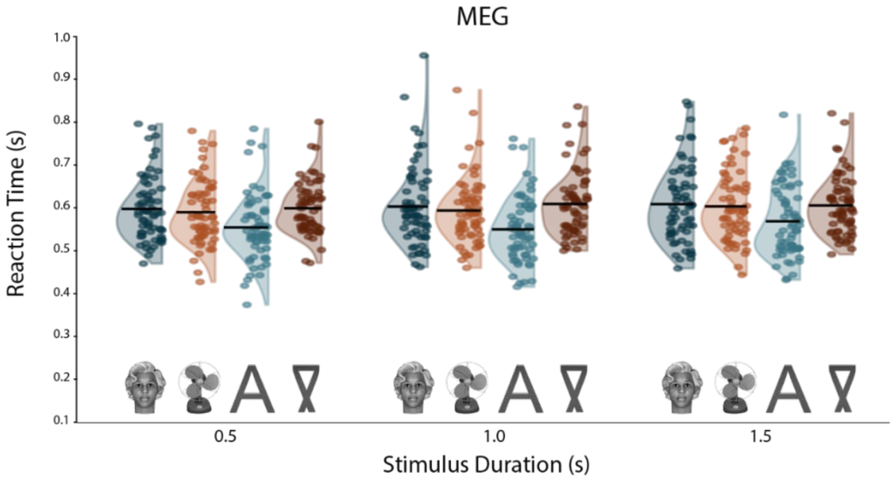
MEG
Reaction Time (s)
1.0
0.9
0.8
0.7
0.6
0.5
0.4
0.3
0.2
0.1
0.5
1.0
1.5
Stimulus Duration (s)

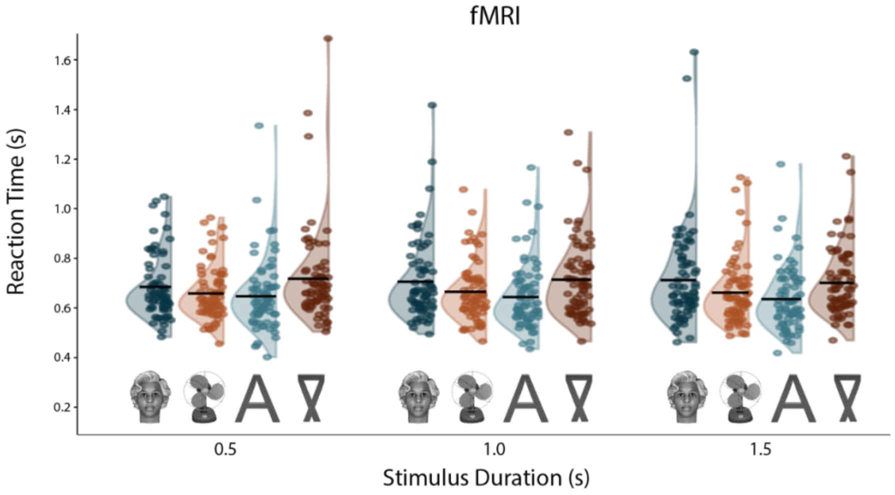
fMRI
Reaction Time (s)
1.6
1.4
1.2
1.0
0.8
0.6
0.4
0.2
0.5
1.0
1.5
Stimulus Duration (s)

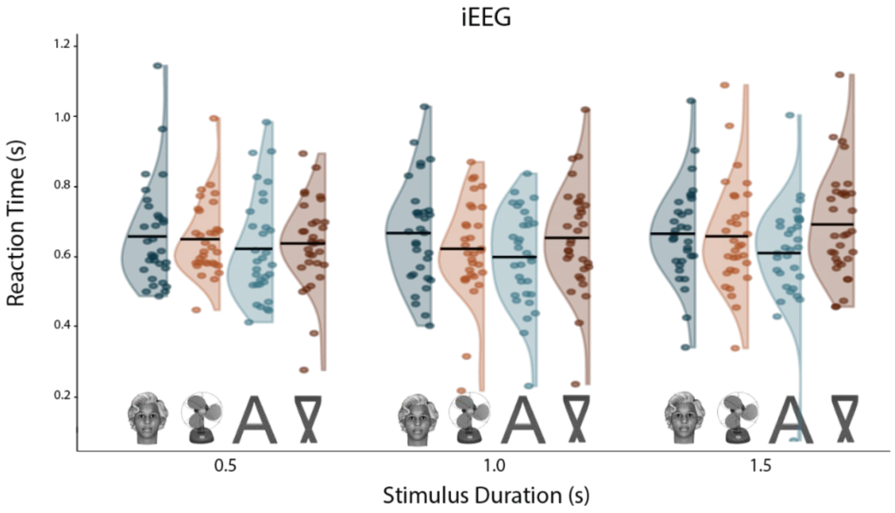
iEEG
Reaction Time (s)
1.2
1.0
0.8
0.6
0.4
0.2
0.5
1.0
1.5
Stimulus Duration (s)

Supplementary Figure 4. Reaction times for the different modalities (MEG: N=65; fMRI: N=73; iEEG: N=32), conditions and durations. The same conventions as in Supplementary Figure 2 are used. A linear mixed-effects model (two-tailed) of reaction time for hits by modality, stimulus duration and stimulus category showed a main effect of modality ($p<0.001$) and category ($p<0.001$), as well as an interaction between modality and category ($p=0.014$). Face stimulus were created using FaceGen Modeler 3.1. Object stimulus are courtesy of Michael J Tart, Carnegie Melloni University, http://www.tarrlab.org.

2. Eye movements analyses

Analyses were done with R (4.3.1, ordinal 2023.12.4, tidyR 1.3.0, dplyr 1.1.4, lmerTest 3.1.3, bayestestR 0.14.0, emmeans 1.10.4) and Python (3.9, numpy 1.21.2, pandas 1.5.2, scipy 1.7.1, pycircstat 0.0.2, astropy 4.3.post1, seaborn 0.12.1, matplotlib 3.6.2, statsmodels 0.14.0, matlab.engine 9.11.19). Saccade analysis features are based on Engbert & Mergenthaler, 2006, but the vfac parameter is based on Engbert & Kliegl, 2003.

Pre-registered analyses

Eye movement patterns were processed in Python (v. 3.9) and analyzed in R (4.3.1) with respect to four dependent variables: fixation distance from screen center, saccade amplitude, number of blinks and pupil size. For each such variable, we asked how it was modulated by task relevance (Relevant, Irrelevant) and category (Faces, Objects, Letters, False fonts). These were defined as fixed effects, while participant and item were defined as random effects in a linear mixed model. We first focused on the first 0.5 s of the stimulus, that were shared for all three durations. Then, to explore how these variables changed over time, we only analyzed the long duration stimuli (1.5 s), and added time window (0-0.5 s / 0.5-1.0 s / 1.0-1.5 s) as a fixed effect. Thus, 8 models were run overall (4 dependent variables X 2 analyses). This analysis was conducted for all sites using EyeLink; NYU data, which was recorded using Tobii, is only included in the heatmaps (Supplementary Figure 15), but not in any of the other analyses, due to the low quality of the data. Notably, eleven participants were excluded from this analysis: three participants due to not having eye tracking data to begin with (2 iEEG patients, 1 MEG participant) and eight for having data of insufficient quality (5 iEEG patients, 1 MEG participant, and 2 fMRI participants).

2.1 First 0.5 s: Fixation distance from center

Participants were very good at maintaining fixation. Importantly, their ability to maintain fixation within the first 0.5 s, as assessed by the median distance of fixation from the screen center (Supplementary Figure 5), was not affected by task relevance ($F(1, 59012)=0.25$, $p=1.000$, $BF_{01}=333.33$). Stimulus category did affect fixations ($F(3, 59021)=4.59$, $p=0.010$, though $BF_{01}=3.45$), with lower distance from the center for faces ($M=1.61$, $SD=1.26$) compared to false fonts ($M=1.64$, $SD=1.31$; $p=0.003$; the rest of the comparisons were not significant and ranged between 0.145 and 1.000). The interaction between task relevance and category was also not significant ($F(3, 59010)=0.31$, $p=1.000$, $BF_{01}=333.33$).

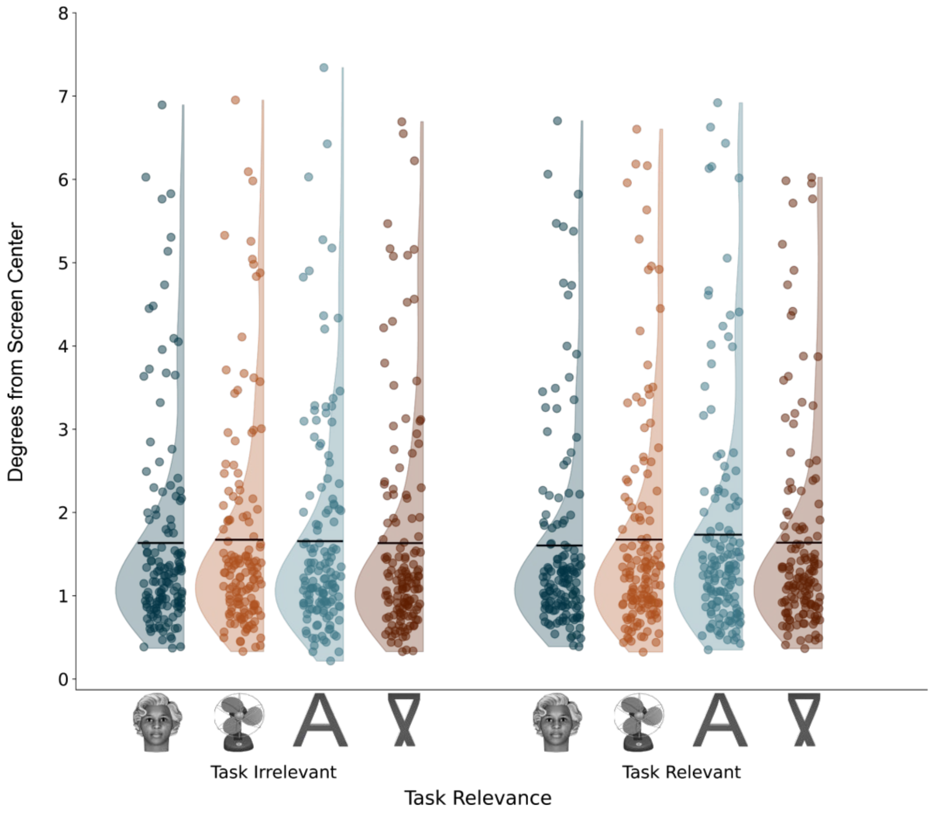

Supplementary Figure 5. Mean distance of fixations from the center, across all modalities (MEG: N=63; fMRI: N=71; iEEG: N=9) and durations, per stimulus category and task relevance. The same conventions as in Supplementary Figure 2 are used. A linear mixed-effects model (two-tailed) of the distance by task relevance and category showed a main effect of category (p=0.010) but not of task relevance (p=1.000) nor their interaction (p=1.000). Face stimulus were created using FaceGen Modeler 3.1. Object stimulus are courtesy of Michael J Tart, Carnegie Melloni University, http://www.tarrlab.org.

2.2 First 0.5 s: Number of blinks

Overall, the number of blinks was very low, with about one blink per trial on average (M=1.10, SD=1.08; Supplementary Figure 6). Small differences were nevertheless found between the conditions. Namely, a main effect was found for category ($F(3, 188)=31.11$, $p<0.001$, $BF_{10}=4.28x10^{12}$), such that faces evoked fewer blinks on average (M=0.14, SD=0.15) than objects (M=0.15, SD=0.17, $p<0.001$), letters (M=0.16, SD=0.16, $p<0.001$) and false fonts M=0.16, SD=0.17, $p<0.001$). The rest of the categories did not differ from one another (p values range between 0.099 and 1.000). A main effect was found for task relevance ($F(1, 120346)=90.75$, $p<0.001$, $BF_{10}=5.73x10^{17}$), with less blinks in the task relevant (M=0.14, SD=0.16) than the task irrelevant (M=0.16, SD=0.17, $p<0.001$) condition. An interaction between the two factors was also found ($F(3, 120342)=5.28$, $p=0.004$, though $BF_{10}=0.77$). Post-hoc comparisons showed that while for the task relevant condition, faces differed from all conditions ($p<0.001$ for the three comparisons, others were not significant and ranged between 0.477 and 1.000), in the irrelevant

condition, faces differed only from letters and false fonts (p<0.001 for both), but not from objects (p=0.155). In addition, objects also differed from both false fonts (p<0.001) and letters (p=0.023). False fonts and letters did not differ from each other (p=1.000).

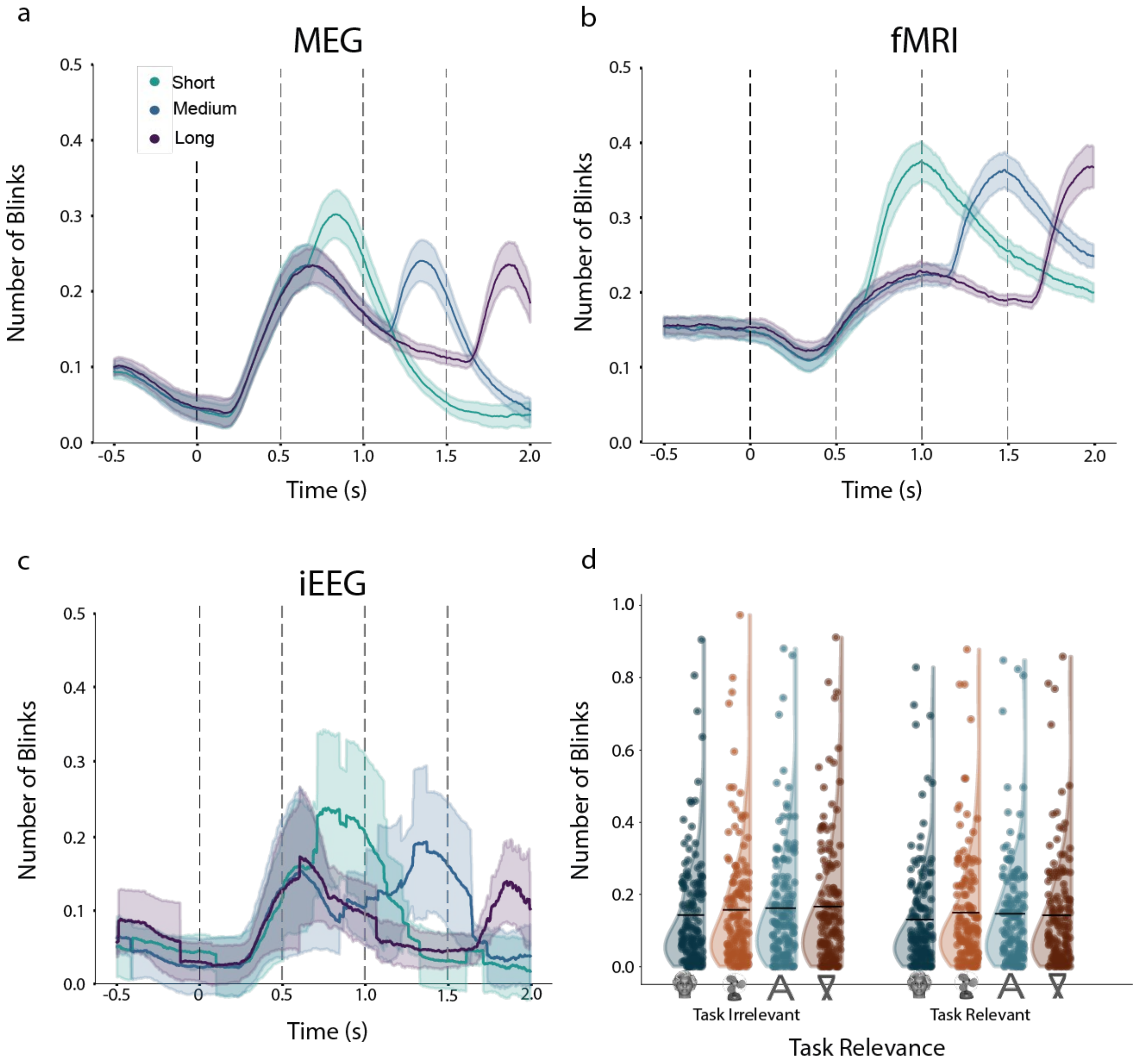

Supplementary Figure 6. a-c: Average number of blinks over time across participants for the three durations (short, medium and long, in green, blue and purple, respectively), for the three modalities (a: MEG: N=63; b: fMRI: N=71; c: iEEG: N=9). The black dashed line marks the onset of the stimulus, while gray dashed lines mark the three offsets. Error bars depict 95% CIs. **d**. Distributions of the average number of blinks per participant in the first 0.5 s of all trials (represented by the dots), across all modalities and durations, broken down by stimulus category (faces, objects, letters, false-fonts; horizontal axis) and task conditions (irrelevant on the left, and relevant on the right). A linear mixed-effects model (two-tailed) of the number of blinks by task relevance and category showed a main effect for category (p<0.001) and task relevance (p<0.001)

as well as their interaction (p=0.004). Face stimulus were created using FaceGen Modeler 3.1. Object stimulus are courtesy of Michael J Tart, Carnegie Melloni University, http://www.tarrlab.org.

2.3 First 0.5 s: Saccade amplitude

We further investigated the amplitudes of the saccades within the first 0.5 s of stimulus presentation. A main effect of stimulus category was found ($F(3, 211)=5.87$, $p=0.002$, though $BF_{10}=1.31$; Supplementary Figure 7d). This stemmed from the letter stimuli evoking greater amplitudes (M=1.40, SD=1.72) compared to faces (M=1.25, SD=1.33, $p<0.001$), false fonts (M=1.27, SD=1.32, $p=0.002$) and objects (M=1.24, SD=1.13, $p=0.006$). Other differences between categories were not found ($p=1.000$ for all the other comparisons). In addition, no effects were found for either task relevance ($F(1, 35980)=0.06$, $p=1.000$, $BF_{01}=333.33$) nor the interaction between relevance and category ($F(3, 36025)=1.28$, $p=0.840$, $BF_{01}=200$). In addition to saccade amplitudes, we also report the number of saccades over time in the different modalities (Supplementary Figure 7).

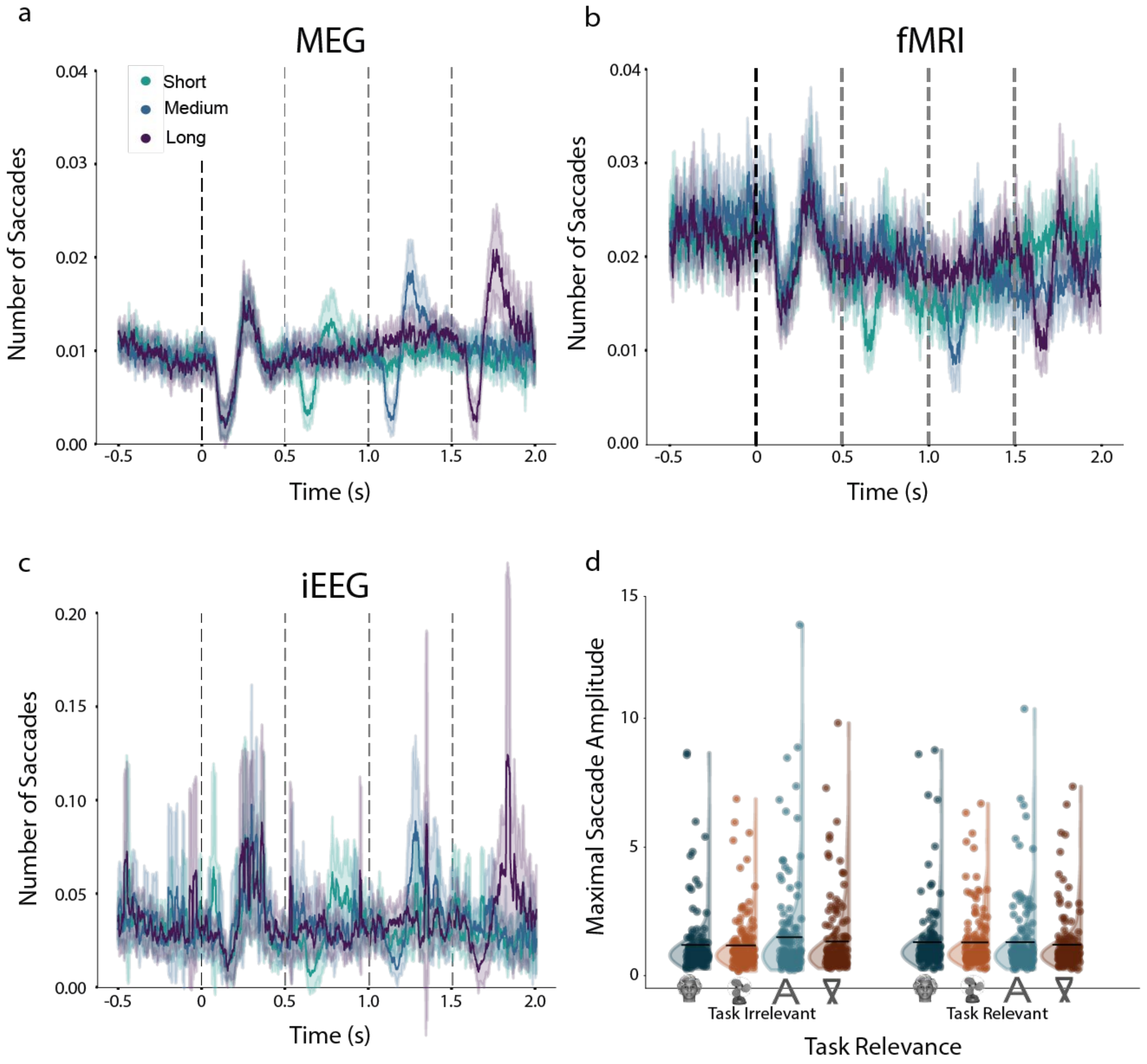

Supplementary Figure 7. a-c. Number of saccades over the trial, separately for the different modalities (MEG: N=63; fMRI: N=71; iEEG: N=9). Error bars depict 95% CIs. **d.** Distributions of the maximal saccade amplitude per participant in the first 500 ms of all trials. The same conventions as in Supplementary Figure 6 are used. A linear mixed-effects model (two-tailed) of the amplitude by task relevance and category showed a main effect of category ($p<0.001$), but not of task relevance ($p=1.000$) nor their interaction ($p=0.840$). Face stimulus were created using FaceGen Modeler 3.1. Object stimulus are courtesy of Michael J Tart, Carnegie Melloni University, http://www.tarrlab.org.

2.4 First 0.5 s: Pupil size

Pupil size was modulated only by the interaction between stimulus category and task relevance (Supplementary Figure 8; $F(3, 112912)=3.83$, $p=0.028$, though $BF_{01}=10$); However,

post hoc comparisons yielded no differences between the categories both when task relevant and irrelevant (p values range between 0.133 and 1.000 for the task irrelevant stimuli, and between 0.230 and 1.000 for the task relevant stimuli). The other main effects were not significant (p=1.000 and BF_{01}=333.33 for both).

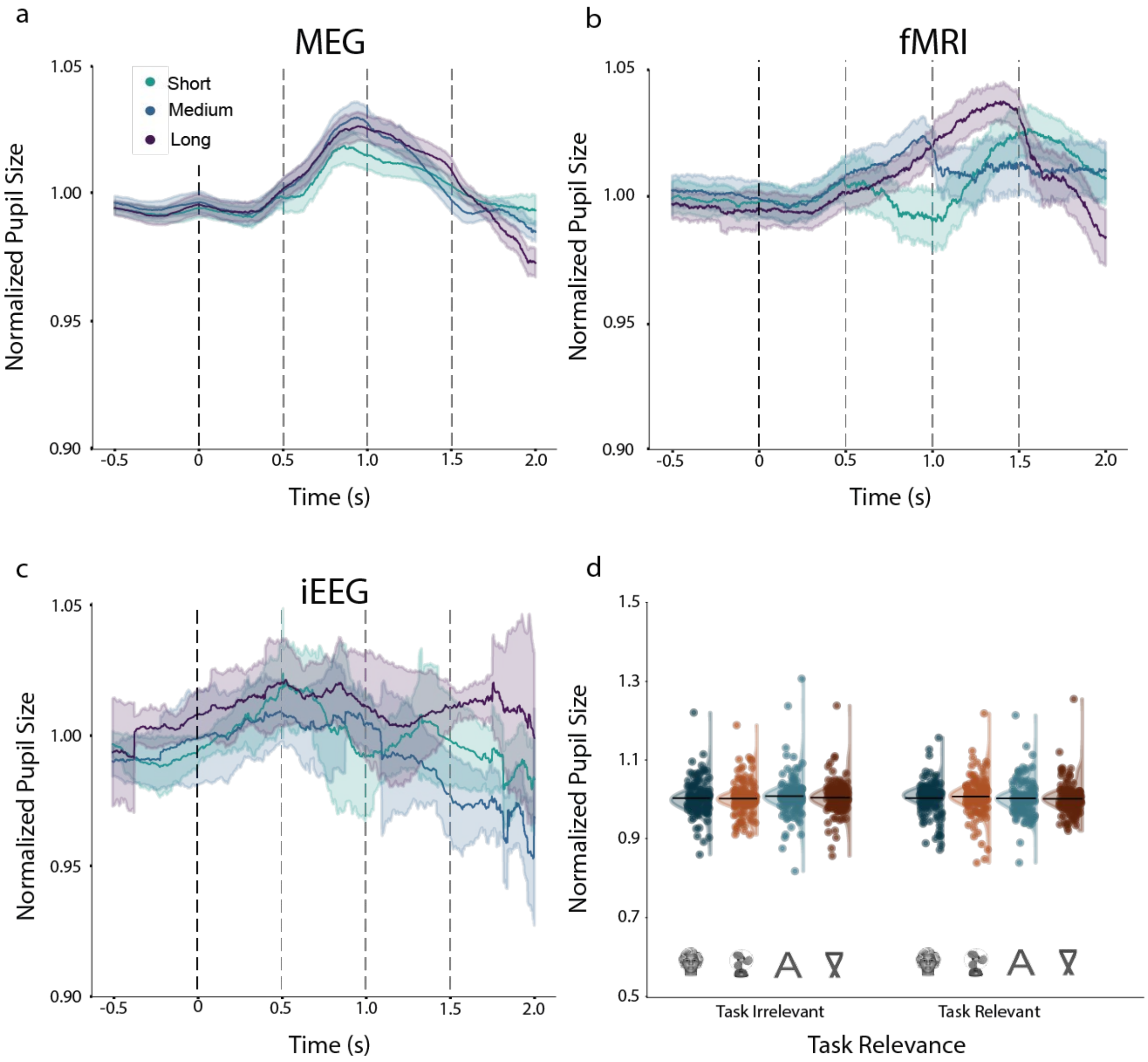

Supplementary Figure 8. a-c. Averaged standardized pupil size over the trial, separately for the different modalities (MEG: N=63; fMRI: N=71; iEEG: N=9). Error bars depict 95% CIs. **d.** Distributions of the standardized pupil size per participant in the first 500 ms of all trials. The same conventions as in Supplementary Figure 6 are used. A linear mixed-effects model (two-tailed) of pupil size by task relevance and category showed no main effects (p=1.000 for both), but an

interaction effect (p=0.028). Face stimulus were created using FaceGen Modeler 3.1. Object stimulus are courtesy of Michael J Tart, Carnegie Melloni University, http://www.tarrlab.org.

2.5 Long trials analysis: Fixation distance from center

When assessing fixation distance from the center of the screen for the 1.5 s stimuli, a main effect of time window was found ($F(2, 57130)=578.05$, $p<0.001$, $BF_{10}=3.27 \times 10^{245}$; Supplementary Figure 9), such that fixation distance differed between all three time windows, growing more distant later in time: first time window: M=1.64 dva, SD=1.36); second time window: M=1.65, SD=1.34; third time window: M=1.71, SD=1.32; $p<0.001$ for all comparisons). No other effect survived Bonferroni correction (p values range between 0.217 ($BF_{01}=33.33$) and 1.000 ($BF_{01}=166.67$)).

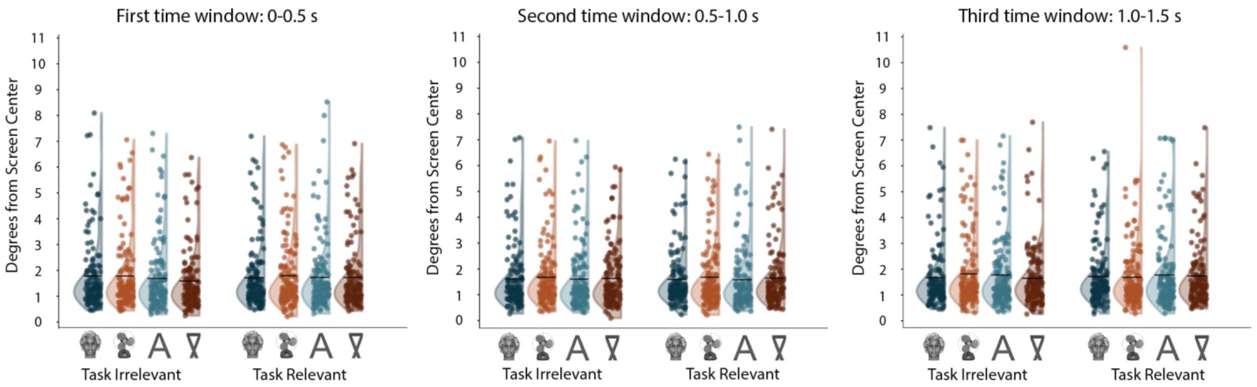

Supplementary Figure 9. The averaged distance of fixations from the screen center (in degrees of visual angle) for the first (left), second (middle) and third (right) time windows, in each category (horizontal axis) for task irrelevant (left) and relevant (right) stimuli. Each dot is an individual participant, plotted together with the overall distribution (N=143). The black horizontal lines mark the averaged value per condition. For illustration purposes, we excluded one outlier participant whose averaged gaze was highly affected by 2 trials where they diverted their gaze away from the center (M=16.80). This participant was not excluded from the analysis. A linear mixed-effects model (two-tailed) of the distance by time window, task relevance and category showed only an effect of time window ($p<0.001$). Face stimulus were created using FaceGen Modeler 3.1. Object stimulus are courtesy of Michael J Tart, Carnegie Melloni University, http://www.tarrlab.org.

2.6 Long trials analysis: Number of blinks

Here, despite the overall very low number of blinks during long stimulus duration trials (M=1.15, SD=1.10), many differences were found between the conditions (Supplementary Figure 10). For convenience, all effects are summarized in Supplementary Table 1.

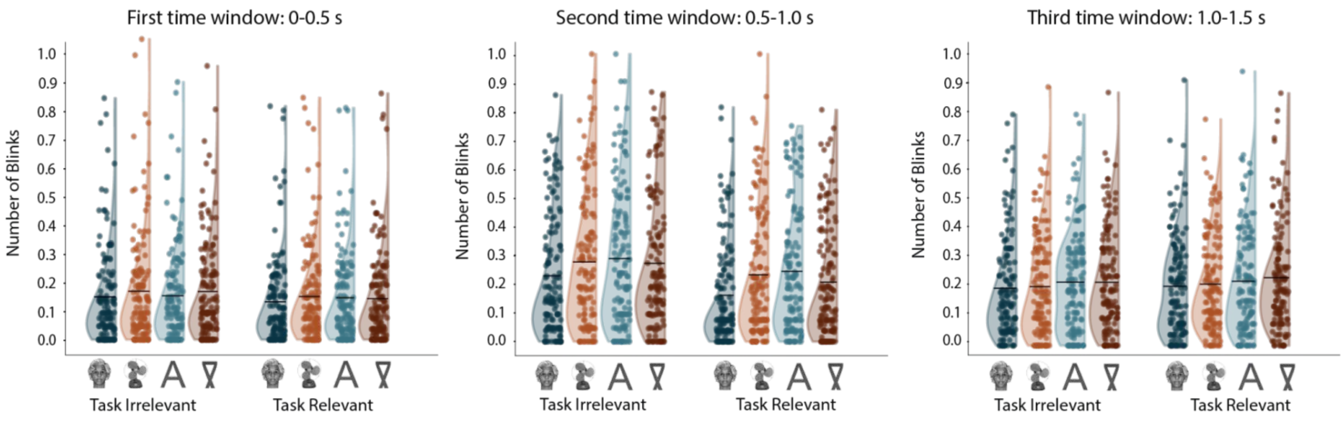

Supplementary Figure 10. Averaged number of blinks for the first (left), second (middle) and third (right) time window, in each category (N=143). The same conventions are used as in Supplementary Figure 9. Results of the linear mixed-effects model (two-tailed) of the number of blinks by time window, task relevance and category are summarized in Supplementary Table 1. Face stimulus were created using FaceGen Modeler 3.1. Object stimulus are courtesy of Michael J Tart, Carnegie Melloni University, http://www.tarrlab.org.

	df	F	Adjusted p value
**Category**	**(3, 123183)**	**49.62**	**<0.0001 (BF_{10}=1.95x10^{28})**
Category: face vs. false font	Inf		<0.0001
Category: face vs. letter	Inf		<0.0001
Category: face vs. object	Inf		<0.0001
Category: false font vs. letter	Inf		1.000
Category: false font vs. object	Inf		0.710
Category: letter vs. object	Inf		0.024
**Task Relevance**	**(1, 123185)**	**101.90**	**<0.0001 (BF_{10}=1.59x10^{20})**
Task Relevance: irrelevant vs. relevant	Inf		<0.0001
**Time Window**	**(2, 123180)**	**907.97**	**<0.0001 (BF_{10}=inf)**
Time Window: first vs. second	Inf		<0.0001

Time Window: second vs. third	Inf		<0.0001
Time Window: first vs. third	Inf		<0.0001
**Category x Task Relevance**	**(3, 123187)**	**1.27**	**1.000 (BF_{01}=200)**
**Time Window x Category**	**(6, 123180)**	**13.28**	**<0.001 (BF_{10}=2.28x10^{11})**
First: face vs. false font	Inf		0.002
First: face vs. letter	Inf		0.015
First: face vs. object	Inf		0.020
First: false font vs. letter	Inf		1.000
First: false font vs. object	Inf		1.000
First: letter vs. object	Inf		1.000
Second: face vs. false font	Inf		<0.0001
Second: face vs. letter	Inf		<0.0001
Second: face vs. object	Inf		<0.0001
Second: false font vs. letter	Inf		0.012
Second: false font vs. object	Inf		1.000
Second: letter vs. object	Inf		0.215
Third: face vs. false font	Inf		0.001
Third: face vs. letter	Inf		0.003
Third: face vs. object	Inf		1.000
Third: false font vs. letter	Inf		1.000
Third: false font vs. object	Inf		0.016
Third: letter vs. object	Inf		0.031
**Time Window x Task Relevance**	**(2, 123180)**	**69.02**	**<0.001 (BF_{10}=8.64x10^{26})**
First: irrelevant vs. relevant	Inf		<0.001
Second: irrelevant vs. relevant	Inf		<0.001

Third: irrelevant vs. relevant	Inf		0.022
**Time Window x Category x Task Relevance**	**(6, 123180)**	**2.19**	**0.288 (BF_{01}=50)**

Supplementary Table 1. Statistics for all comparisons made in the analysis of number of blinks for long duration stimuli, with the factors: category, task relevance, and time window. Main effects and interactions are highlighted in bold, and significant ones are followed by post-hoc tests.

2.7 Long trials analysis: Saccade amplitude

A main effect of time window was found on saccade amplitudes (F(2, 35796)=26.63, p<0.001, BF_{10}=3.43x10^8; Supplementary Figure 11), such that the saccade amplitude in the first time window (M=1.23, SD=1.60) was lower than in both the second (M=1.39, SD=1.86, p<0.001) and the third time windows (M=1.35, SD=1.61; p<0.001), while these latter two windows did not differ from each other (p=1.000). The interaction between category and task relevance was also significant (F(3, 35575)=4.31, p=0.034, though BF_{01}=5.05), such that when task irrelevant, letters (M=1.47, SD=2.23) differed from both faces (M=1.25, SD=1.57; p=0.002) and objects (M=1.23, SD=1.38, p=0.044). The rest of the contrasts were not significant (p value ranges between 0.106 and 1.000). When task relevant, saccade amplitudes were lower for faces (M=1.23, SD=1.12) than objects (M=1.56, SD=2.20, p=0.034). The rest of the contrasts did not differ (p values range between 0.277 and 1.000).

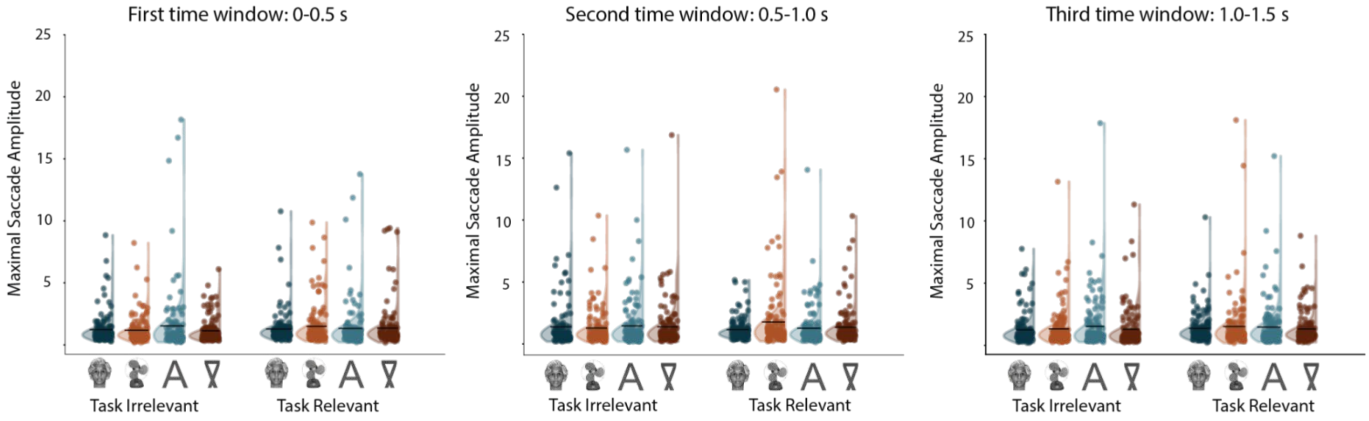

Supplementary Figure 11. The averaged maximal saccade amplitude (in arbitrary units) for the first, second and third time window (N=143). The same conventions are used as in Supplementary Figure 9. A linear mixed-effects model (two-tailed) of saccade amplitude by time window, task relevance and category showed only an effect of time window (p<0.001) and an interaction between category and task relevance (p=0.034). Face stimulus were created using FaceGen Modeler 3.1. Object stimulus are courtesy of Michael J Tart, Carnegie Melloni University, http://www.tarrlab.org.

2.8 Long trials analysis: Pupil size

Though only an interaction effect was found for standardized pupil size when examining the first 0.5 s post-stimulus (with no significant post-hoc differences; see analysis above), several differences emerged when only the long stimulus duration trials were analyzed (Supplementary Figure 12). A main effect of task relevance was found ($F(1, 105556)=81.26$, $p<0.001$, $BF_{10}=4.73x10^{15}$), with smaller pupil sizes for task irrelevant (M=1.01, SD=0.07) compared to task relevant stimuli (M=1.02, SD=0.07; $p<0.001$). Additionally, a main effect of time window was found ($F(2, 105719)=136.34$, $p<0.001$, $BF_{10}=1.30x10^{56}$), with pupil size in the first time window (M=1.00, SD=0.06) being smaller than both the second (M=1.02, SD=0.07; $p<0.001$) and the third windows (M=1.02, SD=0.07; $p<0.001$), with no difference between the second and third windows ($p=0.443$). The interaction between task relevance and time window was also significant ($F(2, 105642)=58.13$, $p<0.001$, $BF_{10}=1.61x10^{22}$). Post hoc analysis revealed that while in the first time window there was no difference in pupil size between task relevant and irrelevant stimuli ($p=0.132$), pupil size was smaller for task irrelevant stimuli both in the second (irrelevant: M=1.01, SD=0.07, relevant: M=1.02, SD=0.07; $p<0.001$) and third (irrelevant: M=1.01, SD=0.07, relevant: M=1.04, SD=0.07; $p<0.001$) time windows. The interaction between time window and stimulus category was also significant ($F(6, 105638)=3.51$, $p=0.013$, though $BF_{10}=0.53$), with no differences between stimuli in the first time window ($p=1.000$ for all comparisons). In the second time window, faces (M=1.01, SD=0.06) led to smaller pupil sizes than both false fonts (M=1.02, SD=0.07; $p<0.001$) and letters (M=1.02, SD=0.06; $p=0.013$; other p values range between 0.099 and 1.000). In the third time window, the only difference in pupil size was between faces (M=1.02, SD=0.06) and false fonts (M=1.03, SD=0.07, $p=0.037$). All other factors were not significant. Supplementary Table 2 summarizes the means, SDs, and statistics for the different conditions.

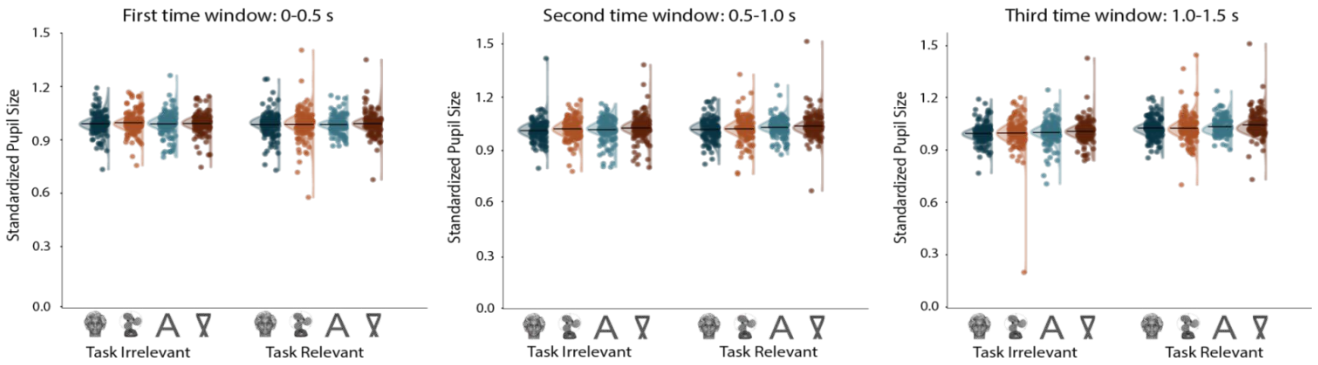

Supplementary Figure 12. Standardized pupil size for the different time windows (N=143). The same conventions as in Supplementary Figure 9 are used. The results of the linear mixed-effects model (two-tailed) of pupil size by time window, task relevance and category are summarized in Supplementary Table 2. Face stimulus were created using FaceGen Modeler 3.1. Object stimulus are courtesy of Michael J Tart, Carnegie Melloni University, http://www.tarrlab.org.

	df	F	Adjusted p value
Category	(3, 228)	3.65	0.093 (BF_{01}=14.29)
Task Relevance	(1, 105556)	81.26	<0.0001 (BF_{10}=4.73x10^{15})
Task Relevance: irrelevant vs. relevant	Inf		<0.0001
Time Window	(2, 105719)	136.34	<0.0001 (BF_{10}=1.30x10^{56})
Time Window: first vs. second	Inf		<0.0001
Time Window: first vs. third	Inf		<0.0001
Time Window: second vs. third	Inf		0.443
Category x Task Relevance	(3, 105565)	2.86	0.247 (BF_{01}=33.33)
Category x Time Window	(6, 105638)	3.51	0.013 (BF_{10}=0.53)
Time Window x Task Relevance	(2, 105642)	58.13	<0.0001 (BF_{10}=1.61x10^{22})
First: irrelevant vs. relevant	Inf		0.132
Second: irrelevant vs. relevant	Inf		<0.0001
Third: irrelevant vs. relevant	Inf		<0.0001
Time Window x Category x Task Relevance	(6, 105637)	0.43	1.000 (BF_{01}=333.33)

Supplementary Table 2. Statistics for all comparisons made in the analysis of pupil size for the long duration stimuli, with the factors: category, task relevance, and time window. The same conventions as in Supplementary Table 1 are used.

3. Control experiment: Surprise memory test

In our main experiment, the stimuli were presented at high contrast, in isolation, at the center of fixation, for long durations. However, given the low prevalence of the targets and the paucity of the display (a stimulus was presented approximately every 2.2 seconds), it could be argued that participants may have missed some stimuli, not being consciously aware of some of them. This could be especially true for the task irrelevant stimuli, on which participants had no need, and no direct incentive, to focus.

To investigate whether task irrelevant stimuli might not be consciously perceived during the main task, we ran a control experiment (which was already described in the published Study Protocol of this experiment[2]). We used the same stimuli and task structure as the main experiment, but performed a surprise memory task at the end of this control experiment. We used memory encoding as a proxy of visibility and focused on whether (old-new) recognition differed between the task relevant and task irrelevant conditions. We reasoned that memory performance would not be high overall, as participants did not expect to be tested on the stimuli, and had no reason and/or incentive to memorize them. Yet we still asked if those stimuli that were task irrelevant, and potentially might not have been perceived during the memory task, give rise to differential recognition rates compared to those that were task relevant and for whom it is reasonable to assume that they were consciously seen.

3.1 Methods

3.1.1 Participants

Thirty-nine neuro-typical participants (26 females, aged between 18 and 59, mean=32.6, SD=12.82, all right-handed) took part in the study, which was run at the Max Planck Institute (MPI) for Empirical Aesthetics in Frankfurt/Germany. All participants had normal or corrected-to-normal vision. They were recruited from the participant pool of the MPI and received monetary compensation for their participation. They all provided written informed consent prior to participation. All experimental procedures were approved by the Ethics Council of the Max Planck Society.

3.1.2 Apparatus

Stimuli (6° × 6°) were presented foveally on an LCD monitor (ASUS VG24QE, 24in., refresh rate 100 Hz, resolution 1920 × 1080 pixel) in a darkened, sound-attenuating booth. Stimulus delivery and response collection were controlled using Psychtoolbox 3[3] on Matlab 2017a, on a PC running Windows 10.

3.1.3 Stimuli and procedure

Stimuli and procedure were identical to the ones used in the main study, including the presentation of the stimuli for 3 different durations (0.5, 1.0 and 1.5 secs). The only differences were: First, 20 filler stimuli (five per category) were created for the memory test stage following

the same procedure as those used in that experiment. Second, only two blocks of 40 trials each were presented. Accordingly, 10 stimuli of each category (Faces, Objects, Letters, False-fonts) were presented in a block. Third, and most importantly, an additional session - a surprise memory test – was administered after the study (henceforth, we refer to the main study as the "exposure phase"). In the surprise memory test 40 stimuli (10 from each category) from the exposure phase (old) were presented alongside the 20 filler (novel) stimuli. Participants were asked to determine whether they had seen the stimuli in the previous exposure phase. From the 10 old stimuli belonging to a given category, half were taken from the non-targets in the task relevant condition, while the other half were taken from the task irrelevant condition. This enabled us to compare the effect of task and stimulus category on incidental memory.

The stimuli presented during the exposure phase were randomized and counterbalanced across participants such that all stimuli appeared equally often in the memory test, while also controlling for their appearance in the task condition (relevant and irrelevant), target, category, orientation and duration in the exposure phase. In each trial, a single stimulus (old/novel) was presented at fixation subtending approximately 6° by 6° of visual angle. The stimulus was shown until participants responded by pressing the left or right arrow keys on a keyboard to indicate old/new. No time-out period was implemented. Key-response attribution was randomized across participants. Participants then gave a confidence rating from 1 to 5 as to how confident they were of having seen the stimuli during the exposure phase. Participants used the 1-5 keys on a keyboard to give their response: 1 corresponds to not sure at all and 5 to being absolutely sure that they had seen the stimulus during the exposure phase. Response attribution was kept constant across participants to avoid confusion in the response mapping.

3.1.4 Analysis

Separate analyses were performed on the data of the exposure and the memory phase. Analysis on the exposure phase data was aimed at assessing participants' overall performance in the target detection tasks. For each participant, four targets were presented, one for each category. Due to the low number of target trials, we ran a Generalized Linear Mixed (GLM) model. The dependent variable had a binomial outcome, with 1 for correct detections, 0 for misses. There were therefore four data points per participant (one per category), each with a value of one or zero depending on whether participants detected a given target. The GLM model was computed with a binomial distribution and a logit link function, with stimulus category as fixed effect and participants as random effect.

To investigate visibility across task relevant and task irrelevant stimuli, we evaluated participants' performance in the memory test, defined as d', as well as the degree of confidence that participants exhibited in having seen the stimuli during the exposure phase. We computed d' defining a Hit when participants declared seeing a stimulus that was indeed presented in the exposure phase (old), and a FA when participants declared seeing a stimulus that was not presented in the exposure phase (filler). d' was computed separately per task and stimulus category and analyzed in a two-way repeated measure ANOVA with task (relevant/irrelevant) and category (faces/objects/letters/false-fonts) as within-participant factors. The median confidence per participant was computed for the different stimuli categories and task relevance separately for

correct and incorrect responses. If confidence ratings are reliable, median confidence ratings should be higher for correct decisions than for incorrect ones.

All analyses were performed in MATLAB and Statistics Toolbox (Release 2019, The MathWorks, Inc., Natick, Massachusetts, United States). The repeated measures ANOVA were computed using the fitrm function. The post-hoc multiple comparisons were performed using the multcompare command with Bonferroni correction for multiple comparison correction. Linear mixed models (LMMs) were computed using fitlme Matlab function.

3.2 Results

3.2.1 Exposure phase

Hit counts were consistently high across participants, in line with the findings of the main experiments (mean hit rate across categories=0.92) and similar across categories ($F(3,152)=0.24$; $p=0.871$; Faces: 38/39; Objects:37/39; Letters: 37/39, False fonts: 32/39) indicating that all participants complied with task instructions.

3.2.2 Memory phase

A repeated measures ANOVA on d' revealed an effect of category ($F(3,114)=11.61$, $p<0.001$), whereby overall d' for Objects was higher than for faces (diff=0.61, $p=0.003$), letters (diff=0.59, $p<0.001$) and false-fonts (diff=0.83, $p<0.001$) indicating that Objects were overall better remembered in this task. The effect of category was modulated by task relevance ($F(3,114)=3.93$, $p=0.01$). As Supplementary Figure 13 shows, objects and letters were remembered similarly across the task relevance conditions ($p>0.5$); whereas memory for faces and false-fonts was higher for the task relevant than for the task irrelevant condition (face diff=0.35, $p=0.011$; false-fonts diff=0.33, $p=0.002$). Critically, no differences between the task relevant and task irrelevant conditions were observed for stimulus categories that were overall better remembered (e.g., objects); and such differences were mostly present for those stimulus categories that were less well encoded in memory (e.g., false-fonts).

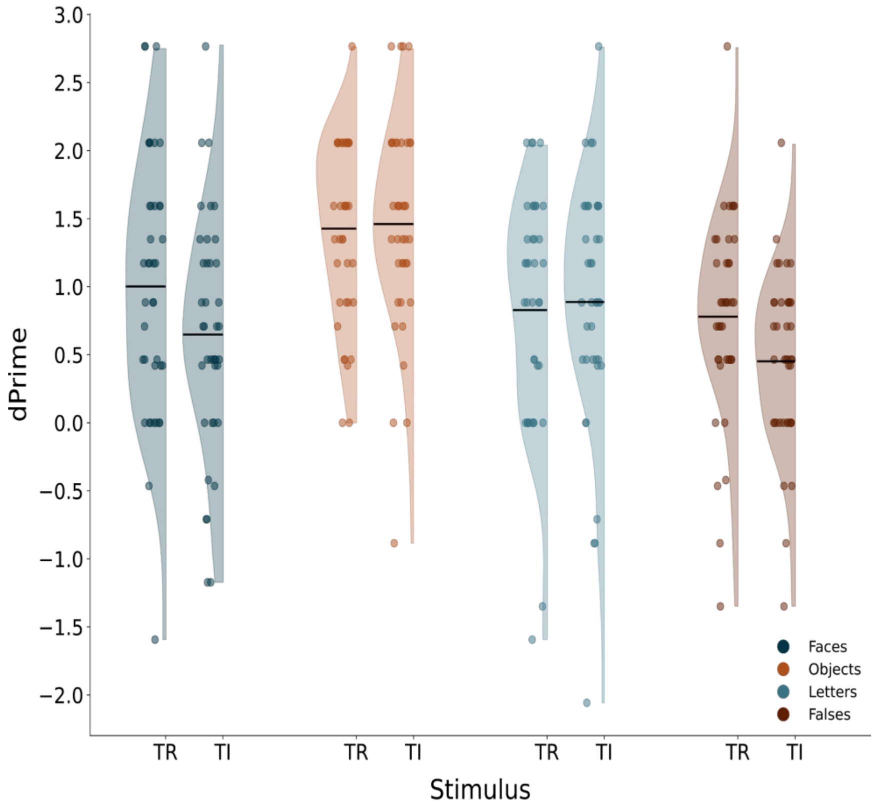

Supplementary Figure 13. d' in the surprise memory test for the different stimulus categories (faces: blue; objects: orange: letters: turquoise; false-fonts: brown) and task relevance conditions (TR: task relevant; TI: task irrelevant). Each dot is an individual participant (N=39). Black horizontal lines depict the mean for each condition. A two-way repeated measure ANOVA (two-tailed) on d' with task relevance and category as within-participant factors revealed an effect of category ($p<0.001$) and an interaction with task relevance ($p=0.010$).

Next, we investigated confidence ratings. We compared the median confidence rating in a linear mixed model with factors: category, task relevance and accuracy (i.e., whether the response was correct or not). As expected, confidence ratings were higher for correct than for incorrect responses ($F(1,533)=28.23$; $p<0.001$), validating the participants' responses in the task. Importantly, as shown in Supplementary Figure 14, confidence ratings were similar across tasks and categories. Accordingly, no main effect of task or interaction between task, and category or accuracy was found (all $p>0.05$).

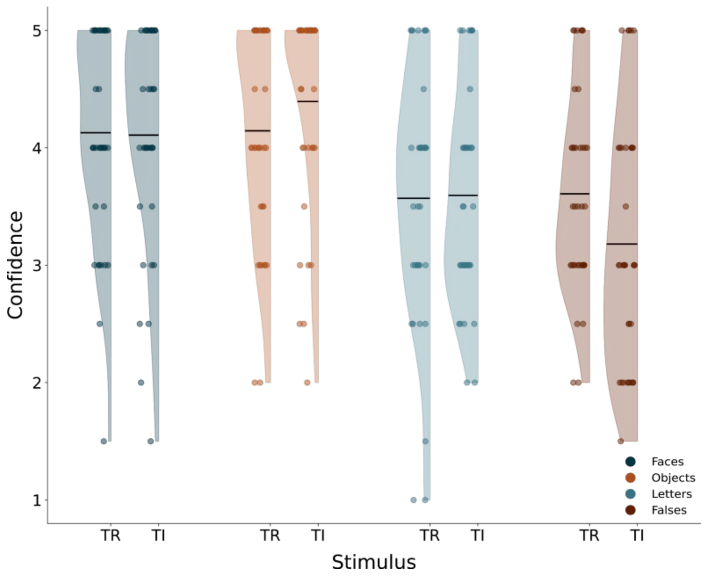

Supplementary Figure 14. Mean of the confidence rating medians to correct answers of the participants (N=39) in the surprise memory phase for task relevant (TR) and task irrelevant (TI) stimuli. Values ranged from 1 to 5 with 1 representing not being confident at all and 5 being absolutely sure of having seen the stimulus. Other plotting conventions are the same as Supplementary Figure 13. An LMM on the mean confidence by category, task relevance and accuracy showed only an effect of confidence ($p<0.001$, two-tailed).

Confidence ratings did differ across categories ($F(1,533)=15.26$; $p<0.001$), and were modulated by accuracy ($F(1,533)=4.36$; $p=0.005$). To further investigate the interaction between accuracy and category, two separate linear mixed models were run with category as a fixed factor separately for the correct and incorrect responses. Confidence ratings across all 4 categories were comparable for the incorrect responses ($F(3,247)=2.32$, $p=0.076$), whereas they differed across categories for the correct responses ($F(3,294)=21.09$, $p<0.001$). For correct responses, confidence ratings for faces and objects were similar ($t(73)=-0.84$, $p=0.0403$) but overall higher than both the confidence ratings for letters and false-fonts (all $p<0.001$), which were comparable among themselves ($t(69)=1.49$, $p=0.140$).

3.3 Conclusions

The results of this control experiment mitigate the concern that participants might not have been aware of the task irrelevant stimuli (or were aware less often than for the task relevant stimuli). When presented with a surprise memory test on the previously shown stimuli, participants' performance for task relevant and task irrelevant objects and letters did not differ. Similarly, there were no differences in confidence ratings on the memory test across task relevant vs. irrelevant stimuli. As there is no reason to think that participants might not be aware of task relevant stimuli, the overall similar performance and confidence for task irrelevant ones renders the claim that the latter might have not been consciously perceived highly unlikely.

4. Replicability of findings: Optimization vs. replication results

As explained in the main text, we divided the MEG and fMRI data into two sets, with one-third of the data (i.e., the optimization dataset; 67 participants total, with 35 fMRI and 32 MEG participants) used for development of analysis details. This allowed us to then test the replicability of the results on the remaining two-thirds of the data (i.e., the replication dataset). Below we report the results of the optimization dataset for all analysis reported in the main paper, alongside the results of the replication dataset for comparison. The results of both the optimization and replication phase were analyzed following the exact same procedures.

4.1 Behavioral analysis

Overall, the optimization phase participants showed very high hit rates (M=97.50%, SD=2.93% across all included participants), very few false alarms (M=0.59%, SD=0.38%), and reasonably fast reaction times (M=0.64s, SD=0.10). Importantly, akin to the replication data, there were no differences in performance in the optimization data, measured using d', between labs within each modality (fMRI: $F(1, 33.02)=0.33$, $p=1.000$, $BF_{01}=17.86$; MEG: $F(1, 28.49)=0.13$, $p=1.000$, $BF_{01}=18.18$). Similarly, there were no differences in reaction times between labs within the same modality (fMRI: $F(1, 32.79)=0.004$, $p=1.000$, $BF_{01}=50$; MEG: $F(1, 30.0)=2.27$, $p=1.000$, $BF_{01}=25$). With respect to false alarms, as there were too few of them, the data could not be adequately modeled.

4.2 Eye movements analysis

Overall, much like the replication data, the participants in the optimization dataset were very good at maintaining fixation (Supplementary Figure 15; for a description of saccade direction, see Supplementary Figure 16), and their ability to do so within the first 0.5 s was not modulated by task relevance (Linear Mixed Model: $F(1, 23555.5)=0.14$, $p=1.000$, $BF_{01}=250$). However, a main effect for stimulus category was not found in the optimization dataset ($F(3,232.4)=0.37$, $p=1.000$, $BF_{01}=250$), but an interaction between category and task relevance was observed ($F(3, 23573.4)=4.70$, $p=0.008$, $BF_{10}=0.491$): When task irrelevant, fixations were slightly closer to the center for letters (M=1.52, SD=1.24) than faces (M=1.63, SD=1.29, p=0.04). When task relevant, there was no significant difference between stimulus categories (p ranging between 0.194 and 1.000), like in the replication dataset.

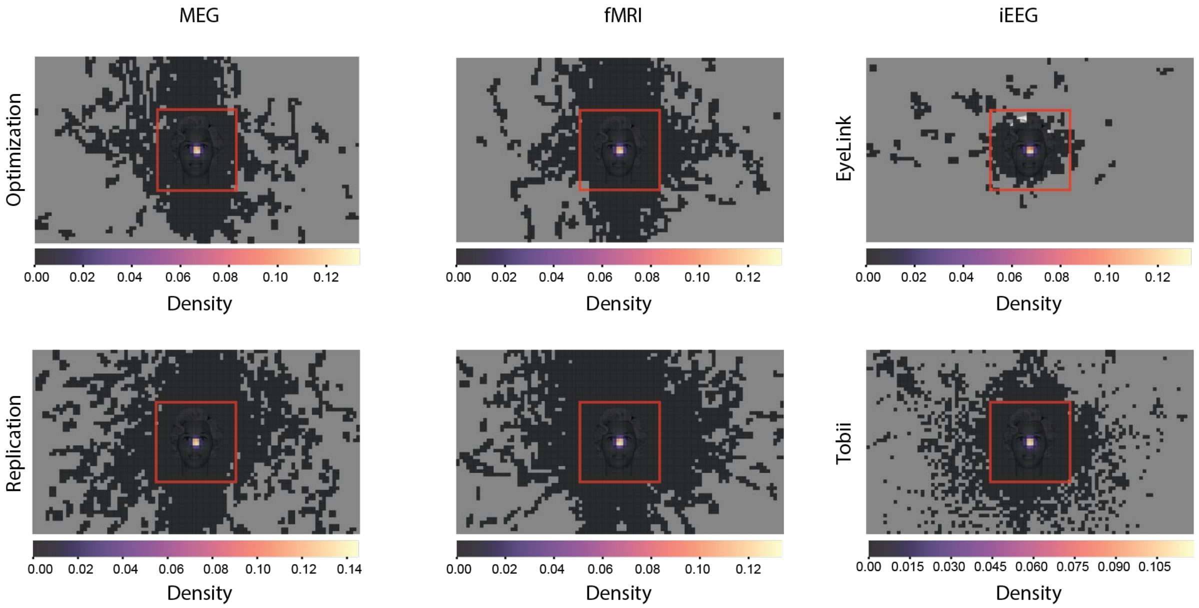

Supplementary Figure 15. Averaged heat maps of fixations throughout the experiment for the three modalities, for the optimization phase participants (upper row) and the replication phase participants (lower row), for MEG (left; optimization N=33, replication N=63) and fMRI (center; optimization N=35, replication N=71). The iEEG column presents the fixation patterns for the EyeLink sample (top; N=9) and the Tobii one (bottom; N=19), to complement Supplementary Figure 1, where only the stimulus area was presented. Face stimulus were created using FaceGen Modeler 3.1.

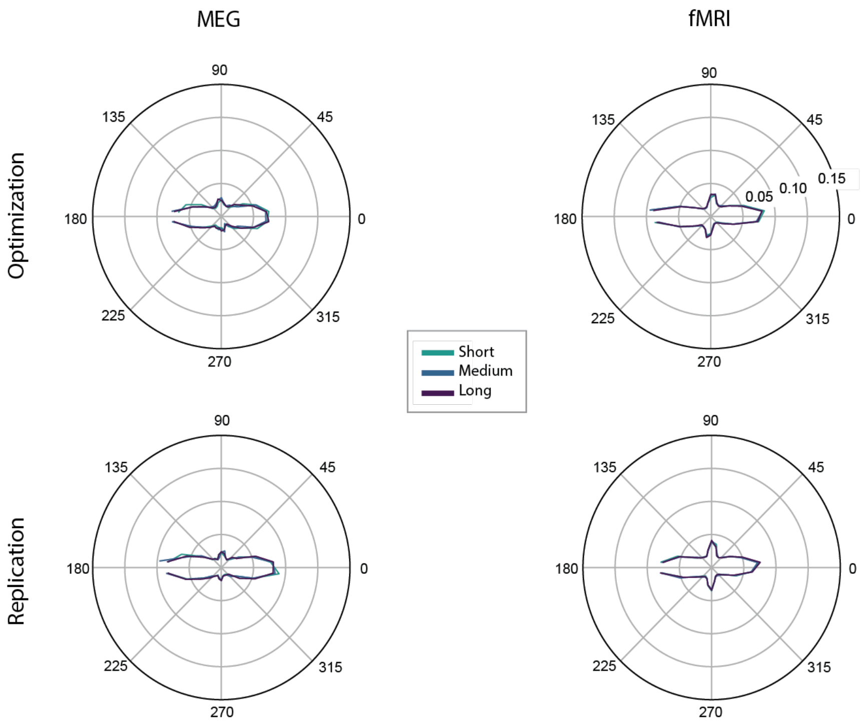

Supplementary Figure 16. Saccade direction for the optimization phase participants (upper row) and the replication phase participants (lower row), for MEG (left; optimization N=33, replication N=63) and fMRI (right; optimization N=35, replication N=71). Plotting conventions are the same as in Supplementary Figure 1.

4.3 Decoding analysis

4.3.1 MEG

Overall, the results of the optimization and replication datasets were highly consistent, with face vs. object decoding in both the posterior and prefrontal regions, which generalized from the task irrelevant to the task relevant condition and vice versa (Supplementary Figure 17).

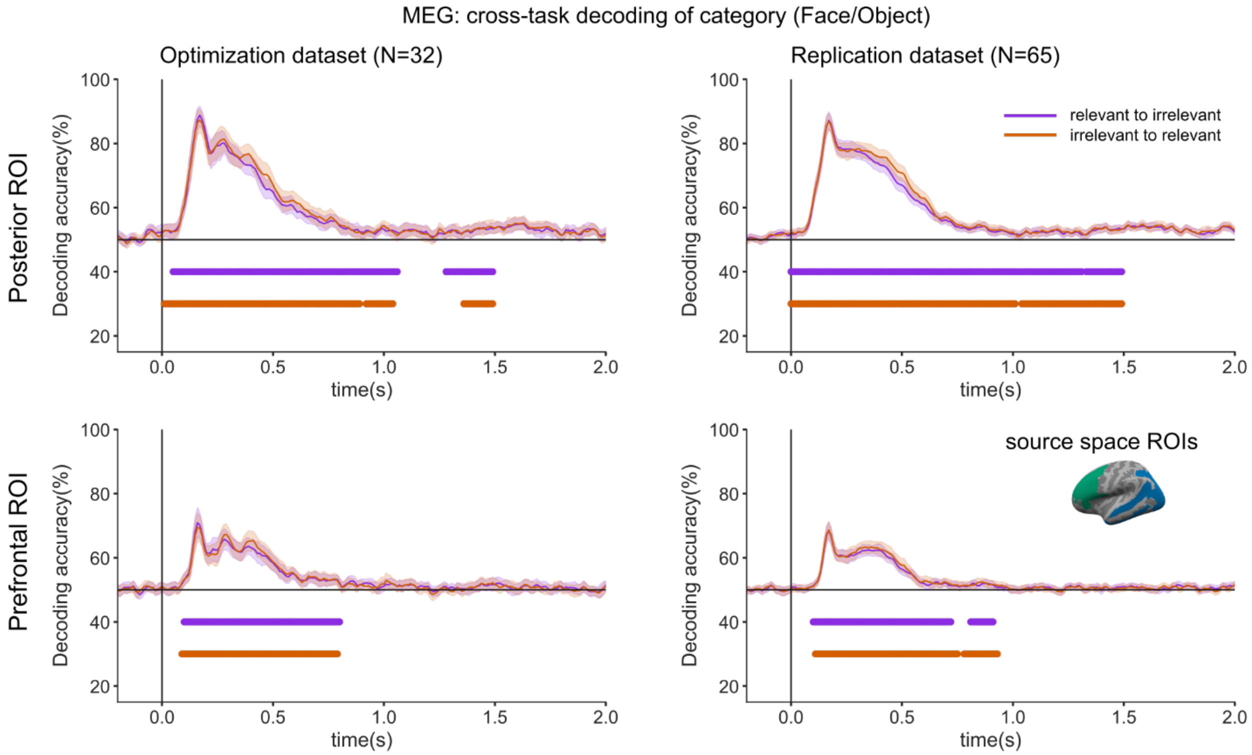

Supplementary Figure 17. Category decoding accuracy for the optimization (left, N=32) and replication (right, N=65) datasets, when training classifiers on the task relevant condition and testing on the task irrelevant condition (purple), or training on the task irrelevant condition and testing on the task relevant condition (orange), within MEG source space for posterior ROIs (top) and prefrontal ROIs (bottom). Lines under the decoding functions indicate time-points showing significantly ($p<0.05$, cluster-based permutation tests, two-sided) above chance (50%) decoding accuracies. Error bars depict 95% CIs estimated across participants. Brain surfaces in lower right panel is from Freesurfer.

Similarly, decoding of face orientation again yielded similar results for the two datasets, with strong decoding of orientation in posterior areas and very weak decoding of orientation in prefrontal ones (Supplementary Figure 18).

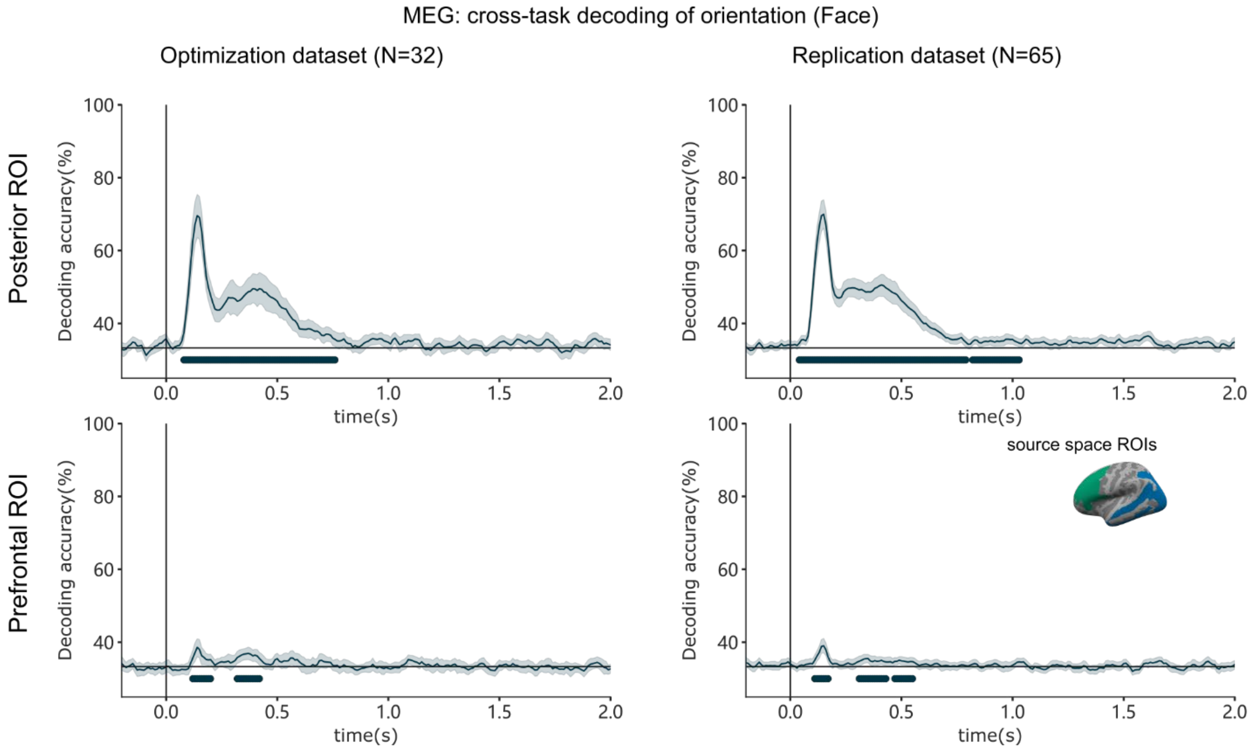

Supplementary Figure 18. Orientation decoding accuracy for faces (left vs. right vs. front view) compared across the optimization (left, N=32) and replication (right, N=65) datasets, when training and testing classifiers on the task irrelevant feature of face orientation (blue) in MEG source space for posterior ROIs (top) or prefrontal ROIs (bottom). Lines under the decoding functions indicate time-points showing significantly ($p<0.05$, cluster-based permutation tests, two-sided) above chance (33%) decoding accuracies. Error bars depict 95% CIs estimated across participants. Brain surfaces in lower right panel is from Freesurfer.

4.3.2 fMRI

Comparable results were found for the searchlight decoding between the optimization and replication datasets (Supplementary Figure 19), with significant decoding evident in similar posterior and prefrontal regions, and generalization from the task irrelevant to the task relevant conditions, and vice versa. Notably though, in the optimization results, the regions showing significant decoding were smaller in spatial extent, most likely due to the smaller sample size of the optimization dataset.

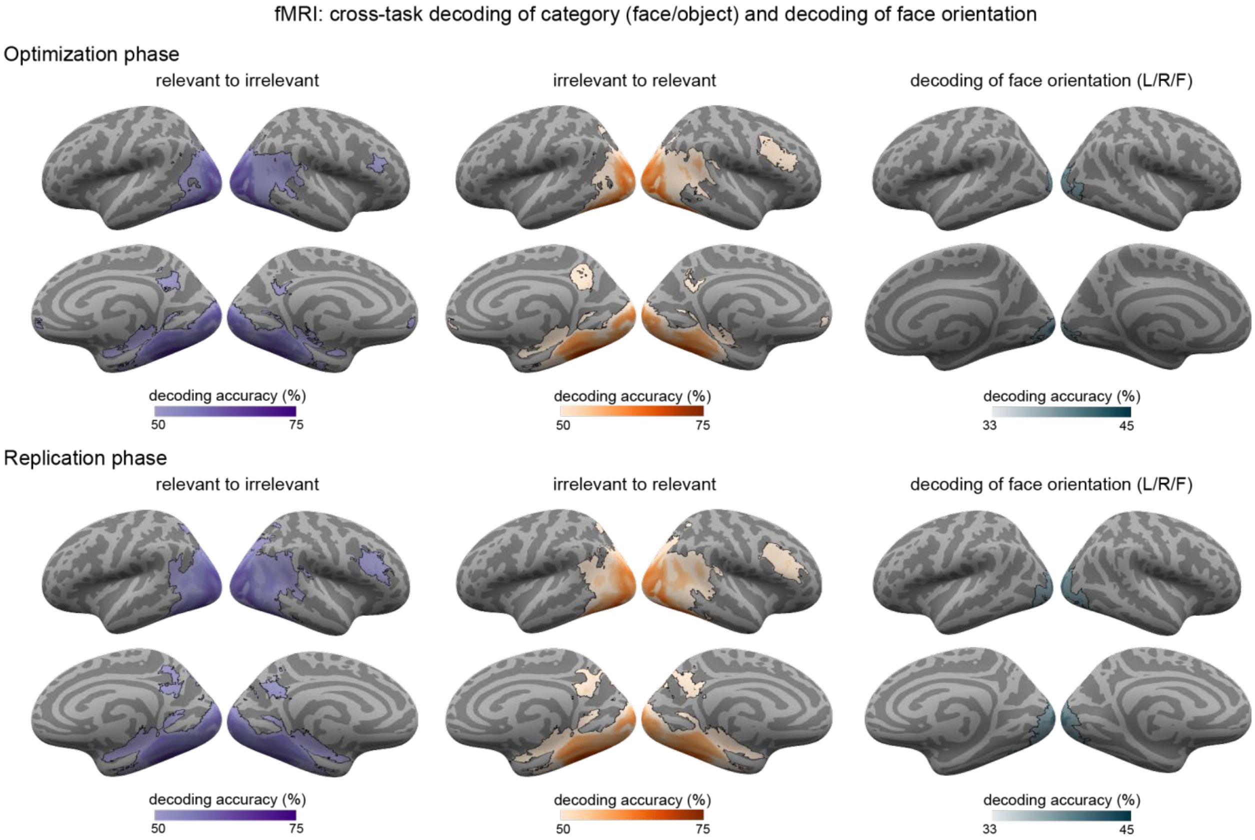

Supplementary Figure 19. Comparison between fMRI decoding results in the optimization (N=35; top) and replication (N=73; bottom) datasets. Cross-task decoding of stimulus category (faces vs. objects) when training classifiers on task relevant stimuli and testing on task irrelevant stimuli (left) or vice versa (center) and for face orientation (i.e., left vs. right vs. front view; right) which was always irrelevant, using a searchlight approach, collapsed across the three stimulus durations. Regions showing significantly (cluster-based permutation test, $p<0.05$) above-chance (50%) decoding accuracies are indicated by the outlined colored regions (left: purple; center: orange; right: green) on the inflated cortical surfaces (within each panel, top: left/right lateral views; bottom: right/left medial views). Brain surfaces are from Freesurfer.

4.4 Levels of activation analysis

4.4.1 MEG

The LMM analysis on gamma and alpha band signals in the prefrontal and posterior ROIs for the optimization dataset yielded similar results as the replication dataset (Supplementary Figure 20 and Supplementary Table 3). In both datasets, none of the models derived from the theories showed a better fit for the gamma band activity compared to the non-theoretical models in any of

the prefrontal or posterior parcels. However, the non-category-specific theories' models best fitted the alpha band activity in eleven parcels in the posterior ROI.

Altogether, the results of the analysis on the optimization data were very much in line with our main findings. In the gamma band, no support was found for either theory. In the alpha band, the late time bins provided compelling support for the IIT's model in the Occipital Pole, though not being content-specific. However, the phasic alpha response to stimulus onset and offset in the anterior and middle-posterior cingulate cortex found in the replication dataset was not found in the optimization dataset. This difference might either reflect lack of power in the optimization dataset given the lower number of participants, or suggest that this finding might be a false positive in the replication dataset.

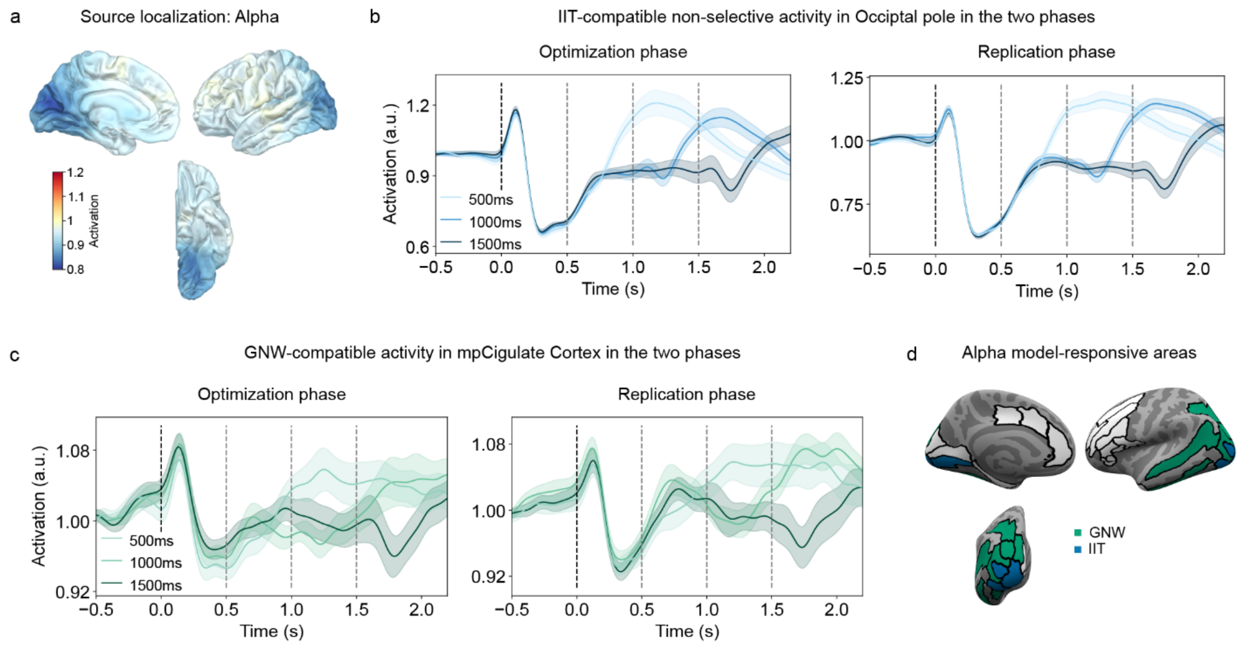

Supplementary Figure 20. Results of the MEG levels of activation analysis in the optimization (N=32) and replication datasets (N=65). **a.** Source localization of the alpha band activity in the optimization phase. **b.** Time course of the averaged alpha band activity in the occipital pole on optimization and replication datasets separately. Different shades correspond to the three different stimulus durations (light: 500ms, medium: 1000ms, dark: 1500ms) **c.** Time course of the averaged alpha band activity in the anterior cingulate cortex on optimization and replication data separately. Different shades correspond to the three different stimulus durations (light: 500ms, medium: 1000ms, dark: 1500ms). All error bars depict 95% CIs estimated across participants. **d.** Representation of the LMM results obtained during the optimization phase on alpha band activity for each parcel included in the prefrontal and posterior ROIs. Bayesian Information Criterion (BIC) was used to define the winning model. Brain surfaces in panels a and d are from Freesurfer.

ROI	# parcels	Task relevance	Signal	IIT model	IIT x Category	GNWT model	GNWT x Category
Prefrontal	12	Irrelevant	Alpha	0	0	0	0
			"Late" alpha	1 F_{IIT} = 32.78 p<0.0001	0	0	0
			Gamma	0	0	0	0
Posterior	15	Irrelevant	Alpha	4 F_{IIT} = 10.31 p<0.0014	0	7 F_{GNW} = 8.94 p<0.0029	0
			"Late" alpha	15 F_{IIT} = 8.27 p<0.0041	0	0	0
			Gamma	0	0	0	0

Supplementary Table 3. Counts of parcels for each of the fitted models of interest per ROI on the MEG optimization dataset for alpha, "late" alpha, and gamma signal separately.

4.5 Synchrony analysis

4.5.1 MEG

The results of the synchronization analysis conducted on the optimization dataset shared some, but not all of the results found in the replication dataset (Supplementary Figure 21). Namely, while a significant difference in phase-synchronization between the category-selective nodes and PFC was found within the 0-0.5 s time window in both datasets, no such difference was found between the face-selective node and V1/V2 in the optimization dataset. The DFC connectivity results showed the expected results between PFC and the face-selective node in both datasets during the ignition time window, as predicted by GNWT, while no sustained connectivity was found between category-selective areas and V1/V2 in the optimization dataset.

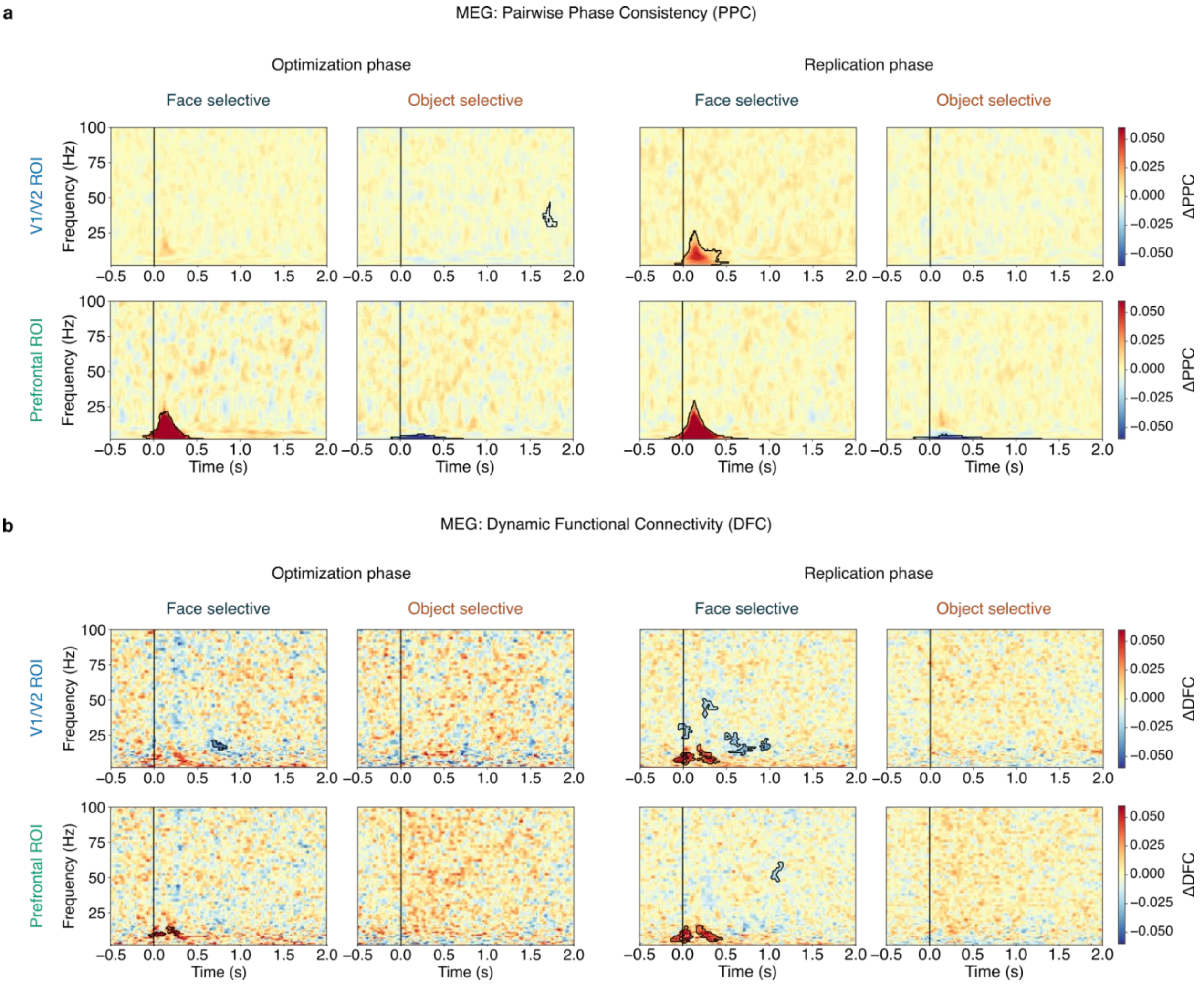

Supplementary Figure 21. Comparison of the MEG synchronization results between the optimization (N=32, left) and the replication datasets (N=65, right). **a**. PPC results were consistent between replication and optimization datasets in PFC, with significant content-selective synchrony below 25 Hz for the face-selective GED; PPC results were instead not consistent in V1/V2, with the early 25-Hz synchronization not observed in the optimization dataset. **b**. DFC results were also consistent between replication and optimization datasets in PFC, with significant early 25-Hz synchronization observed in both datasets, but not in V1/V2, where there was no early 25-Hz synchronization in the optimization dataset. Statistical significance was evaluated through cluster-based permutation tests ($p<0.05$; two-sided) in all reported analyses.

4.5.2 fMRI

Contrary to the other results which were highly similar between the two datasets, the gPPI findings from the optimization dataset did not match the results from the replication dataset (Supplementary Figure 22). As reported in the main text, the replication dataset results showed common clusters across irrelevant, relevant, and combined conditions in V1/V2, IPS, and IFG. On the other hand, the results from the optimization dataset showed scattered clusters that were not shared across the task irrelevant, relevant, and task combined conditions. Moreover, none of the clusters from the optimization dataset survived the correction for multiple comparisons, while only clusters from the combined analysis of the replication dataset survived the correction. We suggest that this discrepancy reflects the fact that the gPPI analysis requires a very large sample size[4], and accordingly yielded results only in the replication dataset, where we had twice the number of participants.

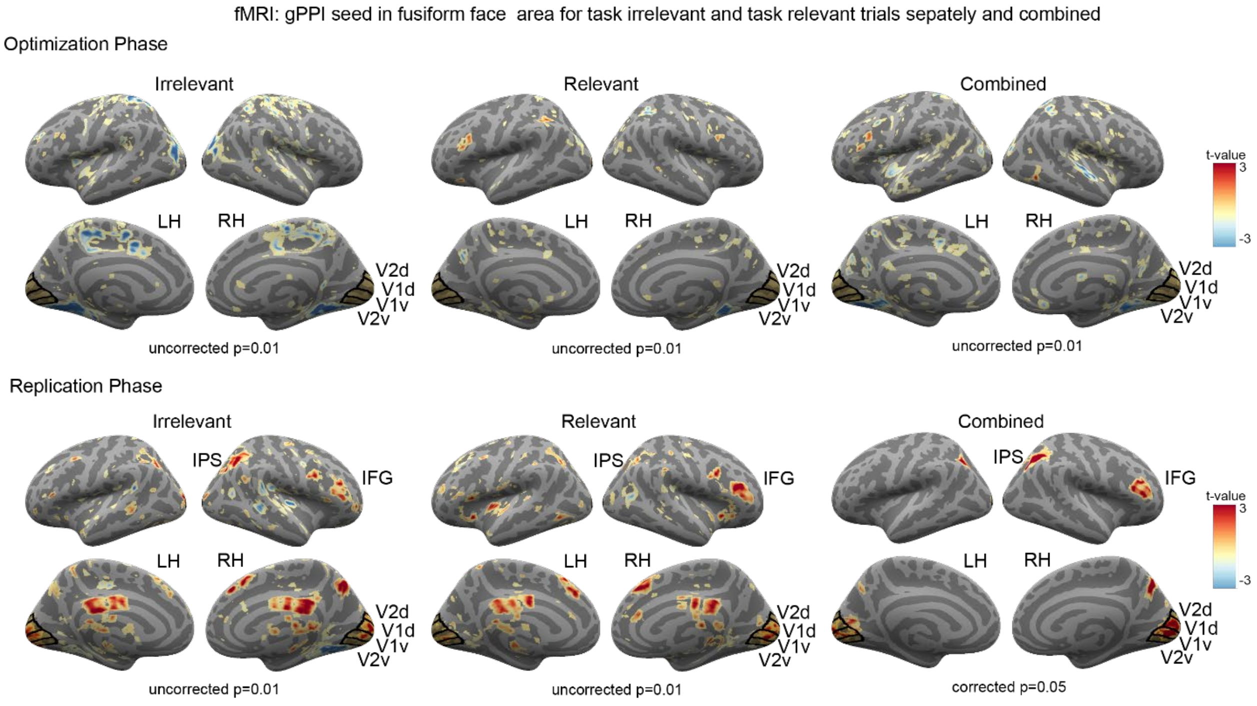

Supplementary Figure 22. Comparison between the main fMRI gPPI results with FFA as a seed in the optimization dataset (top) and the replication dataset (bottom). Results from the optimization and replication datasets were not consistent with each other, most likely due to the relatively smaller sample size (N=35) in the optimization dataset compared to the replication dataset (N=73). Combined relevant and irrelevant conditions in the replication dataset (bottom right) showed statistically significant regions (cluster-based permutation test, p<0.05, two-tailed). Brain surfaces are from Freesurfer.

4.6 Putative NCC analysis

4.6.1 fMRI

Almost all of the areas identified as putative NCCs in the optimization dataset fell within the areas which were also identified in the replication dataset (Supplementary Figure 23). Notably, in the replication data we found more extended pNCCs (e.g., Fusiform Gyrus for letters and false fonts, Inferior Frontal Gyrus pars Opercularis for all stimulus categories), presumably because of the larger sample size compared with the optimization dataset.

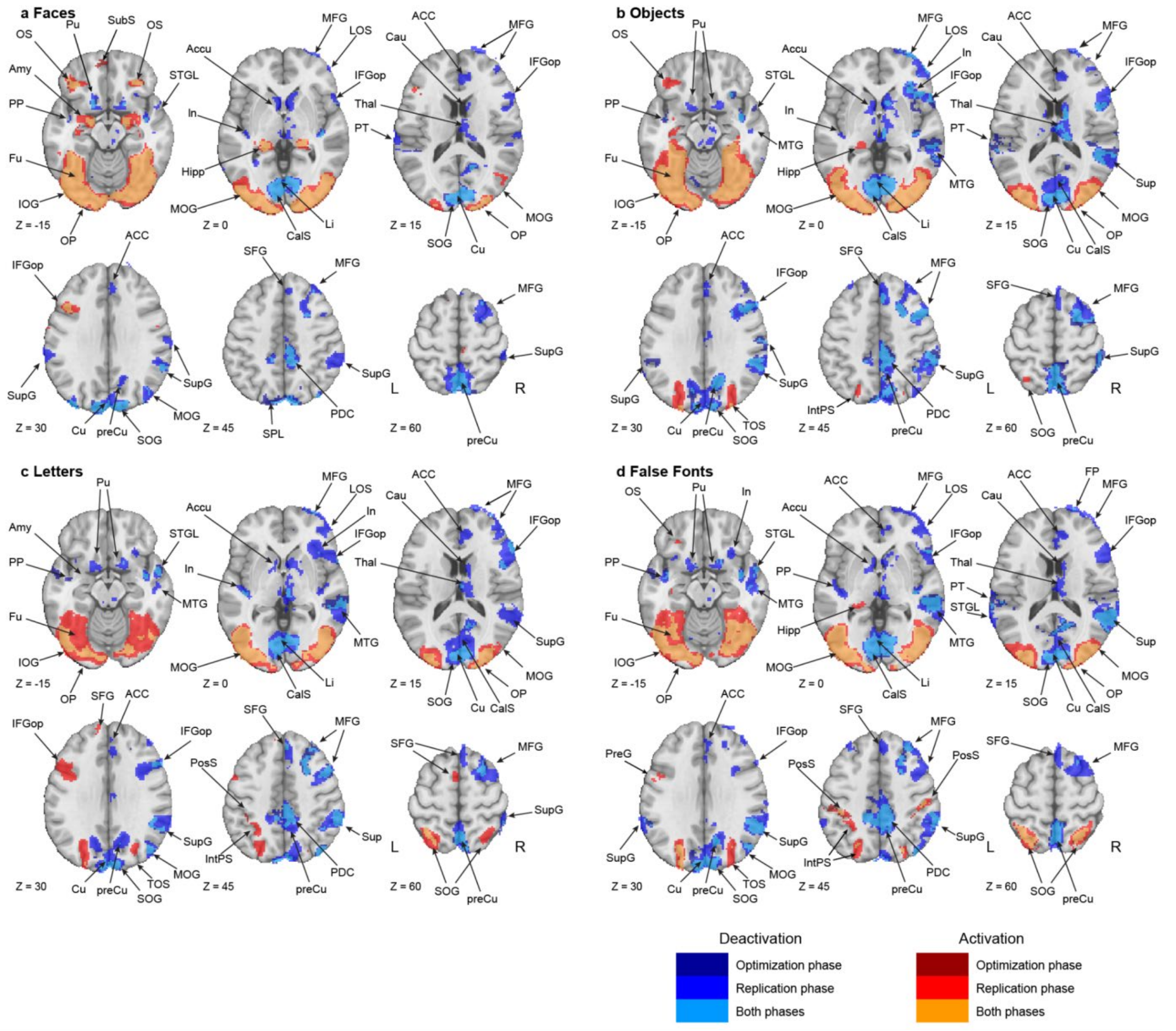

Supplementary Figure 23. Putative NCC analysis (see Methods) results for each of the stimulus categories (**a.** faces; **b.** objects; **c.** letters; **d.** false fonts), for the optimization (N=35) and replication (N=73) datasets. Color codes indicate in which of the datasets (optimization, replication, or both) results were found: Results that were found only in the optimization dataset are marked in dark red/blue for activations and deactivations, respectively, results found only in

the replication dataset are marked in intermediate red/blue, and results found in both datasets (i.e., the overlap) are marked in light blue/orange. Abbreviations: ACC: Anterior Cingulate Gyrus; Accu: Nucleus Accumbens; Amy: Amygdala; CalS: Calcarine Sulcus; Cau: Caudate Nucleus; Cu: Cuneus; Fu: Fusiform gyrus; Hipp: Hippocampus; IFGop: Opercular part of the Inferior Frontal Gyrus; In: Insula; IOG: Inferior Occipital Gyrus; Li: Lingual Gyrus; LOS: Lateral Orbital Sulcus; MFG: Middle Frontal Gyrus; MOG: Middle Occipital Gyrus; MTG: Middle Temporal Gyrus; OP: Occipital Pole; OS: Orbital Sulci; PDC: Posterior Dorsal Cingulate; PP: Planum Polare of the superior temporal gyrus; preCu: Precuneus; PreG: Precentral Gyrus; PT: Planum Temporale of the Superior Temporal Gyrus; Pu: Putamen; SFG: Superior Frontal Gyrus; SOG: Superior Occipital Gyrus; STGL: Lateral aspect of the Superior Temporal Gyrus; SupG: Supramarginal Gyrus; Thal: Thalamus; TOS: Transverse Occipital Sulcus; SubS: Suborbital Sulcus; SPL: Superior Parietal Lobule. Brain slices are from the Montreal Neurological Institute (MNI)152 template (MNI152NLin2009cAsym).

5. Prediction #1: Decoding of conscious content

5.1 Pre-registered analyses

5.1.1 Category Decoding

5.1.1.1 fMRI: Category Decoding

In the main paper, we report fMRI decoding results for face vs. objects (Figure 2 and Extended Data Table 2) and letters vs. false fonts (Extended Data Figure 1a) using a searchlight decoding approach. For completeness, we report here a table with results for the letters vs. false fonts searchlight decoding (Supplementary Table 4).

**Anatomical ROIs (Destrieux atlas)**	**Irrelevant-Relevant**		**Relevant-irrelevant**		**Irrelevant**		**Relevant**	
	n voxels	% voxels	n voxels	% voxels	n voxels	% voxels	n voxels	% voxels
**Posterior ROI**								
G_and_S_occipital_inf	1135	57	1053	52	748	37	1181	59
G_oc-temp_lat-fusifor	280	11	280	11	85	3	535	21
G_occipital_middle	902	37	889	36	379	15	1389	56
S_oc_middle_and_Lunatus	587	58	580	57	150	15	713	71
G_cuneus	293	12	277	11	139	6	285	11
G_occipital_sup	309	16	303	15	109	6	508	26
G_oc-temp_med-Lingual	347	12	340	11	223	7	228	8
G_oc-temp_med-Parahip	0	0	0	0	0	0	1	0
G_temporal_inf	362	25	329	22	133	9	638	44

Pole_occipital	984	41	959	40	580	24	1102	45
Pole_temporal	0	0	0	0	0	0	0	0
S_calcarine	124	5	131	5	55	2	121	5
S_intrapariet_and_P_trans	250	7	276	7	27	1	1368	36
S_oc_sup_and_transversal	464	33	488	34	63	4	876	62
S_temporal_sup	0	0	0	0	0	0	48	1
**PFC ROI**								
G_and_S_cingul-Mid-Post	0	0	0	0	0	0	0	0
Lat_Fis-ant-Horizont	0	0	0	0	0	0	0	0
Lat_Fis-ant-Vertical	0	0	0	0	0	0	0	0
G_and_S_cingul-Ant	0	0	0	0	0	0	0	0
G_and_S_cingul-Mid-Ant	0	0	0	0	0	0	0	0
G_front_inf-Opercular	0	0	0	0	0	0	23	1
G_front_inf-Orbital	0	0	0	0	0	0	0	0
G_front_inf-Triangul	0	0	0	0	0	0	0	0
G_front_middle	0	0	0	0	0	0	67	1
S_front_middle	0	0	0	0	0	0	0	0
S_front_sup	0	0	0	0	0	0	52	1
S_front_inf	0	0	0	0	0	0	1	0

Supplementary Table 4. Number of voxels in each ROI detected in the searchlight decoding of category (letters vs. false fonts) broken down by theory-defined ROI and for cross-task and within-task decoding.

5.1.1.2 MEG: temporal generalization of category decoding

In the main paper (Figure 2c), we presented results for the cross-task decoding of stimulus category (faces vs. objects). Below we show results of the cross-temporal generalization of decoding in posterior and prefrontal areas, both for faces vs. objects and letters vs. false fonts.

Supplementary Figure 24 shows face vs. object decoding: posterior regions exhibited decoding of category which spread across time, with regions showing decoding at both early and late time windows. Thus, decoding patterns appear to be present, disappear and are then reinstated at later times regardless of the stimulus duration. In contrast, prefrontal regions displayed only an early and transient decoding profile, predominantly along the diagonal of the cross-time decoding generalization matrix, albeit with more limited temporal generalization within this restricted time-window. While the temporal generalization patterns in PFC were consistent with those observed in the iEEG data (main Figure 2b); those within the posterior cortex were different than the iEEG data, which showed a clear pattern of duration-tracking. The source of this difference between the MEG and iEEG results in posterior regions is unclear, but may stem from the less precise spatial localization of MEG compared to iEEG, or alternatively from the different brain signal used for the two decoding analyses (High Gamma (HG) for iEEG, due to its tight relation with spiking activity, and low frequencies in the LFP for MEG). Further studies are required to better

understand the sources of these discrepancies between MEG and iEEG decoding in posterior cortex.

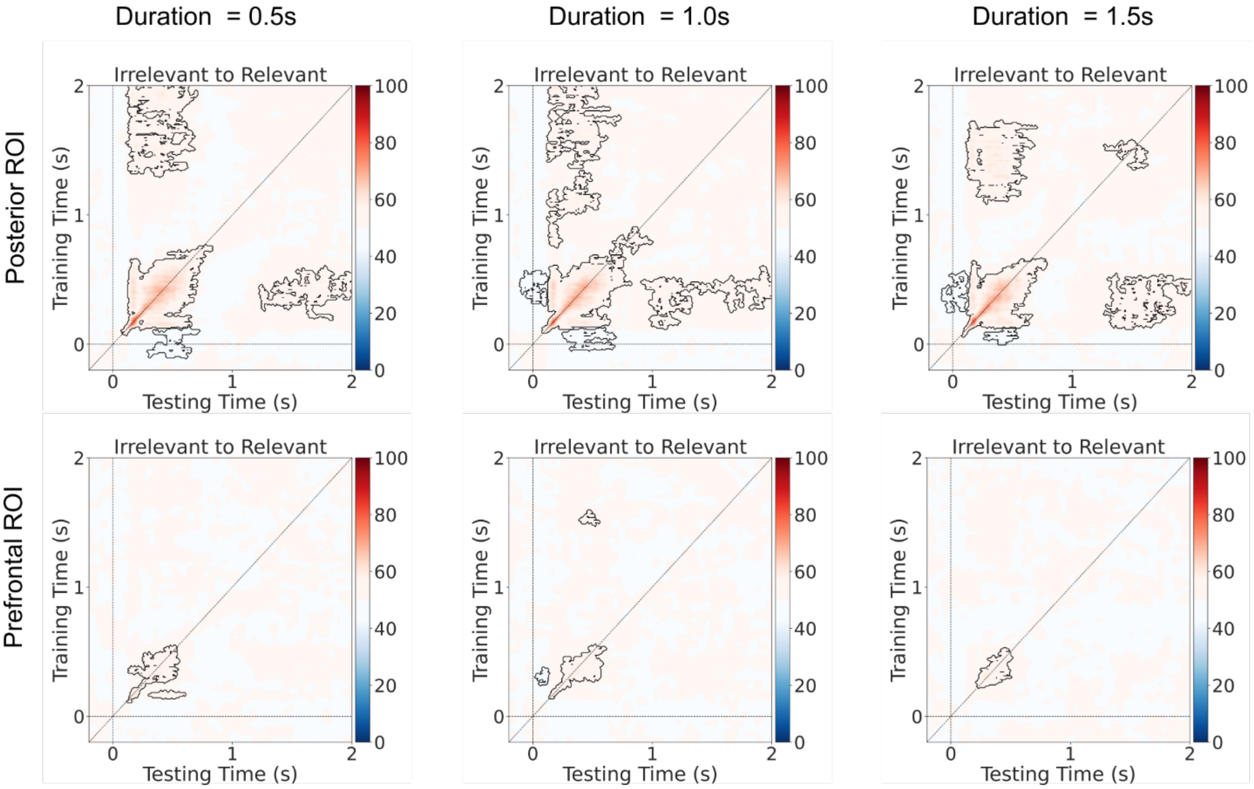

Supplementary Figure 24. Cross-time and cross-task generalization analysis for face vs. object decoding (N=65), shown separately per stimulus duration (left: 0.5 s, middle: 1.0 s, right: 1.5 s). Task irrelevant trials were used for training and task relevant trials for testing the pattern classifiers. The contour in the matrices depicts the statistically significant clusters ($p<0.05$, two-tailed) determined using cluster-based permutation. Upper row: posterior ROI. lower row: prefrontal ROI.

The same analysis was performed for decoding of letters vs. false fonts. As shown in Supplementary Figure 25, posterior regions showed a transient profile along the diagonal of the cross-time decoding generalization matrix, while prefrontal regions did not contribute to above-chance decoding of these stimulus categories when broken-down by stimulus duration (significant letter vs. false font decoding was only evident when trials across all stimulus durations were combined, as reported in Extended Data Figure 1c). These results were further confirmed through Bayesian testing with (Prefrontal ROI, Duration 500ms BF_{01}=2.58, Duration 1000ms BF_{01}=2.65, Duration 1500ms BF_{01}=2.77).

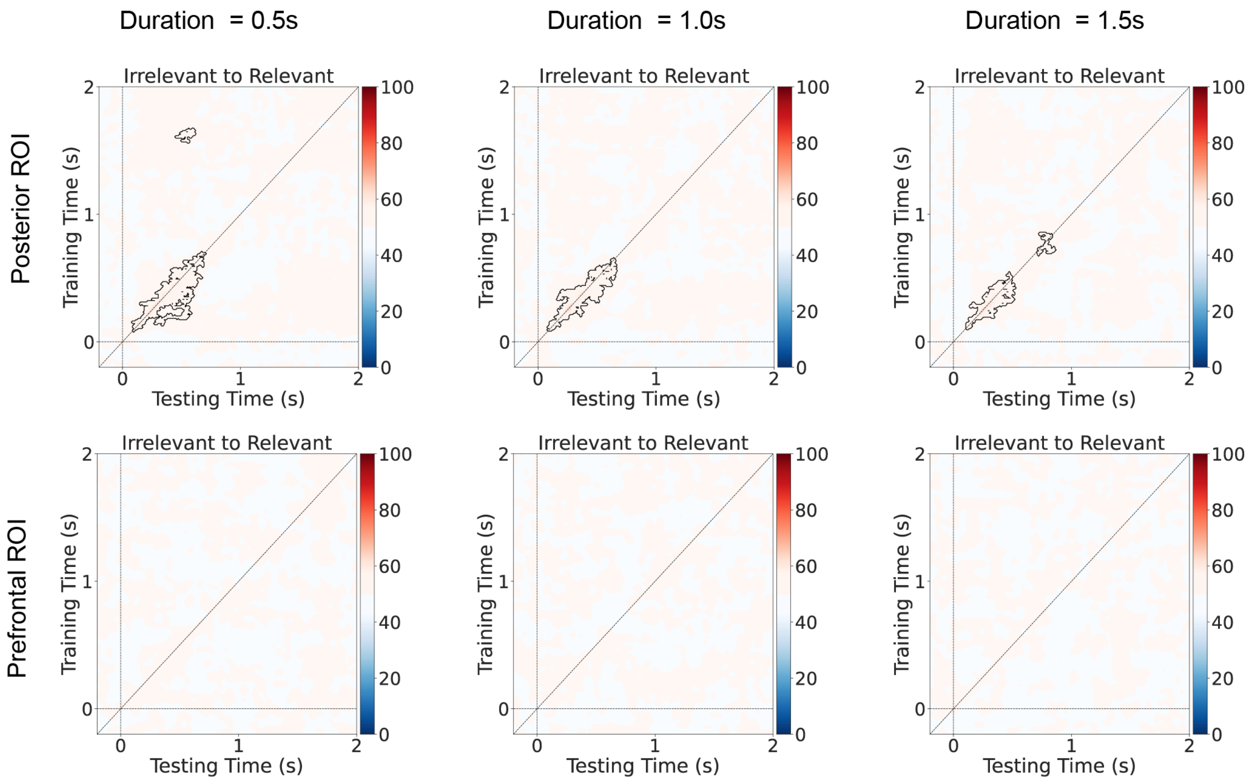

Supplementary Figure 25. Cross-time and cross-task generalization analysis for letters vs. false font decoding (N=65), shown separately per stimulus duration (left: 0.5 s, middle: 1.0 s, right: 1.5 s). Task irrelevant trials were used for training and task relevant trials for testing the pattern classifiers. The contour in the matrices depicts the statistically significant clusters ($p<0.05$, two-tailed) determined using cluster-based permutation. Upper row: posterior ROI. lower row: prefrontal ROI.

5.1.1.3 fMRI: ROI-based category decoding

In the main paper we reported fMRI decoding results with a searchlight approach. The main advantage of this approach is that results reflect an unrestricted search across the entire brain, yet they may lack sensitivity. As GNWT and IIT proponents pre-defined anatomical ROIs reflecting their predictions (see Supplementary Table 26: Anatomical Regions-of-interest (ROIs)), we implemented a decoding approach that decodes stimulus category within each of the theory-defined ROIs separately, to maximize sensitivity. This ROI-based approach further enabled us to better compare the results across modalities (e.g., with the iEEG, which were carried out on the theory-defined ROIs only).

Similar to the searchlight decoding approach, stimulus category was decoded in the task relevant and task irrelevant conditions separately. In addition, generalization of decoding from task relevant to task irrelevant conditions, and vice versa, was tested. For the task relevant/irrelevant conditions, faces vs. objects and letters vs. false fonts classification were conducted in a leave-one-run-out cross validation scheme. To test for generalization of category decoding across

conditions, faces vs. objects and letters vs. false fonts classification were done by training the classification model on one condition and testing on the other condition. Parameter estimate maps[5] of the categories of interest and a SVM classifier were employed to identify stimulus category similar to the searchlight approach.

A permutation test was used to evaluate the statistical significance of decoding within each ROI and test whether the ROIs show accuracies that significantly exceeded the chance level (>0.5). Correction for multiple comparisons across ROIs was performed using the false discovery rate (FDR) method ($p < 0.05$). Significant ROIs were identified and the corresponding average accuracy across participants at each of these ROIs were calculated and displayed on a brain surface (Supplementary Figure 26).

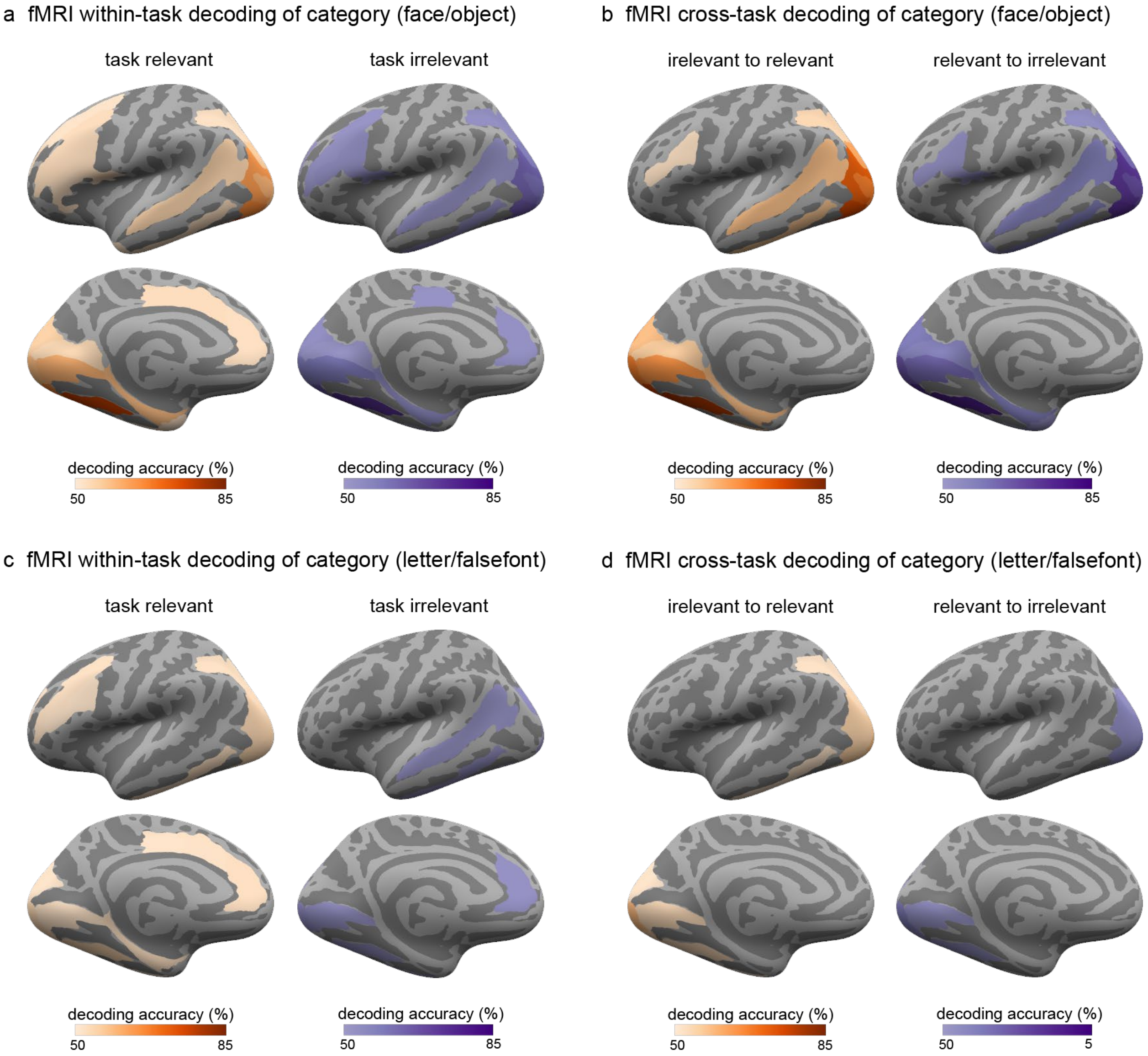

Supplementary Figure 26. Within-task and cross-task decoding of stimulus category (**a & b**: faces vs. objects; **c & d**: letters vs. false fonts) in fMRI using an ROI approach (N=73), collapsed across the three stimulus durations. Regions showing significantly (FDR correction, $p<0.05$) above-chance (50%) decoding are indicated by the outlined colored regions on the inflated cortical surfaces (top: left lateral views; bottom: left medial views). Brain surfaces are from Freesurfer.

In the posterior cortex, faces vs. objects decoding showed significant cross-task generalization in occipital, posterior temporal, and posterior parietal cortex. In the prefrontal cortex, significant cross-task decoding of category was also observed in the inferior frontal sulcus (both relevant to irrelevant and irrelevant to relevant generalization) and the opercular part of the

inferior frontal gyrus (relevant to irrelevant generalization). Significant within-task decoding was also found in both prefrontal and posterior cortices. Letters vs. false fonts decoding showed cross-task generalization and within-irrelevant decoding only in the posterior cortex while within-task decoding of task relevant stimuli showed significance in both posterior and prefrontal regions.

5.1.2 Orientation decoding

In the main paper, we reported results for orientation decoding for faces. Below we provide the full set of results for all stimulus categories (i.e., faces, objects, letters and false fonts) across all three data modalities (i.e., iEEG, MEG and fMRI). Across all techniques, we found evidence for orientation decoding in posterior areas. In PFC however, only MEG showed above chance decoding, and only for face stimuli. However, as reported in Extended Data Figure 3b, the possibility of leakage from posterior areas in MEG source space could not be ruled out.

5.1.2.1 iEEG: Orientation decoding within each stimulus category

Orientation decoding (left vs. right vs. front view faces) was performed on each stimulus category separately, both with and without pseudotrial aggregation (see Methods) for each theory ROI separately (N=29, GNWT ROIs $N_{electrodes}$=576, IIT ROIs $N_{electrodes}$=583). All task conditions were collapsed as orientation was always task irrelevant, and all stimulus durations were collapsed to increase trial numbers entered into the analysis. In posterior ROIs, stimulus orientation was decodable for all stimulus categories besides objects in an early time window (e.g., < 0.5 s) when using pseudotrial aggregation (Supplementary Figure 27). In prefrontal ROIs, stimulus orientation was not decodable for any category, with or without pseudotrial aggregation.

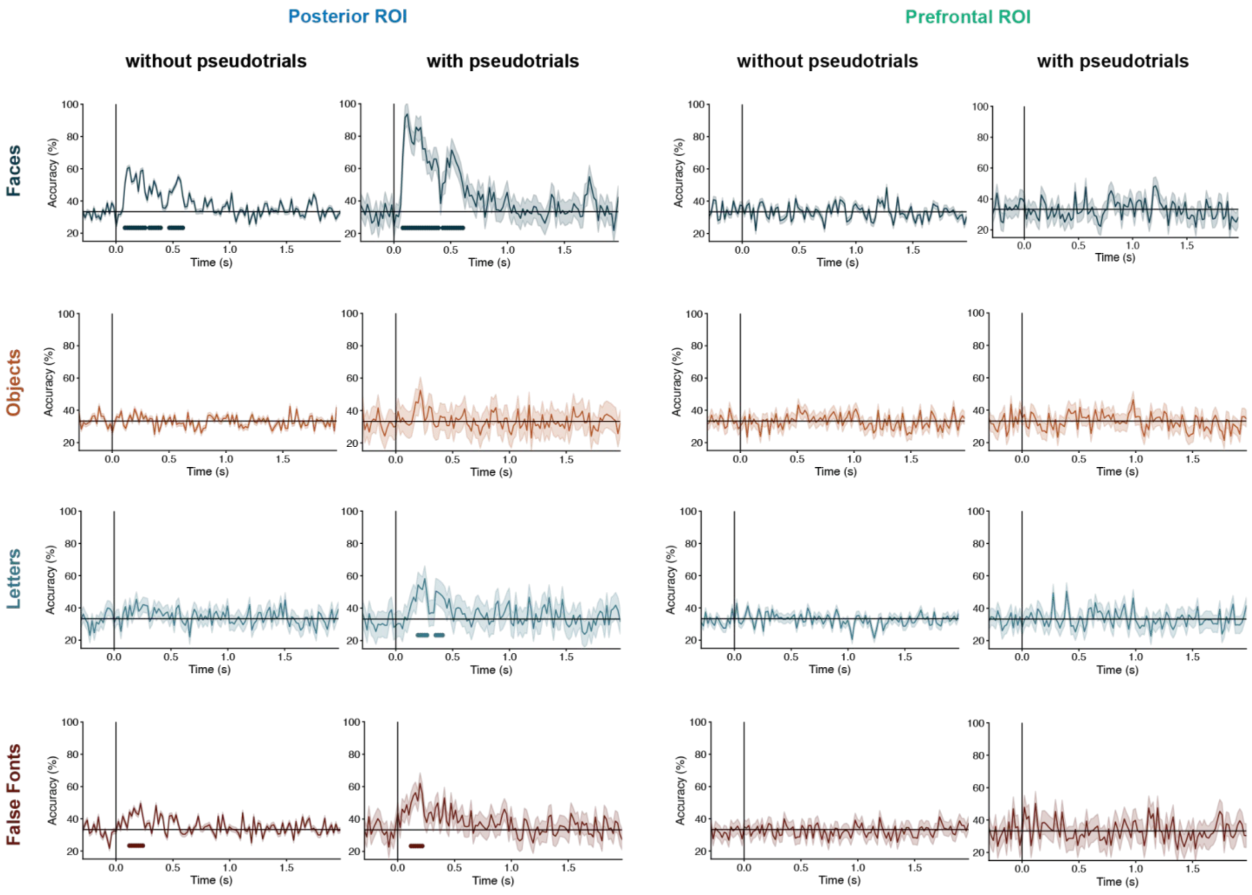

Supplementary Figure 27. Decoding of stimulus orientation (left vs. right vs. front views), which was always task irrelevant, shown for all stimulus categories within posterior ROIs (left 2-columns) and prefrontal ROIs (right 2-columns), collapsed across the three stimulus durations (N=28). Separate classifiers were trained without (left) and with (right) pseudotrial aggregation. Lines under the decoding functions indicate time-points showing significant ($p<0.05$, cluster-based permutation test, one-sided) above chance (33%) decoding accuracies. Error bars depict 95% CI.

5.1.2.2 MEG: Orientation decoding within each stimulus category

As shown in Supplementary Figure 28, in the posterior ROI we observed significant decoding of orientation (left vs. right vs. front views) for all four categories, while in the prefrontal ROI we only observed above-chance decoding of orientation for the face category(Face Orientation BF_{10}=11856.82, Object Orientation BF_{01}=2.43, Letter Orientation BF_{01}=1.98, False Font Orientation BF_{01}=2.75). Like in iEEG, all stimulus durations and tasks-conditions were combined for this analysis to increase sensitivity.

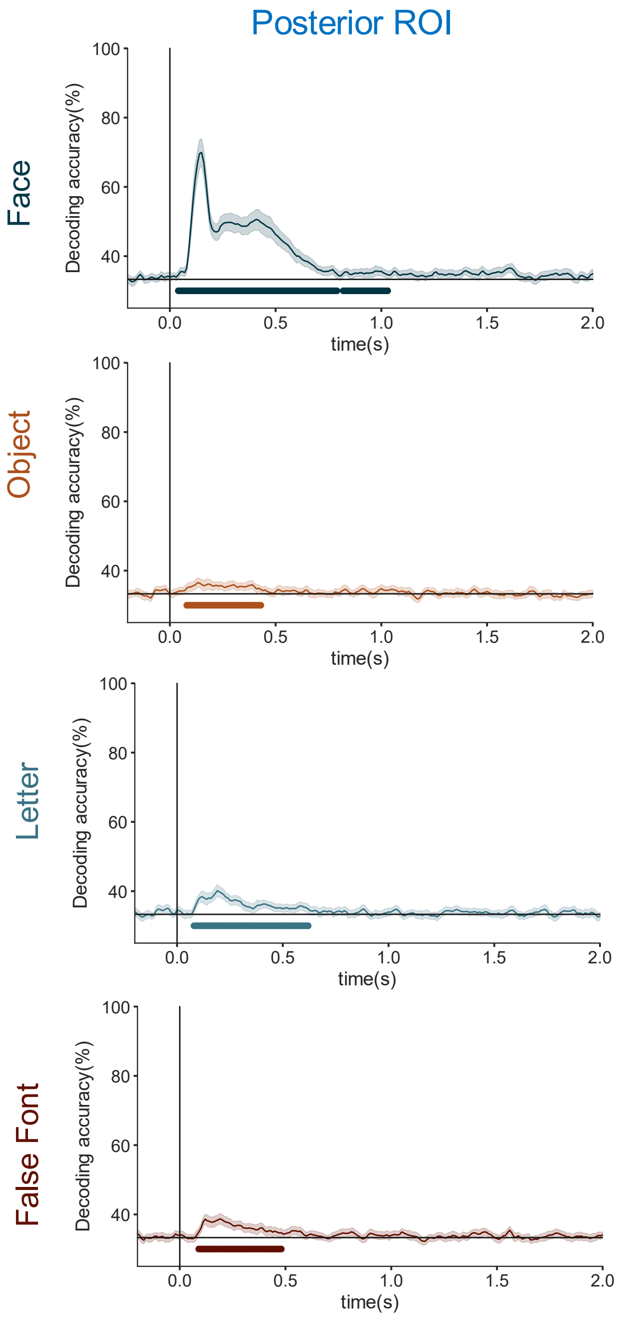

Supplementary Figure 28. Orientation decoding in MEG source space (N=65) for each stimulus category in posterior (left) and prefrontal ROIs (right). Lines under the decoding functions indicate time-points showing significantly ($p<0.05$, cluster-based permutation tests, two-sided) above chance (33%) decoding accuracies. Error bars depict 95% CIs estimated across participants.

5.1.2.3 fMRI: Searchlight decoding of orientation within each stimulus category

We tested orientation decoding (left vs. right vs. front views) for faces, objects, letters, and false fonts using the leave-one-run-out approach. The analyses were performed using the same searchlight decoding pipeline described in the main text.

Significant orientation decoding was observed in different regions of the posterior cortex for faces, letters, and false fonts while there were no significant regions anywhere in the brain showing object orientation decoding. Specifically, the occipital pole and superior occipital gyrus showed decoding accuracies significantly above-chance for orientation of faces, letters, and false fonts (Supplementary Figure 29). No prefrontal regions showed above-chance orientation decoding for any of the stimulus categories.

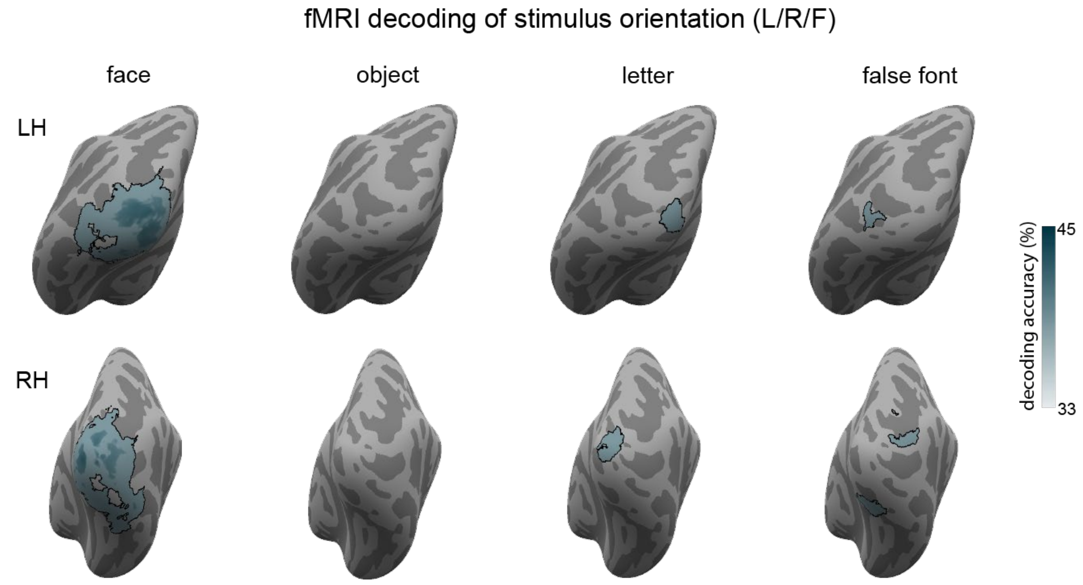

Supplementary Figure 29. Decoding of orientation (left vs. right vs. front view faces) in fMRI using the searchlight approach for faces, objects, letters, and false fonts (N=73). Regions with significantly (cluster-based permutation test, $p<0.05$, one-tailed) above-chance (33%) decoding accuracies are indicated in outlined blue on the inflated cortical surface maps (top: left posterior views; bottom: right posterior views). No regions in prefrontal cortex showed above-chance decoding of orientation for any of the stimulus categories. Brain surfaces are from Freesurfer.

5.1.3 Category and orientation decoding with and without PFC (including inferior frontal sulcus)

In the manuscript and in Extended Data Figure 3 c-f we reported the results of the decoding of category and orientation for the posterior ROIs alone vs. for the posterior ROIs with PFC ROIs, yet the latter excluded the inferior frontal sulcus. Arguably, the lack of increase in decoding we observed might have stemmed from this exclusion of a substantial portion of the PFC. To determine if this could have indeed explained the results, we repeated the analysis, this time including the entire PFC ROI defined by GNWT, including the inferior frontal sulcus. For an illustration of the ROIs used in this analysis, we refer the reader to Supplementary Figure 30a. The results remain unchanged for MEG and iEEG: there was no improvement in decoding accuracies for category or orientation when including the PFC ROIs, compared with the posterior ROIs. These results were further confirmed through Bayesian testing with (MEG Face vs Object BF_{01}=3.20, Letter vs Object BF_{01}=4.32, Face Orientation BF_{01}=2.76). If at all, in iEEG, a statistically significant decrease in decoding of category (faces/objects and letters/false fonts) was observed when including PFC ROIs. For fMRI, including PFC ROIs resulted in a 2% increase in decoding accuracy, akin to the 1% increase observed when the PFC ROIs did not include the inferior frontal sulcus (as reported in the manuscript). The fMRI results are reported for completion, though they are not included in IIT's original prediction due to the low temporal resolution of fMRI. Altogether, these control analyses demonstrate that the decoding results including PFC are robust to the selection of PFC ROIs, lending support to IIT's prediction that inclusion of PFC does not increase (and it may even decrease) decoding accuracy.

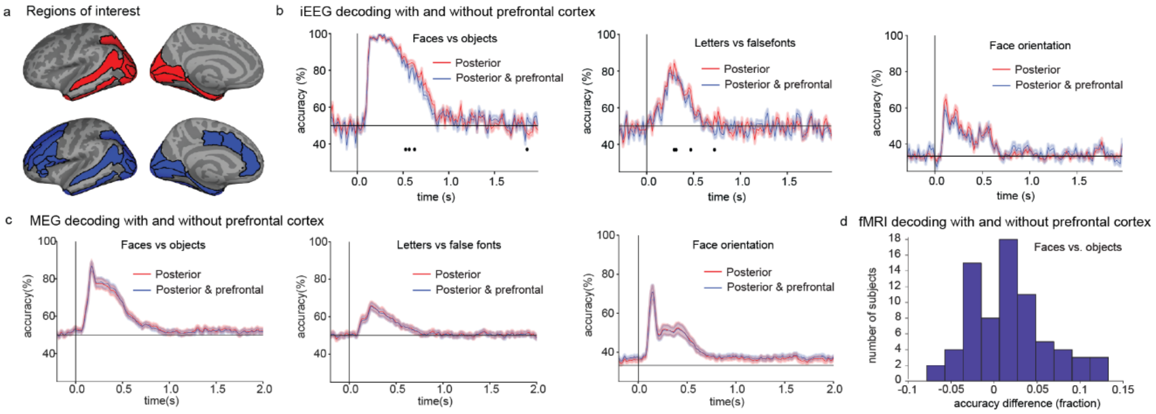

Supplementary Figure 30. Results of the decoding analysis in which the decoders of the posterior ROIs were combined with those of a PFC ROIs, including inferior frontal sulcus. The ROI selection does not affect the results reported in the manuscript: (**a**) Region of interest used in the decoding analysis including and excluding PFC areas. For iEEG (**b**) and MEG (**c**), no improvement was found, and in some cases, decoding accuracy decreased for the combined posterior and PFC ROI (blue) compared to posterior ROIs only (red), using an upper tail variance corrected paired t-

test (p<0.05). For fMRI (**d**), an advantage was found for the combined ROIs (posterior and frontal) compared to posterior ROIs only (upper tail one-sample permutation test, p<0.05), reminiscent of the original result (see further discussion in the original manuscript). In the plot, the difference in accuracy between the combined ROIs and the posterior ones is plotted on the x-axis, with the number of participants showing each value on the y-axis. iEEG N=28, MEG N=65 and fMRI N=73. All error bars depict 95% CIs. Brain surfaces in panel a are from Freesurfer.

6. Prediction #2: Maintenance of conscious content over time

6.1 Pre-registered analyses: Tracking of duration

In the main paper, we reported the results for the theories' predictions on modulations in the gamma band power (iEEG & MEG). However, the preregistered predictions specified that they could be met in either the gamma, the alpha bands, or in Event-Related Potentials/Fields (ERPs/ERFs). For completeness, we report all of these results here.

6.1.1 iEEG: Duration tracking in the different signals

In Supplementary Table 5, we describe the results for all conditions and all preregistered signals. The number of electrodes aligned with each theoretical model within each task condition, and for each type of signal (alpha, HG power and event-related potentials, ERPs) is provided. This table is complemented by Supplementary Figure 31, where these same electrodes are displayed on the brain surface, showing how they are localized in the brain.

ROI	# electrodes	Task relevance	Signal	IIT model	IIT x Category	GNWT model	GNWT x Category
PFC	655	Irrelevant	Alpha	0	0	0	0
			HGP	0	0	0	0
			ERP	2 $F_{IIT} > 24.67$ P<0.0001	0	1 $F_{GNWT} = 34.83$ P<0.0001	0
		Relevant	Alpha	0	0	0	0
			HGP	0	0	1 $F_{GNWT} = 28.80$ P < 0.0001	0
			ERP	1 $F_{IIT} = 86.49$	2 $F_{IIT \times cate} > 5.76$	3 $F_{GNWT} > 21.01$	0

				P < 0.0001	P < 0.001	P < 0.0001	
Posterior	657	Irrelevant	Alpha	1<br>F_{IIT} =10.51<br>P = 0.001	0	5<br>F_{GNWT} > 16.59<br>P < 0.0001	0
			HGP	12<br>F_{IIT} >27,26<br>P < 0.0001	13<br>$F_{IIT \times cate}$ > 7.54<br>P < 0.0001	11<br>F_{GNWT} > 20.55<br>P < 0.0001	0
			ERP	11<br>F_{IIT} =23.45<br>P < 0.0001	1<br>$F_{IIT \times cate}$ = 8.88<br>P < 0.0001	29<br>F_{GNWT} > 20.24<br>P < 0.0001	2<br>$F_{GNWT\ x\ cate}$ > 4.99<br>P < 0.001
		Relevant	Alpha	1<br>F_{IIT} =36.90<br>P < 0.0001	0	6<br>F_{GNWT} >15.29<br>P < 0.0001	0
			HGP	13<br>F_{IIT} >14.70<br>P < 0.001	22<br>$F_{IIT \times cate}$ > 3.1<br>P < 0.02	13<br>F_{GNWT} > 19.35<br>P < 0.0001	1<br>$F_{GNWT \times cate}$ = 2.10<br>P = 0.09
			ERP	16<br>F_{IIT} =27.23<br>P < 0.0001	1<br>$F_{IIT \times cate}$ = 4.16<br>P = 0.006	31<br>F_{GNWT} > 11.25<br>P < 0.0001	0

Supplementary Table 5. Number of prefrontal and posterior electrodes with a significant fit to the theories' models in the LMM analysis, separately for the alpha, HGP and ERP analysis (prefrontal ROI N patients=31, posterior ROI N patients=31).

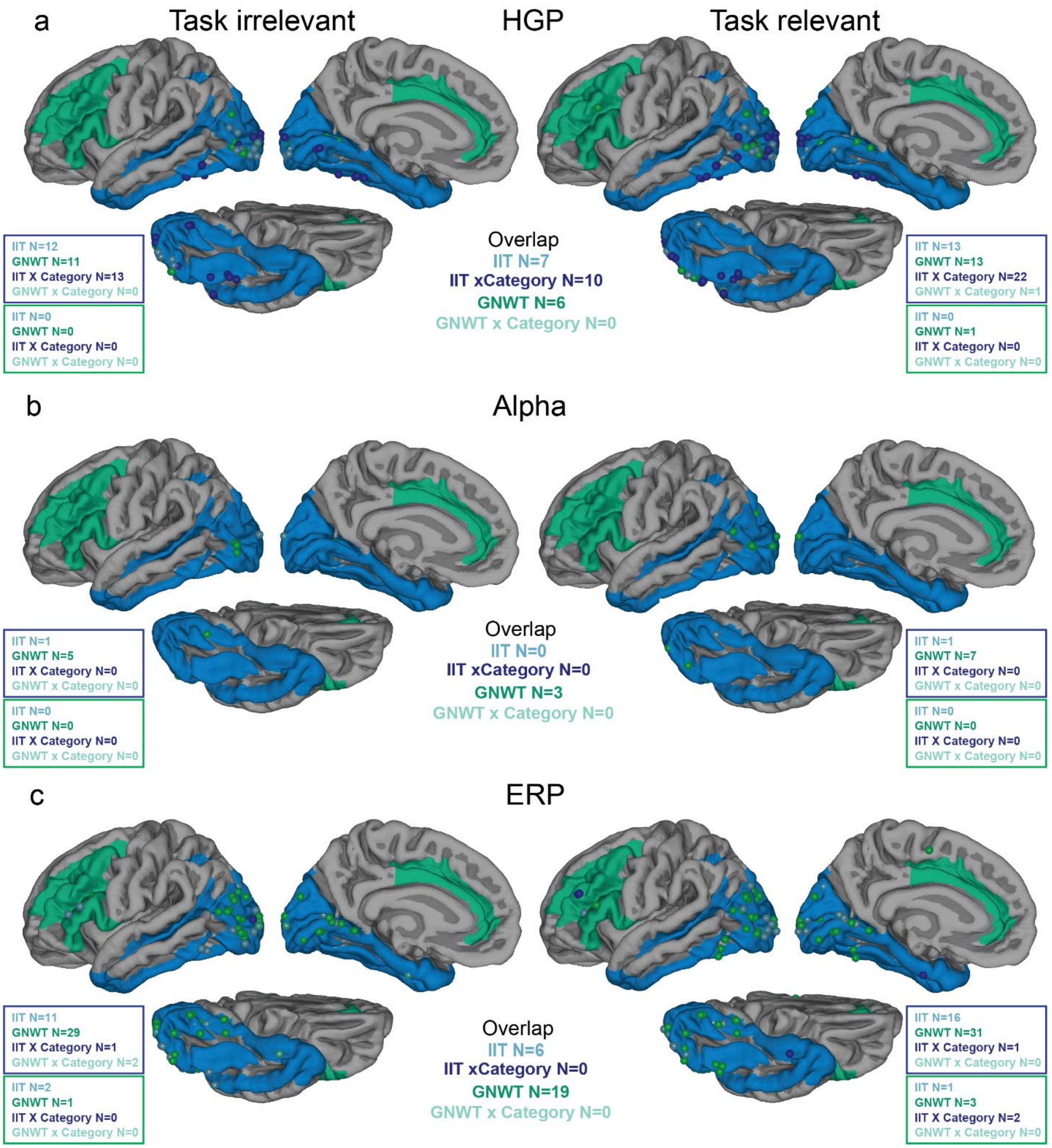

Supplementary Figure 31. Location of electrodes found to be consistent with the theories predicted activation patterns in the task irrelevant (left) and relevant (right) conditions separately in the HGP (**a**), alpha (**b**) and ERP (**c**) signals. In each case, the count of electrodes is reported for each theory ROI (blue box for posterior ROI, N patients=31, green box for prefrontal ROI, N patients=31) per theory-derived model separately for each task relevance condition, as well as the overlap (i.e., counts of electrodes for which the same model was of best fit (highest BIC) for both task relevant and irrelevant condition, labeled as overlap, and presented in the middle of the figure). Brain surfaces in panels a-c are from Freesurfer.

As both the figure and the table show, in the task irrelevant condition the results of the alpha band analysis did not provide strong support for either of the theories: none of the prefrontal electrodes showed the GNWT expected activity pattern, while in posterior sites, only one electrode showed the expected IIT pattern, and none showed the expected interaction between the IIT model and stimulus category. The ERP results, however, did provide some support for GNWT and IIT, with one prefrontal electrode whose activity was consistent with the GNWT prediction, 11 posterior electrode whose activity fitted the IIT model, and 1 electrodes showing the interaction between the IIT model and category (Supplementary Figure 32).

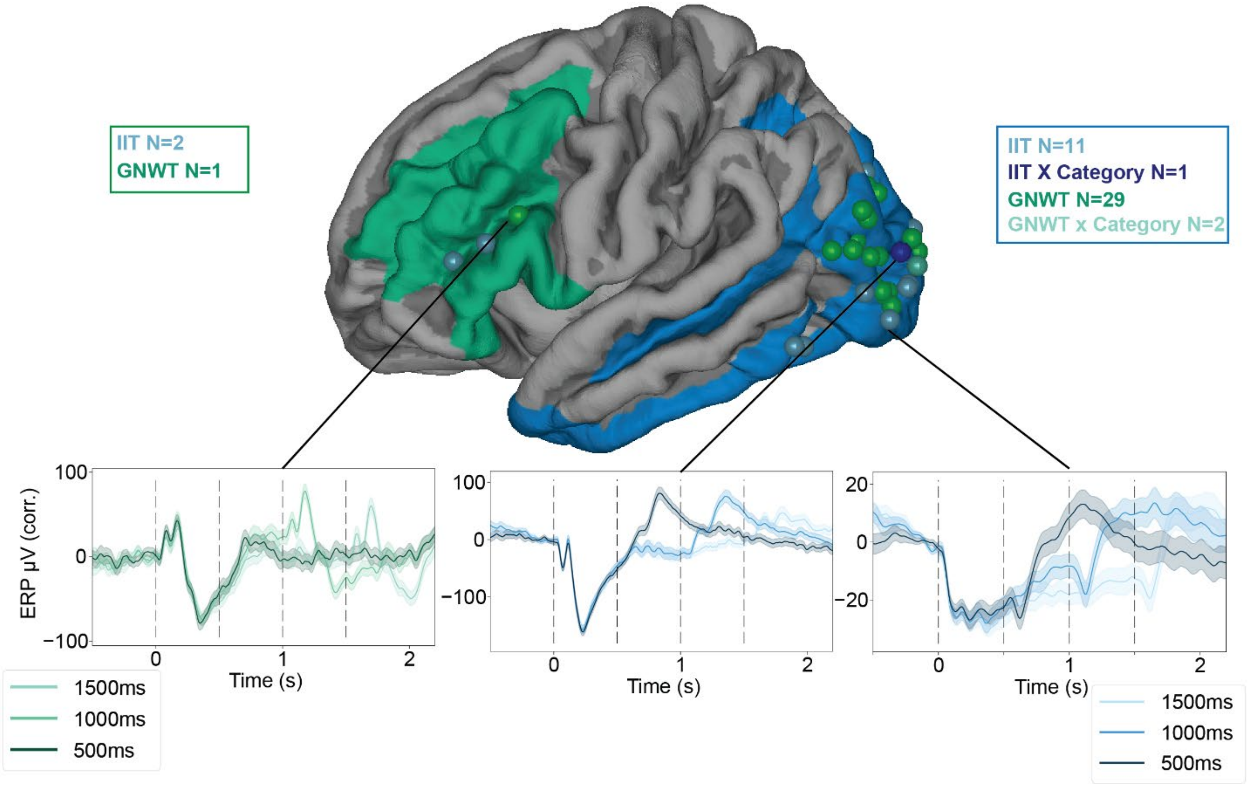

Supplementary Figure 32. Results of the LMM on the ERP signal in the task irrelevant condition. The location of the electrodes found to be consistent with the theories' models are shown on the brain surface, with the prefrontal and posterior ROIs depicted in green and blue, respectively. The time series below show the average activation across trials, separately for each duration (shaded areas represent s.e.m. across trials). On the left, an electrode found to be consistent with the GNWT prediction in the prefrontal region, in the middle is an electrode consistent with IIT prediction with category interaction, on the right is an electrode consistent with IIT without a category interaction. Response type determined by linear mixed models comparison (BIC). Error bars depict standard error of the mean. The number of electrodes is reported in the figure itself. Brain surface is from Freesurfer.

In the task relevant condition, more prefrontal electrodes (N=3) were found to be consistent with the GNWT model for the ERP signal, and one electrode fitted the GNWT model in the HGP. Notably however, these results might be driven by the task rather than by

consciousness per se (accordingly, all theory predictions were critically tested on the task irrelevant conditions).

As for consistency across task relevance conditions, as can be seen in Supplementary Figure 31 for the HGP signal, electrodes captured by the models in the task irrelevant condition largely overlapped with those captured in the task relevant condition. The only notable exception were 12 out of the 22 electrodes captured in the task relevant condition as showing a category interaction with the IIT model; these electrodes showed no interaction in the task irrelevant condition. This result can partly be explained by the somewhat weaker signal found in the task irrelevant condition, compared with the task relevant one (see onset responsiveness, category selectivity and Representational Similarity Analysis (RSA) sections), as might be indeed expected given the task manipulation. Because testing for interaction involves sub-grouping of trials, and accordingly require stronger effect sizes to be detected, it is probable that these electrodes were not captured by the model in the task irrelevant condition due to the weaker signal.

6.1.2 iEEG: Onset responsiveness and category selectivity

Electrode implantation varies considerably across epilepsy patients, as it is dictated based on medical considerations. Thus, we first aimed at characterizing the neural responses observed across the populations of electrodes in response to the stimuli presented. This analysis was done independently from the theories' predictions.

Overall, 15.9% (558 out of 3512) of the electrodes showed responses to our stimuli: out of those, 357 showed amplitude increases with respect to baseline, while 201 showed amplitude decreases. Supplementary Figure 33 shows the electrodes found to be responsive in the task relevant and irrelevant trials. Next, we characterized the latency of neural responses across brain areas. Response latencies were the shortest and least variable in occipital cortex, while latencies in other regions were found to be more variable (see Supplementary Figure 33c). We then examined the electrodes that were located within the ROIs defined by the theories. Within the posterior ROI we observed a higher number of electrodes showing amplitude increases compared to baseline than electrodes showing amplitude decreases (141 vs. 29). In the PFC ROI, the proportion of activated to deactivated electrodes was comparable (55 vs. 59).

We then established the robustness of this result using a Bayes factor t-test (Cauchy scale factor of 0.707). We observed comparable results: 440 electrodes were found to be responsive using this method, with 95.2% of those electrodes consistent with the electrodes identified using the methods reported above.

We further characterized the selectivity of the electrodes in our data set (see methods). A total of 223 electrodes were found to be selective to a given category. Faces was the category with the most selective electrodes (108), followed by objects (78), false-fonts (31) and letters (6). On average, the selectivity strength (quantified as a d') was similar across faces (M=0.88, SD=0.66), objects (M=0.70, SD=0.36) and false-fonts (M=0.71, SD=0.28), although the strongest d' values were observed for the face-selective electrodes. 89 of the 223 category selective electrodes (40.0%) were located within the posterior ROI defined by IIT (51 face selective, 24 object selective, 13 false-fonts selective and only 1 letter selective electrodes). While fewer category selective

electrodes were observed in the GNWT ROI (24/223, 10.8%), selectivity for all stimulus categories was observed (15 face selective, 6 object selective, 2 letter selective and 1 false-font selective electrodes). Most of the category selective electrodes clustered within a few ROIs (inferior frontal sulcus, 8, middle frontal gyrus, 7, triangular part of the inferior frontal gyrus, 4, opercular part of the inferior frontal gyrus, 4 on the Destrieux).

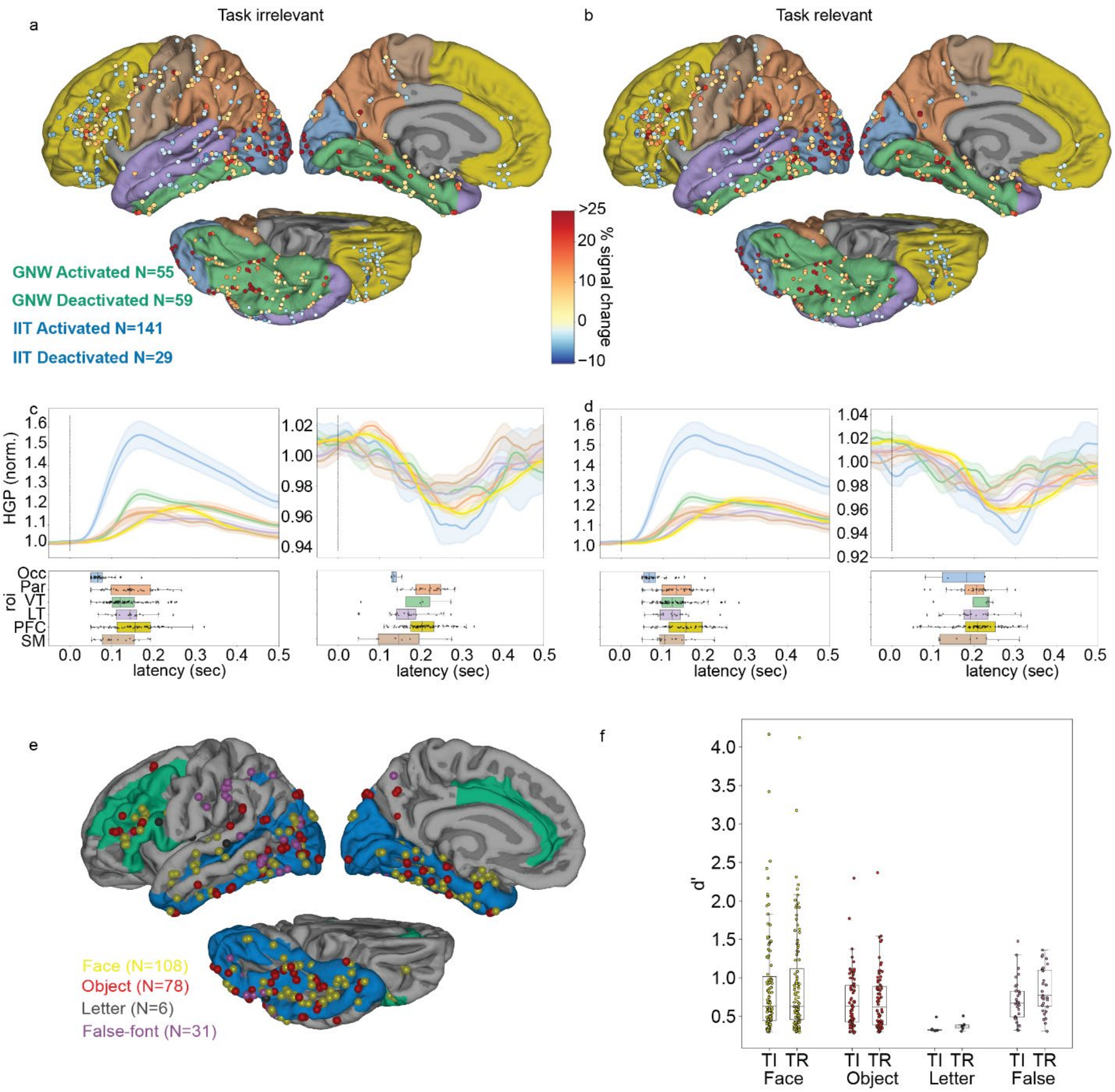

Supplementary Figure 33. Results of the onset responsive and category selectivity analysis (N patients=32). **a-b.** The location of onset responsive electrodes, color coded by the percentage of signal change between baseline (-0.3-0 s) and onset window (0.05-0.35 s) in the task irrelevant (**a**) and relevant (**b**) conditions. The color on the surfaces represents anatomical ROIs (blue: occipital

(occ), orange: parietal (Par), green: ventral temporal (VT), purple: lateral temporal (LT), yellow: PFC, brown: sensorimotor (SM)). **c.** Average HGP across electrodes in the task irrelevant condition showing activation (left) and deactivation (right, shaded area represent the s.e.m. across electrodes) separately for each ROI (colors matching the surfaces) as well as the response latencies across electrodes within ROIs in the form of boxplots. **d.** Same as panel c but for the task relevant condition. For **c** and **d**, error bars depict standard error from the mean across electrodes. Statistical significance of changes in activation was established using a two-tailed Wilcoxon signed-rank test (p<0.05, FDR corrected[6]). **e.** Location of category selective electrodes color coded per category. The color on the surfaces represent the theory ROIs. **f.** Box plots depicting the distribution of selectivity strengths (d') for each category and task relevance condition separately (each point represents the d' of an electrode selective to the corresponding category). Significance was determined using a permutation test on *d'* (p<0.05, upper tail). Brain surfaces in panels a, b and e are from Freesurfer.

6.1.3 MEG: Duration tracking in the different signals

For MEG, we used LMMs to investigate the temporal patterns of gamma band (60-90 Hz) and band alpha (8-13 Hz) power, as well as event-related fields (ERFs). Akin to the iEEG section above, here too we provide results for all conditions and all preregistered signals, within the regions of interest (ROIs) defined by the theory proponents (posterior ($N_{parcels}$=15), parietal ($N_{parcels}$=1) and prefrontal ($N_{parcels}$=11) parcels). Supplementary Table 6 details the number of parcels aligned with each theoretical model according to regions of interest, task condition, and type of signal (gamma and alpha bands as well as event-related fields, ERFs). We additionally report the results for the "Late" alpha analysis, in which we tested later time windows (200 ms later than specified in the preregistration), given the expected delay of the peak alpha signal decrease relative to the peak gamma signal increase (see below for details).

ROI	# parcels	Task relevance	Signal	IIT model	IIT x Category	GNWT model	GNWT x Category
Posterior	15	Irrelevant	Alpha	1<br>FIIT = 15.48<br>p = 0.0001	0	13<br>FGNW > 14.65<br>P < 0.0002	1<br>FGNW x cate = 0.18<br>p = 0.91
			"Late" alpha	12<br>FIIT > 30.21<br>p < 0.0001	3<br>FIIT x cate > 2.91<br>p < 0.03	0	0
			Gamma	0	0	0	0
			ERF	0	0	1<br>FGNW = 43.60<br>p < 0.0001	0

		Relevant	Alpha	2<br>FIIT > 16.60<br>p < 0.0001	0	12<br>FGNW > 10.19<br>p < 0.0014	0
			“Late” alpha	15<br>FIIT > 38.55<br>p < 0.0001	0	0	0
			Gamma	0	0	0	0
			ERF	0	0	3<br>FGNW > 19.11<br>p < 0.0001	0
Parietal	1	Irrelevant	Alpha	0	0	1<br>FGNW = 28.19<br>p < 0.0001	0
			“Late” alpha	1<br>FIIT > 23.80<br>p = 0.0001	0	0	0
			Gamma	0	0	0	0
			ERF	0	0	0	0
		Relevant	Alpha	0	0	1<br>FGNW = 20.44<br>p < 0.0001	0
			“Late” alpha	1<br>FIIT = 12.66<br>p = 0.0004	0	0	0
			Gamma	0	0	0	0
			ERF	0	0	0	0
Prefrontal	11	Irrelevant	Alpha	0	0	2<br>FGNW = 19.62<br>p < 0.0001	0
			“Late” alpha	2<br>FIIT > 22.52<br>p < 0.0001	0	0	0
			Gamma	0	0	0	0
			ERF	0	0	0	0
		Relevant	Alpha	0	0	0	0
			“Late” alpha	2<br>FIIT > 24.51	0	0	0

	$p < 0.0001$				
	Gamma	0	0	0	0
	ERF	0	0	0	0

Supplementary Table 6. Number of posterior, parietal and prefrontal parcels with a significant fit to the theories' models in the LMM analysis, separately for the alpha, "late" alpha, gamma and ERF analyses.

In the task irrelevant condition, the results provided support for GNWT, with two prefrontal parcels (Anterior and Middle-Anterior Cingulate Cortex) showing the GNWT-expected pattern of alpha band activity. However, this finding was not supported by the alpha band analysis in the late time bins. More convincing evidence was found in support of IIT, specifically in the two alpha band analyses. The observed effects were category-specific. The observed effects were specific to a particular category in three posterior parcels (Cuneus, Superior Occipital Gyrus, Superior and Transverse Occipital Sulcus), specificity predicted by IIT. The results from the gamma band and ERF analyses did not provide any support for either theory.

In the task relevant condition, neither of the theories' predictions were fully supported in the alpha band. Specifically, none of the prefrontal parcels showed the pattern of activity predicted by GNWT. At the same time, we did not observe the category-selective effect predicted by IIT in any of the posterior parcels. As in the irrelevant condition, the results from the gamma band and ERF analyses did not provide any support for either theory. More detailed information regarding the specific analyses on the alpha and ERF signals are provided below.

6.1.3.1 Gamma power

The LMM analysis on the task-irrelevant condition showed that none of the theory-based models provided a good fit to the data (Supplementary Figure 34a). We did not detect any sustained gamma band activity in posterior cortex, as predicted by IIT, neither any phasic onset and offset response in PFC, as predicted by GNW (see Supplementary Figure 34b for an example of a posterior and PFC parcel). Gamma band power was strong in posterior areas, but it is possible that the null results were due to small signal amplitudes.

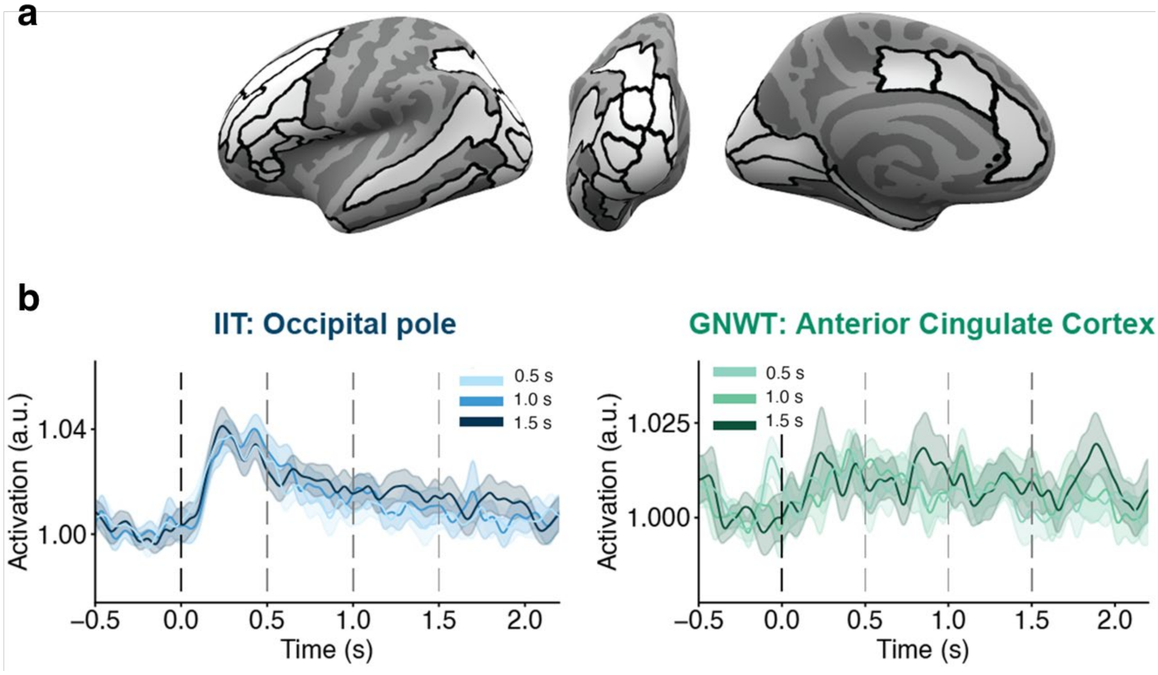

Supplementary Figure 34. Results of the LMM analysis (N=65) on MEG gamma band activity **a**. Parcels denoting ROIs showing gamma activity consistent with none of the temporal patterns predicted by the theories (white). Bayesian Information Criterion (BIC) was used to define the winning model. **b**. Left: averaged gamma activity in the occipital pole for the three stimulus durations (shades of blue), showing a pattern of activity not in line with IIT's prediction. Right: averaged gamma activity in the anterior cingulate cortex for the three stimulus durations (shades of green), showing a pattern of activity not in line with GNWT's prediction. Error bars depict 95% CIs estimated across participants. Brain surfaces in panel a are from Freesurfer.

6.1.3.2 Alpha power

Alpha band activity can be reliably detected in MEG and it is held to be anti-correlated with the gamma signal. To make sure this is indeed the case also in our data, we explored the time course of the two rhythms in the task irrelevant condition, averaged across durations. Such anti-correlation was indeed found in our data (Supplementary Figure 35), with the alpha and gamma band activities both localized in posterior areas and exhibiting an inverse correlation (i.e., while gamma activity increases, alpha activity decreases).

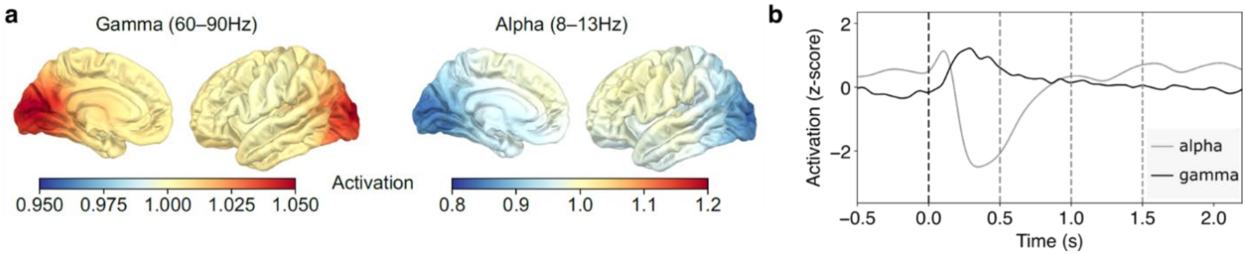

Supplementary Figure 35. a. Source localization maps for the gamma band (60-90 Hz, left) and alpha band (8-12 Hz, right) activity. **b**. Time course of the gamma and alpha band activity in occipital cortex for the task irrelevant condition (N=65). The data are averaged across all stimulus durations. The vertical axis represents the z-scored power in the two frequency bands. Gamma and alpha band activities exhibit an anti-correlated relationship. Brain surfaces in panel a are from Freesurfer.

The LMM analysis on the task-irrelevant condition showed two prefrontal parcels (Anterior and Middle-Anterior Cingulate Cortex) and a posterior parcel (Occipital Pole) matching GNWT's and IIT's predictions, respectively (Supplementary Figure 36a). In posterior cortex, alpha power decreased in a sustained manner scaling with stimulus duration (with a further phasic decrease at stimulus offset) in the occipital pole, in line with IIT predictions. In PFC, the anterior and middle-anterior cingulate cortex showed the GNWT temporal profile, with a phasic decrease in the alpha power in the 300-500 ms interval after stimulus offset (Supplementary Figure 36b). Supplementary Table 7 provides the Bayesian Information Criteria (BICs) for all tested models for each of these parcels.

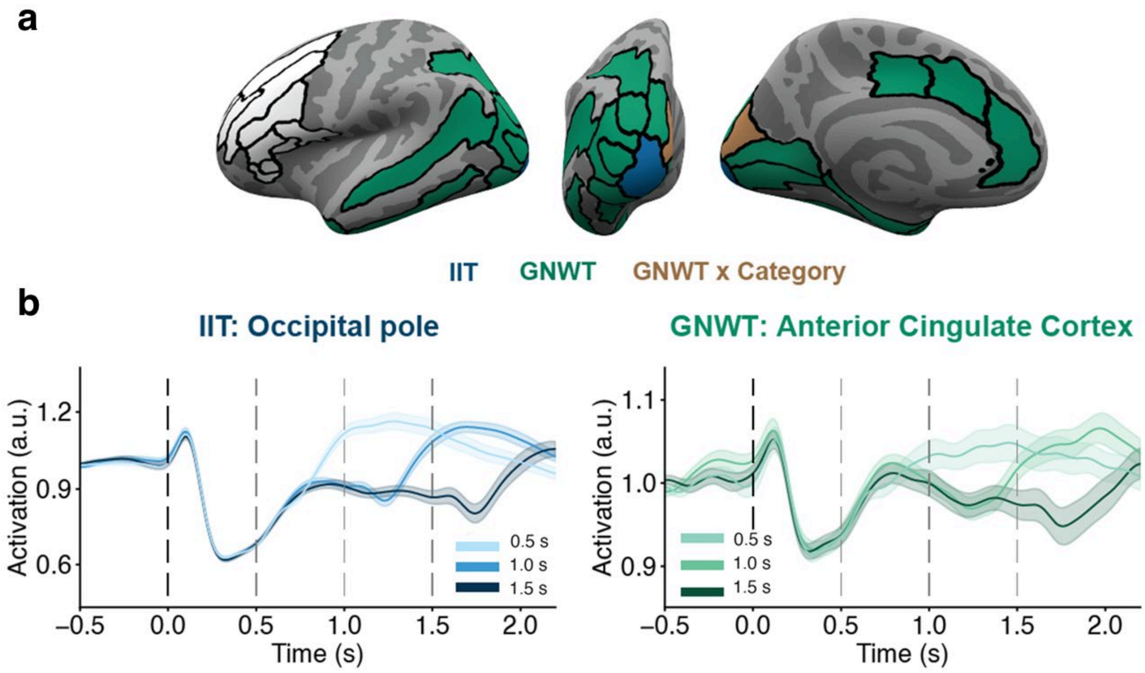

Supplementary Figure 36. Results of the LMM analysis (N=65) on MEG alpha band activity. **a**. Parcels denoting ROIs showing alpha activity consistent with the sustained duration tracking predicted by IIT (blue), the phasic onset & offset duration tracking predicted by GNWT (green), and areas showing none of the temporal patterns predicted by the theories (white). Bayesian Information Criterion (BIC) was used to define the winning model. **b**. Left: averaged alpha activity in the occipital pole for the three stimulus durations (shades of blue), best fitted by the IIT model. Right: averaged gamma activity in the anterior cingulate cortex for the three stimulus durations (shades of green), best fitted by the GNWT model. Error bars depict 95% CIs estimated across participants. Brain surfaces in panel a are from Freesurfer.

Model	Anterior Cingulate Cortex	Middle-Anterior Cingulate Cortex	Occipital Pole
Null	-2428.03*	-2307.79*	-593.34*
Time window	-2415.60*	-2297.43*	-625.27*
Duration	-2461.89*	-2336.43*	-840.26*

Time window + Duration	-2449.52*	-2326.17*	-878.06*
Time window + Duration + IIT	-2446.19*	-2324.87*	-885.72*
Time window + Duration + GNWT	-2465.26*	-2337.96*	-876.21*
Time window + Duration + IIT x Category	-2419.60*	-2302.07*	-874.86*
Time window + Duration + GNWT x Category	-2437.61*	-2308.88*	-863.20*

Supplementary Table 7. BIC values from the linear mixed model on the alpha activity for each model and parcel showing theory-predicted activity. Stars indicate that the fit of the model converged significantly (p-value < .05, two-tailed).

Given that alpha is a slow rhythm, it might be that the predicted patterns should actually be found later in time (compared to the gamma patterns for which the time windows were optimized). We accordingly conducted a control analysis using later time bins. Specifically, we analyzed the activity in the time bins 1.0 -1.2 s, 1.5-1.7 s and 2.0-2.2 s. In the posterior ROI, all the parcels showed an alpha activity in line with IIT.s prediction, with two parcels (Cuneus and Superior Occipital Gyrus) also showing category-selective responses. In contrast, none of the parcels in PFC showed the pattern of activity predicted by GNWT. While these findings confirm the sustained posterior activity previously associated with alpha, providing further support for IIT's prediction, they raise doubts about the phasic alpha band response in the PFC.

6.1.3.3 ERF signals

ERFs were calculated for each parcel in both the prefrontal and posterior ROIs (in line with the main analyses reported in the main text). The results provided no evidence for either of the theories. In the prefrontal ROIs, none of the theories' models outperformed the non-theoretical models. In the posterior ROIs, the GNWT's model best fitted the ERFs in the Inferior Occipital Cortex parcel, and the IIT's model did not outperform the other models in any of the remaining parcels (see again Supplementary Table 6 above).

6.2 Exploratory analyses: Duration predictions

Several control analyses were conducted to exclude possible confounds, to better understand the obtained results we found, or to provide the theories with the best chance possible of confirming their predictions. These control analyses are described below.

6.2.1 iEEG: Exploratory decoding analysis, with unrestricted temporal profiles and time windows

The models presented above test the theories predictions very strictly, as they only investigate activation in pre-specified time-windows. To explore if the data includes any evidence for duration tracking that is not restricted to these time windows, we complemented our planned analysis with an additional analysis that searched for these patterns using decoding along the entire signal. We accordingly trained an SVM classifier to decode stimulus duration for each electrode from 0.5 to 2 s of stimulus onset (excluding the first 0.5 s as indiscriminate of stimuli duration), using time points as features (after 0.200 s non-overlapping moving average of single trials activation). The SVM was run separately for each stimulus category (faces, objects, letters, false fonts) and task manipulation (task relevant and irrelevant). No FDR correction was applied. Electrodes were considered to track duration if the decoding accuracy was significantly above chance (label shuffle permutation test, $p<0.05$) in both task relevance conditions for at least one of the stimulus categories (see Supplementary Table 8). This approach also allowed us to investigate the robustness of the LMM in identifying the predicted patterns as well as capture any other activation patterns associated with duration. The results are depicted in Supplementary Figure 37 (alongside the results of the original LMM analysis, and the following sustained activity tracking analysis, described below.

ROI	# electrodes	Across categories	Faces	Objects	Letters	False fonts
Posterior	657	47	34	31	26	28
Prefrontal	655	4	2	3	1	1

Supplementary Table 8. Count of electrodes for which duration decoding was found, separately for prefrontal and posterior ROIs (N patients=31). Electrodes showing any duration tracking, for at least one of the categories, are reported in the column ‘Across categories’. The other columns describe the number of electrodes showing duration tracking separately for each of the categories. Notably, there was substantial overlap (i.e., most electrodes responded to more than one category).

In this exploratory analysis, we asked two questions. First, how many of the electrodes captured by the LMM analysis were also detected in this exploratory decoding analysis. Second, how many of the electrodes detected in the exploratory analysis show a pattern that matches either the IIT or the GNWT predictions (based on visual inspection of the results). As Supplementary

Figure 37 shows (panels a, b and d), the first question yielded a strong result, validating the two analyses, as substantial overlap was found between them, with all electrodes detected by the LMM approach also found in the decoding approach, except for one. As for the second question, with this more liberal approach we were only able to detect one electrode that showed an onset and offset response consistent with GNWT's predictions, yet at earlier time windows than expected (reported in the main text; see Figure 3c). The three other electrodes picked up by this analysis did not show the expected onset & offset response.

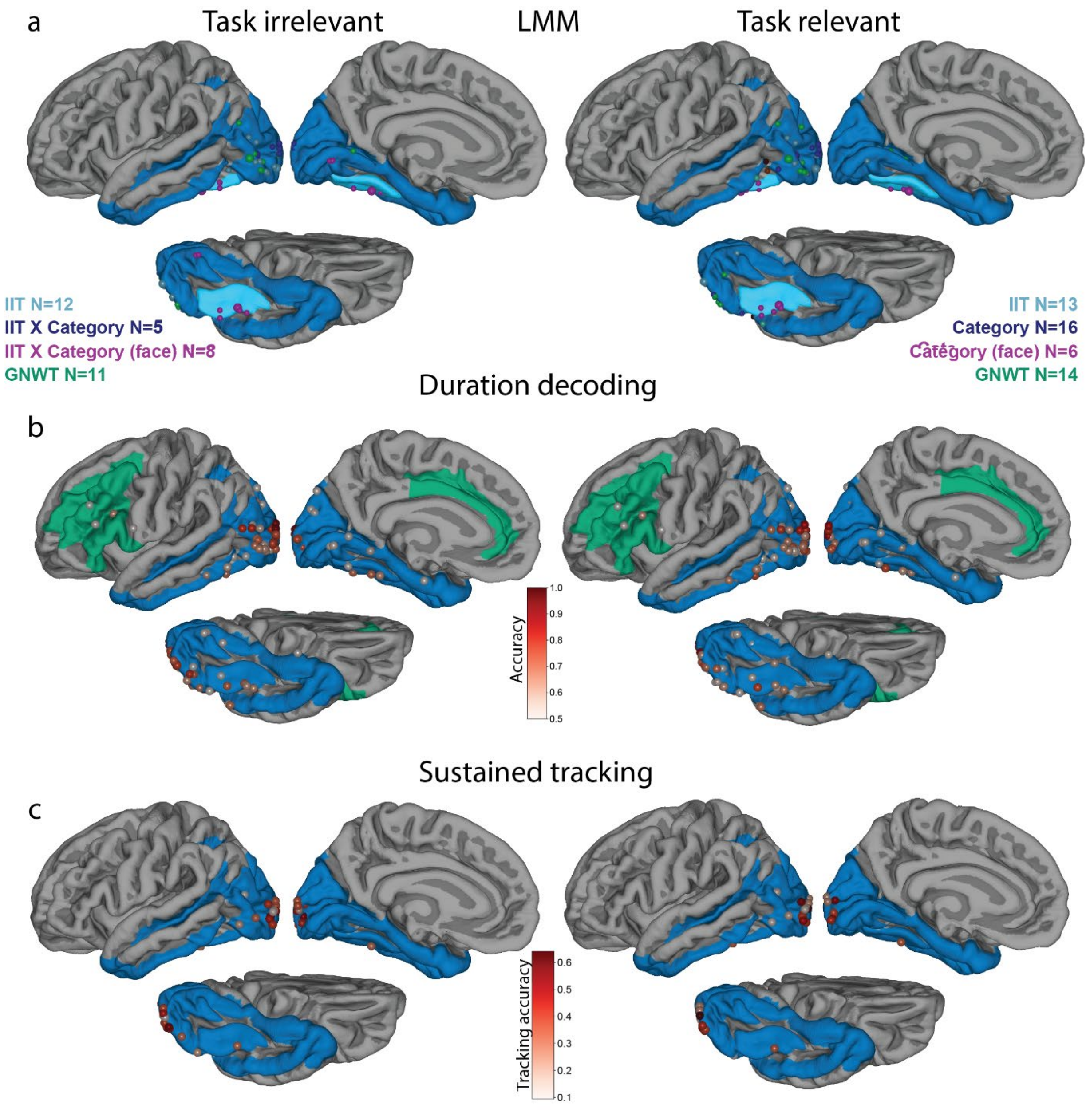

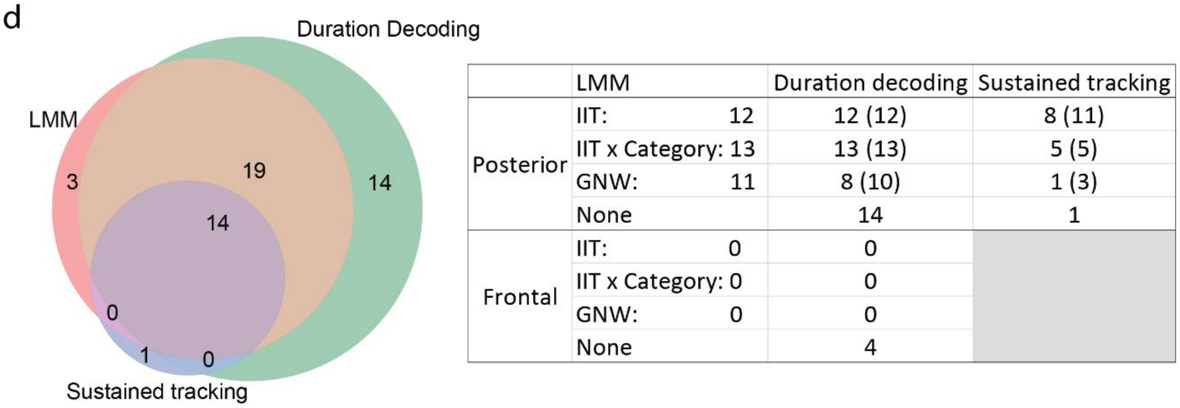

		LMM	Duration decoding	Sustained tracking
Posterior	IIT:	12	12 (12)	8 (11)
	IIT x Category:	13	13 (13)	5 (5)
	GNW:	11	8 (10)	1 (3)
	None		14	1
Frontal	IIT:	0	0	
	IIT x Category:	0	0	
	GNW:	0	0	
	None		4	

Supplementary Figure 37. Comparison of the results of the different analyses used to identify electrodes showing activation patterns associated with stimuli duration, separately for the task irrelevant (left) and relevant (right) conditions (prefrontal ROI, N patients=31; posterior ROI, N patients=31). **a.** Location of electrodes identified by the LMM models (using BIC, reported in the main text) as showing activation patterns consistent with IIT (light blue), GNWT (green), IIT with category interaction (dark blue), on which face selective electrodes showing sustained activation in a category dependent manner are marked in purple. The number next to the legend corresponds to the total number of electrodes found for each analysis. **b.** Location of electrodes with above chance duration decoding (upper tail permutation test, $p<0.05$) in at least one of the 4 stimuli categories, color coded by the highest significant accuracy observed across the 4 stimuli categories. **c.** Location of electrodes with significant sustained activation (as determined by the sustained tracking method, upper tail permutation test, $p<0.05$) for at least one of the stimulus categories, color coded by the tracking accuracy (i.e. proportion of trials for which activation was sustained for as long as the stimulus was presented on the screen, +/- 0.15 s). **d.** Overlaps across the three methods for the task irrelevant trials only. The Venn diagram (left) represents the total number captured by each method and the overlap across those. The table (right) shows the breakdown of the electrodes that were identified in the different theoretical models in the LMM analysis (left column), according to their overlap with the other methods (duration tracking, middle column; sustained activity, right column). In the latter columns, the numbers correspond to the count of LMM-detected electrodes that were also found to be significant by each method in both task conditions (alpha=0.05) while the number between parentheses corresponds to the counts of electrodes found to be significant in the task irrelevant condition (no correction). The row "none" describes the number of electrodes detected by the two analyses, which were not picked up by the LMM. Note that the sustained tracking method was not applied on the prefrontal ROIs, hence these cells are empty. Brain surfaces in panels a-c are from Freesurfer.

6.2.2 iEEG: Onset/offset analysis

The second control analysis was again driven by the lack of prefrontal electrodes showing the GNWT predicted pattern of both onset and offset ignitions. This failure could stem from three possible findings: lack of electrodes showing an onset response, lack of electrodes showing an offset response, and/or a lack of electrodes showing both an onset and an offset response. In addition, in the LMM approach, activation was averaged over broad time windows (0.2 s). If the offset responses are very short-lived in the prefrontal ROI, they might have been missed by our analysis. To disentangle between these options, we performed a time-resolved analysis. Specifically, we ran a sliding upper tail t-test across all stimuli durations from 0.3-0.5 s (against baseline from -0.2 to 0 s) following stimulus onset and offset separately. Activation was considered to be significant when a minimum of 0.02 s consecutive samples were below $p<0.05$. Only electrodes within the GNWT-defined region of interest were investigated. The results are summarized in Supplementary Table 9.

# electrodes	Significant window	Across categories	Faces	Objects	Letters	False-fonts
655	Stimulus onset	9	2	3	2	7
	Stimulus offset	0	0	0	0	0
	Both	0	0	0	0	0

Supplementary Table 9. Counts of electrodes showing increased activation following stimulus onset, offset or both in the prefrontal ROI (N patients=31). This analysis was performed for each task and category separately. The reported counts in the across categories column refer to the electrodes with activation significantly above baseline in both task conditions within at least one of the stimulus categories. The reported counts in the faces, objects, letters and false-fonts column refer to the counts separately for each category. With this analysis, we identified nine electrodes that showed an onset response between 0.3 and 0.5 s, while no electrode showed either an offset response alone (at the respective time following stimulus offset), or both an onset and offset response.

This result demonstrates first that our analyses are sensitive enough to capture the relevant responses when they occur, and second that onset responses are indeed found in PFC ROIs within the predicted time window (though in a relatively small subset of the electrodes). This pattern of results suggests that the reason the main preregistered analyses failed to support the GNWT model was the lack of offset responses.

6.2.3 iEEG: Sustained duration tracking

Finally, the third control analysis was aimed at testing for sustained activity in the electrodes identified by the modeling approach as matching the patterns predicted by IIT. Notably, the preregistered modeling approach was designed to maximize discriminability between both theories by investigating only specific time windows following stimulus onset. Thus, it is possible that the identified electrodes do not show sustained activation, but rather increased activation only in the investigated time windows.

To control for this possibility, we applied the method developed by Gerber and colleagues[7]. This method is aimed at determining how long a stimulus was presented in single trials by isolating the time point at which activation drops below a threshold defined as the median between the average baseline (-0.5 to -0.2 s) activation and post-stimulus activation (1.2 to 1.5 s) of the longest stimuli (1.5 s) only. Activation in a given trial was considered to accurately track duration if the predicted duration was within +/- 0.15 s of stimulus offset. For a given electrode, the proportion of accurately predicted trial duration was computed and compared to a null distribution of duration tracking accuracy obtained by durations label shuffles. This analysis was performed separately for each task condition and stimulus category on the 1.0 and 1.5 s trials.

Electrodes were considered to track duration if duration was correctly identified in both task conditions, for at least one of the stimuli categories. This method was applied to electrodes located in the IIT defined region of interest using the HG signal, and the results are summarized in Supplementary Table 10. Using this method, we found 15 electrodes showing sustained activity, out of the 25 electrodes found using the modeling approach.

# electrodes	Across categories	Faces	Objects	Letters	False fonts
657	15	10	10	9	5

Supplementary Table 10. Counts of electrodes showing duration tracking in the posterior ROI (N patients=31). We performed the analyses for each task and category separately. The reported counts in the across categories column refer to the electrodes in which duration was identified significantly above chance in both task conditions within at least one of the categories. The reported counts in the faces, objects, letters and false-fonts column refer to the counts separately for each category.

6.2.4 MEG: Gamma power in unified ROIs

As described previously, for the MEG data, we did not find any meaningful results with respect to the theories' predictions in the gamma range when considering the individual parcels within the prefrontal and posterior ROIs. We further tested if the predictions are borne out by the data when inspecting the entire posterior/prefrontal ROIs without dividing them into parcels. Here, we did find an early increase in gamma power with the onset of the visual stimuli in the posterior ROI, but not in the prefrontal one (Supplementary Figure 38). However, this posterior gamma activity was not sustained with respect to the duration of the stimuli, contrary to the prediction by IIT. These observations were confirmed by the results of the LMM analysis, which did not find support for the IIT models in the posterior ROI, nor for the GNWT models in the prefrontal ROI (Supplementary Table 11).

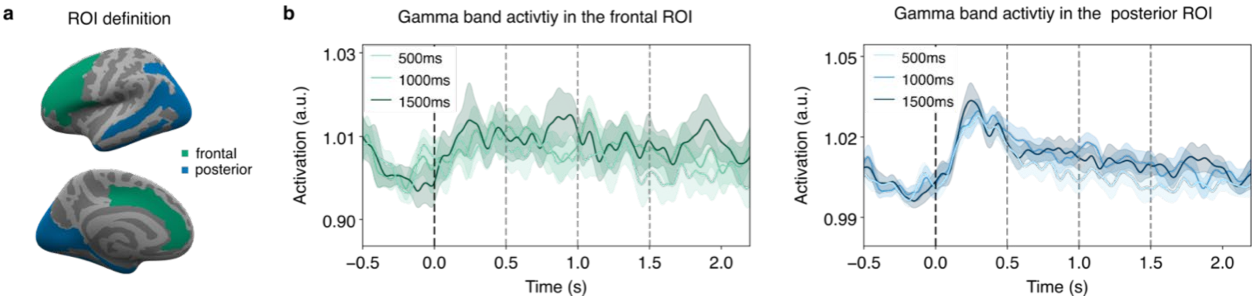

Supplementary Figure 38. Results of the LMM analysis (N=65) on gamma band activity in the entire prefrontal (left) and posterior (right) ROIs. Bayesian Information Criterion (BIC) was used to define the winning model. **a.** Brain surface plot showing the definition of the entire prefrontal and posterior ROIs in lateral (top) and medial (bottom) views. **b.** Time course of the gamma band activity in the prefrontal (left, green) and posterior (right, blue) ROIs for each stimulus duration, averaged across participants. The results did not provide support for any of the proposed theories. Error bars depict 95% CIs estimated across participants. Brain surfaces in panel a are from Freesurfer.

Model	Prefrontal ROI	Posterior ROI
Null	-8634.19*	-9131.94*
Time window	-8620.56*	-9125.84*
Duration	-8637.89*	-9138.72*
Time window + Duration	-8624.27*	-9132.71*
Time window + Duration + IIT	-8620.26*	-9125.63*
Time window + Duration + GNW	-8618.65*	-9126.16*
Time window + Duration + IIT x Category	-8577.34*	-9086.74*
Time window + Duration + GNWT x Category	-8575.98*	-9088.87*

Supplementary Table 11. BIC values from the linear mixed model on the gamma activity for each model and ROI. Stars indicate that the fit of the model converged significantly (p-value < .05, two-tailed).

6.2.5 MEG: Alpha power in unified ROIs

Similarly, we tested the predictions on the entire posterior/prefrontal ROIs using alpha power, and observed an initial reduction in activity followed by a sustained but smaller decrease throughout the duration of the stimuli (Supplementary Figure 39). These observations were

subsequently analyzed using a LMM. In the posterior ROI, the response best fitted the GNWT model, different from IIT's predictions. For the prefrontal ROI, the models including the GNWT predictors outperformed all other models (Supplementary Table 12). Taken together, these results support the hypotheses proposed by GNWT and those proposed by IIT.

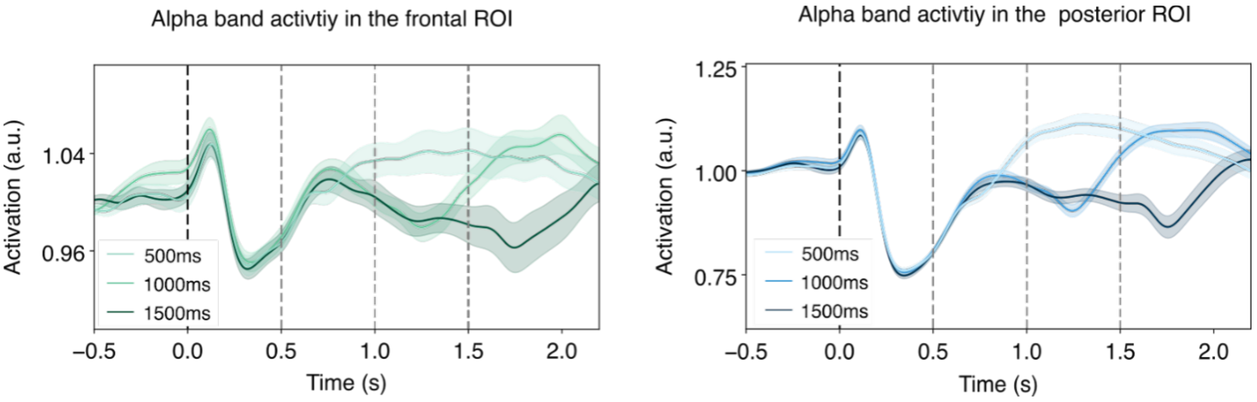

Supplementary Figure 39. Results of the LMM analysis (N=65) on alpha band activity in the entire prefrontal (left, green) and posterior (right, blue) ROIs. Bayesian Information Criterion (BIC) was used to define the winning model. Time series showing averaged alpha band activity across participants for each stimulus duration. Alpha band activity in prefrontal (as well as posterior) cortices was reduced after stimulus onset, and then decreased below baseline levels at stimulus offset, supporting the hypothesis proposed by GNWT. Error bars depict 95% CIs estimated across participants.

Model	Prefrontal ROI	Posterior ROI
Null	-3260.23*	-1814.58*
Time window	-3250.18*	-1817.67*
Duration	-3301.51*	-2017.08*
Time window + Duration	-3291.59*	-2022.05*
Time window + Duration + IIT	-3287.74*	-2018.97*

Time window + Duration + GNW	-3303.48*	-2074.43*
Time window + Duration + IIT x Category	-3262.99*	-2003.74*
Time window + Duration + GNWT x Category	-3276.18*	-2055.83*

Supplementary Table 12. BIC values from the linear mixed model on the alpha activity, separate per models and ROIs. Stars indicate that the fit of the model converged significantly (p<.05, two-tailed).

6.2.6 MEG: ERFs in unified ROIs

When inspecting the combined ROIs, both the prefrontal and posterior ROIs showed an initial evoked response locked to the stimulus onset (Supplementary Figure 40). We also observed an offset response, yet it was mainly visible in the posterior ROI, where neither of the theories predicted such a pattern. A LMM analysis on the ERFs was then performed on each ROI. In both cases, none of the theory models outperformed the time window model (see Supplementary Table 13).

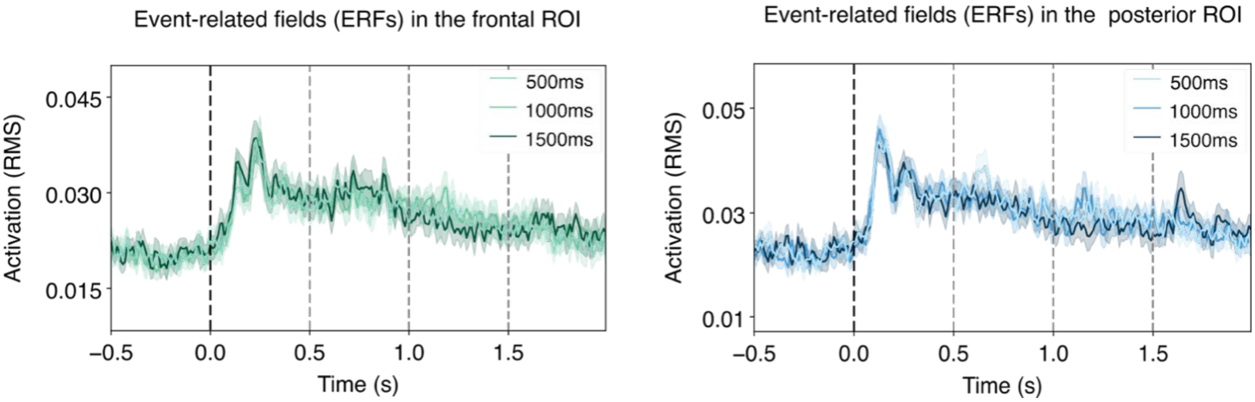

Supplementary Figure 40. Results of the LMM analysis (N=65) on the ERF response in the combined prefrontal (left) and combined posterior (right) ROIs. Bayesian Information Criterion (BIC) was used to define the winning model. Time series showing averaged ERFs across participants for each stimulus duration (Root Mean Square; RMS). We found an early evoked response at the onset of the visual stimuli in both the prefrontal and posterior ROIs and a smaller offset response limited to the posterior ROI. Error bars depict 95% CIs estimated across participants.

Model	Prefrontal ROI	Posterior ROI
Null	-14128.76*	-13689.17*
Time window	-14223.10*	-13726.36*
Duration	-14115.14*	-13673.99*
Time window + Duration	-14209.57*	-13711.19*
Time window + Duration + IIT	-14205.25*	-13706.36*
Time window + Duration + GNW	-14203.24*	-13705.87*
Time window + Duration + IIT x Category	-14170.60*	-13669.54*
Time window + Duration + GNWT x Category	-14168.86*	-13668.17*

Supplementary Table 13. BIC values from the linear mixed model on the ERFs for each model and ROI. Stars indicate that the fit of the model converged significantly (p-value < .05, two-tailed).

6.2.7 MEG: Alpha power using delayed time-windows on the unified ROIs

Given that alpha is a slow rhythm, it might be that the predicted patterns should actually be found later in time (compared to the gamma patterns). We accordingly conducted a control analysis using later time bins. Specifically, we analysed the activity in the time bins 1.0 -1.2 s, 1.5-1.7 s and 2.0-2.2 s. The IIT's model outperformed all other models in both posterior and in the prefrontal ROI. Notably however, this model was not category selective (as opposed to the content-selective IIT x Category model), contrary to IIT's predictions (Supplementary Table 14).

Model	Prefrontal ROI	Posterior ROI

Null	-3482.61*	-1976.74*
Time window	-3473.54*	-1987.24*
Duration	-3524.23*	-2173.01*
Time window + Duration	-3515.32*	-2186.06*
Time window + Duration + IIT	-3525.33*	-2354.12*
Time window + Duration + GNWT	-3511.91*	-2266.14*
Time window + Duration + IIT x Category	-3493.69*	-2341.51*
Time window + Duration + GNWT x Category	-3478.20*	-2248.88*

Supplementary Table 14. BIC values from the linear mixed model on the alpha activity for later time bins, separate per models and ROIs. Stars indicate that the fit of the model converged significantly (p-value < .05, two-tailed).

6.2.8 MEG: Onset/offset analysis

To further investigate alpha band activity in the different parcels in posterior cortex and PFC, we conducted an additional control analysis by comparing the power at either 0.4 or 0.6 s following stimulus onset and offset separately against a baseline at -0.25 s relative to stimulus onset and offset, respectively. These two specific time points were chosen to test both the preregistered and late alpha time bins while attempting to minimize the temporal smoothing of the wavelet analysis which used a 0.5 s gaussian window (i.e., 0.25 s before and after the time point). The baseline before stimulus onset was not used for the offset analysis in an attempt to minimize the contribution of the sustained decreased in alpha activity due to sustained tracking. To increase SNR, the power at each specific time was computed by averaging the activity from -1 to +1 ms, providing a total of three data points. Furthermore, as the onset and offset activity are confounded in the 0.5-s duration, that condition was excluded from the analysis. For this analysis 1.0 and 1.5 s trials from the task irrelevant condition were combined. We considered alpha deactivation to be significant if the p-value in the t-test remained significant after FDR correction over each contrast i.e., onset and offset separately.

In the task irrelevant condition, the onset/offset analysis yielded a significant decrease in alpha band power at stimulus onset in all PFC parcels in the early time. In the late time, the alpha decrease was detected only in the anterior cingulate cortex (Supplementary Figure 41) and orbital inferior frontal gyrus in the late time. All posterior parcels showed an onset response in both early and late time (Supplementary Figure 41). The offset response was significant in 8 out of the 11 PFC parcels in the early time. In the late time window, none of the parcels showed a significant offset response. Similarly, no offset response was detected in the late time in any of the posterior parcels (Supplementary Figure 41), while it was detected in the intraparietal sulcus, inferior temporal gyrus and temporal pole in the early time analysis.

In the task relevant condition, we observed a significant onset response in all posterior and PFC parcels, in both the early and late time periods. However, none of the parcels showed a significant offset response at any time point.

To address the variability in activity during the stimulus presentation, which was used as a baseline in the offset analysis, we conducted an additional control analysis on the task irrelevant condition where an offset response in PFC was detected. We calculated the difference between the alpha power value at each time point and the value estimated at the preceding time point (N-1). This method eliminated any sustained modulation during stimulus presentation, thus making our baseline activity more reliable. All other aspects of the analysis remained the same as in the previous onset-offset analysis. The results demonstrated that once the variability in the sustained response was eliminated, none of the posterior or PFC areas exhibited a significant onset or offset response at 0.4 and 0.6 s, which a phasic offset response occurring in an earlier time period (< 0.25 s) in both posterior cortex and PFC (Supplementary Figure 41).

In summary, the onset/offset analysis shows a phasic offset response in PFC, as well as in posterior cortex, at the preregistered time. These results align with the results of the LMM analysis providing evidence in favor of GNW's predictions. However, the results of the late time analysis - which is considered to take into account the slow dynamic of the alpha band activity - do not fully support this conclusion, as most of the PFC parcels do not show a significant offset response during this period. Moreover, we did not observe an offset response in PFC when analyzing the task relevant condition, nor when controlling for sustained effects in the baseline time period. Finally, given the similarity with the results in posterior cortex, the fact that the offset response in posterior cortex was significantly larger in magnitude (see Supplementary Figure 41 for a comparison), and the known difficulties to record activity from deep and medial (frontal) sources, we cannot rule out the possibility that the effect in PFC stems from leakage from posterior sources (see Extended Data Figure 3b).

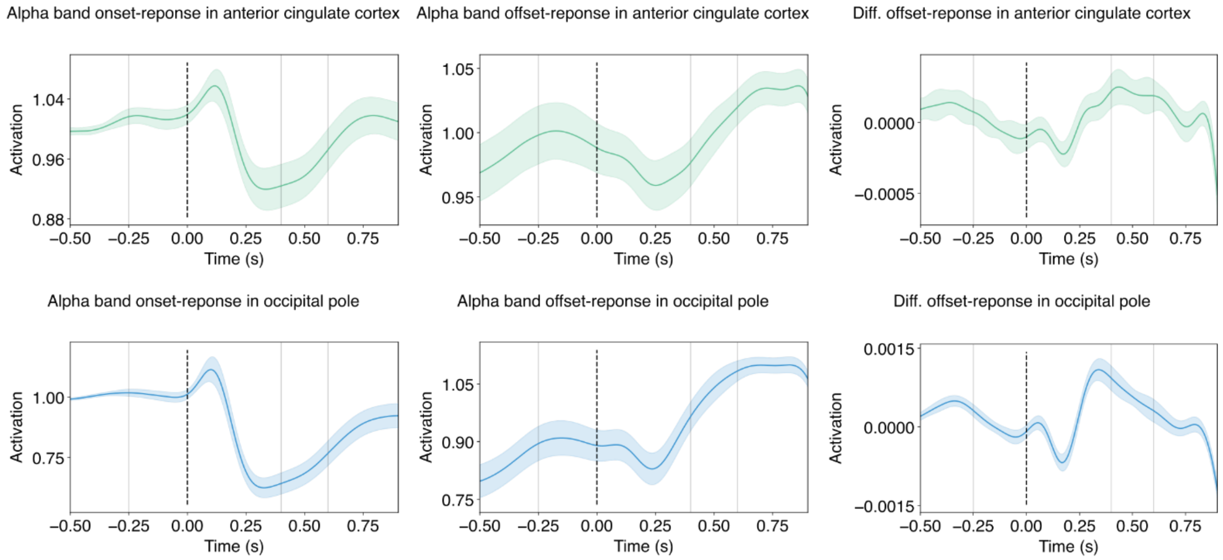

Supplementary Figure 41. Results of the onset/offset analysis (N=65) performed on the alpha band activity in the task irrelevant condition. The top row of the figure presents the alpha band activity in the anterior cingulate cortex locked to stimulus onset (left) and stimulus offset (middle), as well as the offset response after removing the sustained activity (right). The bottom row presents the same plots for the alpha band activity in the occipital pole. Results show a phasic offset response in the anterior cingulate ($p<.05$) at the time predicted by GNWT; however, the late time analysis - which accounts for the slow dynamic of the alpha band activity - does not support this conclusion. Error bars depict 95% CIs estimated across participants.

6.2.9 MEG: Task relevant (non-target) analysis

To examine if the pattern of results might be different when the stimuli are task relevant, we repeated the LMM analysis on the gamma band, alpha band and ERF signals, for each prefrontal and posterior parcel. None of the parcels showed the gamma activity pattern predicted by either of the two theories. As for the alpha band, the GNWT model best fitted the activity in the same parietal parcel where it was found best for the task irrelevant condition (Middle-Posterior Cingulate Cortex), as well as in twelve of the posterior parcels (see Supplementary Figure 42). The non-content selective IIT model best fitted the alpha signal in two posterior parcels (Occipital Pole, Lingual part of the Medial Occipito-Temporal gyrus). When examining the alpha band activity in late time bins, the non-content selective IIT model provided the best fit in the parietal Middle-Posterior Cingulate Cortex, and the frontal Anterior Cingulate Cortex, in the Inferior Orbital Gyrus and in all of the posterior parcels. Finally, while none of the theories' models best fitted the ERF signal in the prefrontal ROIs, the GNWT model outperformed all the other models in three posterior parcels (Cuneus, Lateral Occipito-Temporal Gyrus, Middle Occipital and Lunatus Sulcus).

The analysis on task relevant trials was also performed on the combined ROIs. In the gamma band analysis, none of the theory models outperformed the null model in the prefrontal ROIs and the duration model in the posterior ROIs, in contrast with the theories' predictions. When

considering the alpha band activity, none of the theory models fit the response better than the duration model in the prefrontal ROIs, different from GNWT's predictions; in the posterior ROIs, the GNWT model outperformed the other models, against IIT's predictions. The ERF analysis results were consistent with the other results, with none of the theories' models outperforming the time window model in neither of the ROIs. Altogether, these results showed little evidence in favor of the theories' predictions.

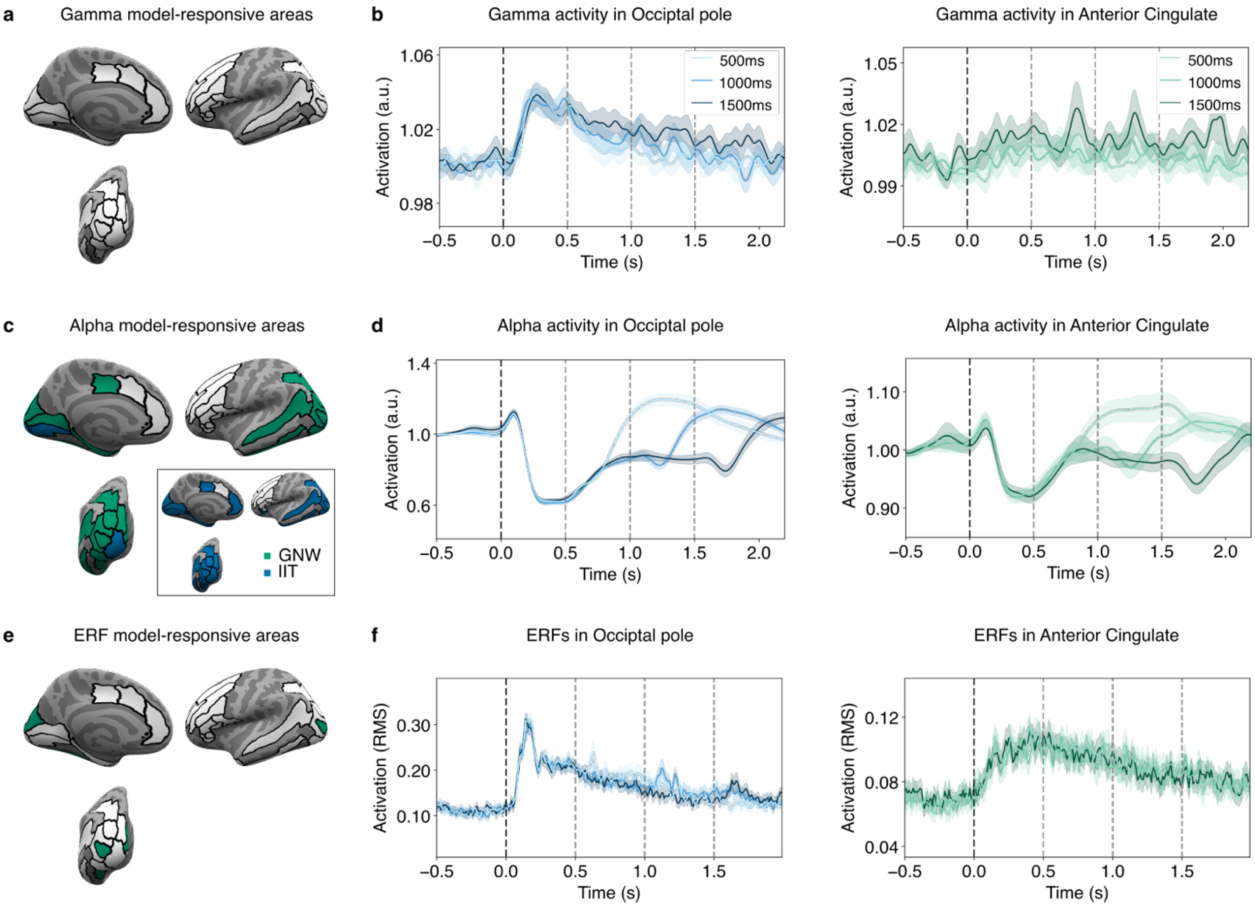

Supplementary Figure 42. Results of the task relevant LMM duration analyses (N=65). Bayesian Information Criterion (BIC) was used in all LMM analyses to identify the winning model.**a.** Results of the LMM analysis on the gamma activity, where no parcel was captured by the theoretical models. **b**. Gamma activity time series in the Occipital pole and Anterior Cingulate Cortex for each duration. **c**. Results of the LMM analysis on the alpha activity. The inset represents the LMM results on the late time bins. **d**. Alpha activity time series in the Occipital Pole and Anterior Cingulate Cortex for each duration. **e**. Results of the LMM analysis on the ERFs. **f**. ERFs time series in the Occipital Pole and Middle-Posterior Cingulate Cortex for each duration. All error bars depict 95% CIs estimated across participants. Brain surfaces in panels a, c, e are from Freesurfer.

6.3 iEEG Pre-registered analyses: Representational Similarity Analysis (RSA)

6.3.1 iEEG: RSA analysis

For this analysis, data from 29 patients with complete data sets were used. Among these patients, 28 had electrodes implanted in the posterior region of the brain, and 28 had electrodes implanted in the prefrontal cortex (PFC) region. In total, there were 583 electrodes in the posterior region and 576 electrodes in the PFC region. We performed a cross-temporal RSA on each theory-defined ROI, separately for each of the stimulus properties (category, identity and orientation). We correlated the temporal generalization matrices with the two theory-predicted matrices using Kendall's Tau correlation and assessed the significance of the correlation value for each theory-predicted matrix through a label shuffling permutation test. For every matrix that was found significant (for either of the theories), we then directly compared the correlation values between the theory-predicted matrices by subtracting the correlation values obtained for the GNWT-predicted matrix from the IIT-predicted matrix, and testing the significance of the difference. Using this approach, a difference greater than zero would indicate an advantage for IIT while a difference smaller than zero would indicate an advantage for GNWT. This allowed us to determine if a theory is empirically validated by performing better than the competing theory within its own ROI. This approach provides a stricter test than simply evaluating the significance of the correlation between a theory's predicted pattern and the observed data. Figure 3 and Extended Data Figure 4 illustrate the results for each of the stimulus properties and theory-defined ROI separately. In Supplementary Table 15, we report the full set of statistical results for each of these tests.

As we report in the main text, in the posterior ROI, all contrasts including category and identity were found to be significantly correlated with the IIT predicted temporal patterns, although for orientation none of the stimulus categories showed a sustained representation, and thus did not correlate with the IIT predicted temporal pattern. When directly contrasting the differences in correlation between the IIT and the GNWT predicted matrix in posterior cortex, we found that faces vs. objects in the task irrelevant condition and object identity were significantly better explained by the IIT predicted temporal pattern. The lack of significant advantage of the IIT vs. GNWT models for the other contrasts, despite the significant correlations with the IIT model itself, is mostly explained by the different type of test applied when directly comparing the two theory predicted matrices against each other. Because there is a partial overlap between the theories' predictions (e.g., at stimulus onset) observing a significant advantage for one theory over the other becomes more challenging. Reflecting this point, GNWT predicts content representation from 0.3-0.5 s following stimulus onset, which was observed in most cases. Yet while the correlation with the GNWT predicted matrix in posterior cortex was mostly found to be positive, it was almost never significant (with the exception of the contrast: task relevant letters vs. false-fonts).

In PFC, none of the contrasts for category, identity or orientation yielded patterns significantly correlated with the GNWT model, which can be explained by the lack of the predicted patterns after stimulus offset. In a few cases, the correlation with the IIT predicted temporal matrix

was found to be significant: namely, faces vs. objects in the task relevant condition, and identity for letters and false fonts; with a significant difference in favor of the IIT predicted temporal pattern over the GNWT predicted temporal pattern for false font identity. These results indicate that at least certain stimulus features might be represented in a sustained fashion in prefrontal regions. Notably, they do not constitute evidence for IIT, as they were observed in PFC and not in posterior cortex as predicted by the theory.

contrast	# electrodes	roi	τ_{iit}	p_{iit}	τ_{gnwt}	p_{gnwt}	$\tau_{iit} - \tau_{gnwt}$	p_{diff}
Faces vs. objects (task irrelevant)	583	posterior	0.62	0.000 *	-0.01	0.507	0.31	0.007 *
Faces vs. objects (task relevant)	583	posterior	0.41	0.004 *	0.09	0.210	0.16	0.127
Letters vs false fonts (task irrelevant)	583	posterior	0.25	0.019 *	-0.06	0.703	0.15	0.106
Letters vs false fonts (task relevant)	583	posterior	0.22	0.033 *	0.20	0.017 *	0.01	0.899
Face identity	583	posterior	0.42	0.003 *	-0.06	0.651	0.24	0.060
Objects identity	583	posterior	0.66	0.000 *	-0.06	0.657	0.36	0.003 *
Letters identity	583	posterior	0.36	0.011 *	0.01	0.473	0.18	0.125
False-fonts identity	583	posterior	0.45	0.002 *	0.07	0.298	0.19	0.109
Face orientation	583	posterior	0.22	0.133	0.00	0.509		
Objects orientation	583	posterior	-0.07	0.561	-0.08	0.729		
Letters orientation	583	posterior	-0.27	0.938	0.05	0.348		
False fonts orientation	583	posterior	-0.20	0.837	0.08	0.287		
Faces vs objects (task irrelevant)	576	PFC	0.02	0.431	0.17	0.051		
Faces vs objects (task relevant)	576	PFC	0.40	0.003 *	0.08	0.246	0.16	0.767
Letters vs false-fonts (task irrelevant)	576	PFC	-0.02	0.541	0.10	0.226		
Letters vs false-fonts (task relevant)	576	PFC	0.13	0.181	-0.01	0.486		
Faces identity	576	PFC	-0.40	0.992	-0.04	0.607		
Objects identity	576	PFC	0.16	0.187	0.17	0.130		
Letters identity	576	PFC	0.45	0.011*	-0.10	0.746	0.28	0.311

False-fonts identity	576	PFC	0.44	0.003*	-0.24	0.971	0.34	0.004 *
Faces orientation	576	PFC	0.07	0.338	0.13	0.215		
Objects orientation	576	PFC	0.02	0.458	-0.03	0.601		
Letters orientation	576	PFC	0.16	0.193	0.20	0.060		
False-fonts orientation	576	PFC	-0.08	0.698	-0.10	0.749		

Supplementary Table 15. Results of the RSA for each of the investigated stimulus properties (category, identity and orientation) in each theory-defined ROI (N patients=28 each). First column: stimulus property studied. Second column: number of electrodes within the theory-defined ROI. Third and fourth column: kendall's Tau correlation between the IIT (τ_{iit}) predicted matrix and the temporal generalization matrices, and associated P-value from the label shuffle permutation test (p_{iit}), respectively. Fifth and sixth column: kendall's Tau correlation and P-value for GNWT (τ_{gnwt}, p_{gnwt}). Seventh and eighth column: 0.5 centered correlation difference between the IIT and GNWT correlation and associated P-value ($\tau_{iit} - \tau_{gnwt}$ and p_{diff} columns). The stars in the P-value column represent the significance of the test at alpha=0.05 (p_{iit} and p_{gnwt}, upper-tail; p_{diff}, two-tailed).

6.4 iEEG Exploratory analyses: RSA

6.4.1 Feature selection

As described above, we had a total of 583 and 576 electrodes in posterior and PFC ROIs, respectively. However, only a fraction of those were found to be responsive to our task (see Onset/offset analysis above). While multivariate analyses are thought to be robust to noise, adding irrelevant features might nonetheless obscure the findings. This in turn might explain the lack of significant representation for some of the contrasts in each ROIs. To rule out this possibility, we performed another RSA analysis, selecting the top 200 most informative features using select-k-best in a cross-validated fashion (5 folds for category and orientation, 3 folds for identity as only 3 trials were available for each label in this contrast level). Supplementary Figure 43 summarizes these results using this feature selection approach and Supplementary Table 16 provides a full description of the results.

In the posterior ROI, we observed strong evidence for IIT-predicted temporal patterns at the level of category: we found a significant correlation difference in favor of IIT for faces vs. objects in the task relevant and irrelevant condition and also for the letters vs. false-fonts contrast in the task irrelevant condition (Supplementary Figure 43, first row). However, for identity level analyses no significant representation was found for any of the stimulus categories, except objects (Supplementary Figure 43, second row). These results diverge from those reported without any feature selection, yet they are easily explained by the low number of trials available for each identity when using feature selection. As feature selection must be cross validated to avoid double dipping, only ⅔ of the trials were used to compute the within class corrected distances, while the

remaining ⅓ were used for feature selection. The increased SNR offered by the feature selection is therefore counteracted by the lower number of trials available for computing the relevant metric. As such, while the RSA matrices both for faces and objects identity significantly correlated with IIT predicted matrices, the difference between the correlation for IIT and GNWT did not reach significance. Finally, for orientation, no significant representation was observed for any of the stimulus categories (Supplementary Figure 43, third row). Interestingly, the temporal generalization matrix for face orientation was observed to be significantly correlated both with IIT and GNWT predicted temporal matrices.

In PFC, feature selection yielded a larger improvement for the representation of category. Consistent with the results found without feature selection, we observed significant faces vs. objects representation in the task relevant and task relevant conditions, as well as significant representation of letters vs. false-fonts in the task relevant condition. For all of those contrasts, information was present only at stimulus onset but not at stimulus offset, contrary to GNWT's predictions (Supplementary Figure 43, fourth row). The RSA patterns themselves were similar to those observed without feature selection. Yet, the latter were not significant at stimulus onset, suggesting that feature selection might confer higher sensitivity to the RSA analysis. These results are consistent with the temporal generalization patterns observed in the decoding analysis.

Though these results suggest category representation exclusively at stimulus onset in PFC, no significant representation for identity or orientation for any of the stimulus categories was observed in PFC, either at stimulus onset or at stimulus offset. Also, the predicted offset patterns were not observed for any of the contrasts (Supplementary Figure 43, fifth and sixth row). Accordingly, none of the temporal generalization matrices were found to be significantly correlated with the GNWT predicted patterns.

Taken together, the RSA analysis with feature selection, while at times improving the statistical significance of certain results, does not change the main conclusions as we found no support for the GNWT prediction of both an onset and an offset reactivation of information.

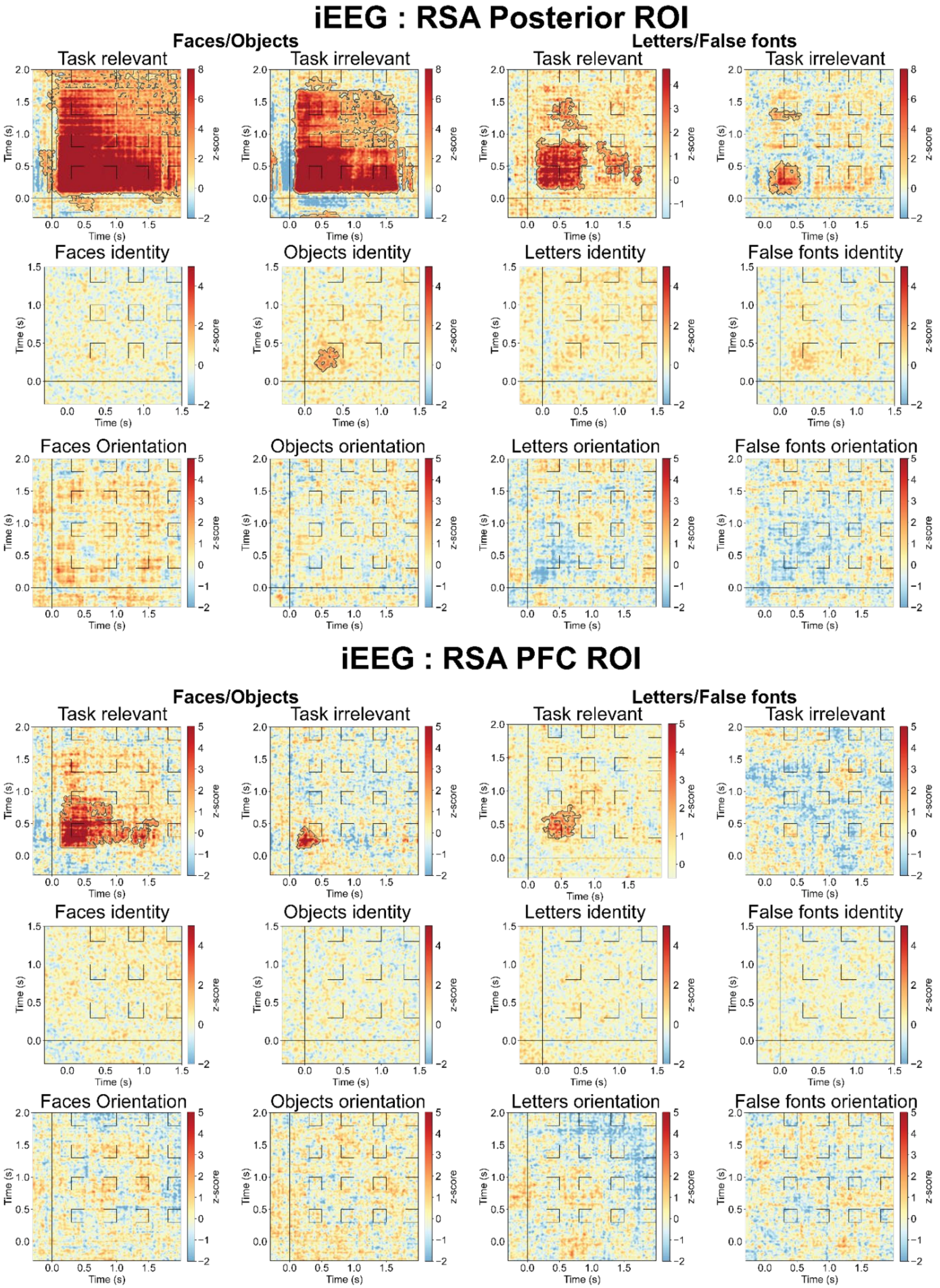
iEEG : RSA Posterior ROI
Faces/Objects
Letters/False fonts
Task relevant
Task irrelevant
Faces identity
Objects identity
Letters identity
False fonts identity
Faces Orientation
Objects orientation
Letters orientation
False fonts orientation
iEEG : RSA PFC ROI
Time (s)
z-score

Supplementary Figure 43. Results of temporal generalization RSA with 200 features selection (N=28). The upper three rows show the results from the posterior ROIs, the lower three rows show the results from the PFC ROIs. For each ROI, results are reported for category level contrasts (first row), identity level contrast for each category separately (second row) and orientation level contrasts for each category separately (last row). For the category level contrasts, task relevant and irrelevant trials were investigated separately. For identity and orientation contrasts, task relevant and irrelevant trials were combined. Furthermore, for category and orientation, only 1.5 s trials were analyzed. For identity, 1.0 s and 1.5 s trials were combined due to the low number of trials across patients. As a result of this, the X-axis for the category and orientation conditions differs from the one for identity. The contour in the matrices depicts the statistically significant clusters (p<0.05, upper-tail) determined using cluster-based permutation.

contrast	# features	roi	τ_{iit}	p_{iit}	τ_{gnwt}	p_{gnwt}	$\tau_{iit} - \tau_{gnwt}$	p_{diff}
Faces vs. objects (task irrelevant)	200	posterior	0.61	0.000 *	0.00	0.452	0.30	0.002 **
Faces vs. objects (task relevant)	200	posterior	0.51	0.000 *	0.03	0.342	0.24	0.007 **
Letters vs false fonts (task irrelevant)	200	posterior	0.29	0.002 *	-0.07	0.792	0.18	0.019 **
Letters vs false fonts (task relevant)	200	posterior	0.28	0.003 *	0.15	0.041 *	0.07	0.388
Face identity	200	posterior	0.24	0.012 *	-0.03	0.619	0.13	0.153
Objects identity	200	posterior	0.24	0.012 *	0.17	0.046 *	0.04	0.654
Letters identity	200	posterior	-0.09	0.743	0.09	0.190		
False-fonts identity	200	posterior	0.23	0.102	0.00	0.489		
Face orientation	200	posterior	0.21	0.032 *	0.20	0.029 *	0.00	0.983
Objects orientation	200	posterior	-0.09	0.636	-0.04	0.644		
Letters orientation	200	posterior	-0.27	0.965	0.07	0.256		
False fonts orientation	200	posterior	-0.17	0.814	0.08	0.249		
Faces vs objects (task irrelevant)	200	PFC	0.03	0.355	0.17	0.050	-0.07	0.461
Faces vs objects (task relevant)	200	PFC	0.45	0.001 *	0.08	0.214	0.19	0.458
Letters vs false-fonts (task irrelevant)	200	PFC	-0.01	0.506	0.08	0.224		

Letters vs false-fonts (task relevant)	200	PFC	0.14	0.140	0.03	0.365		
Faces identity	200	PFC	-0.26	0.990	0.02	0.409		
Objects identity	200	PFC	0.09	0.200	0.04	0.336		
Letters identity	200	PFC	0.24	0.049 *	0.02	0.435	0.11	1.000
False-fonts identity	200	PFC	0.26	0.010 *	-0.05	0.653	0.15	0.456
Faces orientation	200	PFC	0.40	0.001 *	-0.25	0.985	0.32	0.003 *
Objects orientation	200	PFC	-0.02	0.563	0.00	0.471		
Letters orientation	200	PFC	0.14	0.176	0.20	0.044 *	-0.03	1.000
False-fonts orientation	200	PFC	-0.06	0.685	-0.10	0.786		

Supplementary Table 16. Results of the RSA for each of the investigated stimulus properties (category, identity and orientation) in each theory-defined ROI with 200 features selection.

6.4.2 GNWT extended windows

The investigated time windows were determined based on theoretical considerations, and were aimed at maximizing the difference between the theories' predictions. In particular, for IIT it was critical that the tested time windows did not include the stimulus evoked response as much as possible, while for GNWT it was critical to capture the late responses marking the predicted ignition (i.e., >0.25 s). As a compromise between those two competing needs, a time window starting at 0.3 s was chosen. However, upon inspecting the data, we observed several cases where the responses in PFC had earlier latencies than the preregistered time window (0.3-0.5 s), yet still within the range of latencies predicted by GNWT (<0.25 s). To rule out the possibility that the lack of evidence for GNWT simply stemmed from the selection of the time window, we performed an exploratory analysis on the 1.5 s stimulus duration trials, using a more extended and earlier time window to capture a representation of conscious content in the workspace, had it occurred (from 0.25-0.5 s for GNWT onset ignitions and 1.75-2.0 s for GNWT offset ignitions, and from 0.25-1.5 s for IIT's sustained activity prediction). This also enabled us to investigate whether the offset responses predicted by GNWT may have also occurred earlier. Since this analysis only tested GNWT's prediction, it was only carried out in the PFC ROIs.

Results from this extended time window analysis are described in Supplementary Table 17. The observed findings did not change the main conclusions (as shown in Supplementary Table 15). Notably, for the category representation of faces/objects in task irrelevant trials, we found a significant correlation with the GNWT predicted matrix but not with the IIT predicted matrix, yet the direct contrast between the two theory predicted matrices was not statistically significant, thus failing the predefined test. Notably, this correlation seems to be driven by off-diagonal non-significant increases in within-class corrected distances, while GNWT predicts generalization between stimulus onset and offset, which should be observed also along the diagonal in the

predicted time window (from 1.75-2.0 s). Thus, even if the statistical test would have yielded a significant result, the results would not be entirely compatible with the GNWT predicted offset patterns.

contrast	# electrodes	roi	τ_{iit}	p_{iit}	τ_{gnwt}	p_{gnwt}	$\tau_{iit} - \tau_{gnwt}$	p_{diff}
Faces vs objects (task irrelevant)	576	PFC	0.00	0.479	0.26	0.004 *	-0.13	0.617
Faces vs objects (task relevant)	576	PFC	0.45	0.000 *	0.02	0.403	0.22	0.767
Letters vs false-fonts (task irrelevant)	576	PFC	-0.06	0.657	0.09	0.224		
Letters vs false-fonts (task relevant)	576	PFC	0.13	0.166	0.01	0.431		
Faces identity	576	PFC	-0.37	0.990	-0.03	0.577		
Objects identity	576	PFC	0.19	0.130	0.19	0.087		
Letters identity	576	PFC	0.41	0.015 *	-0.17	0.870	0.29	0.311
False-fonts identity	576	PFC	0.18	0.137	0.14	0.190		
Faces orientation	576	PFC	0.43	0.005 *	-0.22	0.966	0.33	0.004*
Objects orientation	576	PFC	0.03	0.398	-0.05	0.656		
Letters orientation	576	PFC	0.19	0.139	0.17	0.072		
False-fonts orientation	576	PFC	-0.11	0.778	-0.06	0.661		

Supplementary Table 17. Results of the RSA for each of the investigated stimulus properties (category, identity and orientation; N patients=28 for each ROI), when extending the predicted time-windows by 0.05 s, to explore the possibility of offset responses at earlier latencies than expected.

6.4.3 GNWT model including only onset

GNWT's preregistered prediction stated that the update of the workspace following stimulus offset should reinstate the information conveyed by the stimulus that has just disappeared. However, one might argue instead that an alternative mechanism for maintaining conscious perceptions over time only requires an update conveying information about the next conscious content. Under this interpretation of GNWT, information about the content of consciousness will be represented transiently following the onset of the stimulus only, without requiring an offset response.

We tested this prediction using an alternative GNWT derived matrix, where representation

is predicted to occur only from 0.3-0.5 s (post stimulus onset). The results are described in Supplementary Table 18. Notably, while all category level contrasts were found to be significantly correlated with this GNWT 'onset only' model, and only one of the four category contrasts (faces vs. objects, task relevant) was correlated with the IIT model, we did not observe any significant differences between the IIT and GNWT models for any of the category contrasts when they were directly compared, indicating no stronger support for the GNWT prediction over the IIT prediction or vice versa.

With respect to identity, significant correlations were evident for both the GNWT and IIT models for letters identity, yet with no stronger support for GNWT when directly compared to IIT. No other identities (i.e., within the face, object, or false-font category) showed any significant correlations with either theory's model.

For orientation, we found no support for a GNWT onset-only model for any of the stimulus categories, and a significant correlation with the IIT model only for face orientation. As mentioned above in the preregistered RSA analysis section, these results do not lend support to IIT as they were observed in PFC and not in the posterior cortex.

contrast	# electrodes	roi	τ_{iit}	p_{iit}	τ_{gnwt}	p_{gnwt}	$\tau_{iit} - \tau_{gnwt}$	p_{diff}
Faces vs objects (task irrelevant)	576	PFC	0.02	0.431	0.26	0.002 *	-0.12	0.617
Faces vs objects (task relevant)	576	PFC	0.40	0.003 **	0.35	0.000 **	0.02	0.767
Letters vs false-fonts (task irrelevant)	576	PFC	-0.02	0.541	0.21	0.042 *	-0.11	1.000
Letters vs false-fonts (task relevant)	576	PFC	0.13	0.181	0.34	0.000 *	-0.10	1.000
Faces identity	576	PFC	-0.40	0.992	-0.35	0.997		1.000
Objects identity	576	PFC	0.16	0.187	0.14	0.177		1.000
Letters identity	576	PFC	0.45	0.011 *	0.28	0.043 *	0.08	0.318
False-fonts identity	576	PFC	0.07	0.338	0.17	0.146		1.000
Faces orientation	576	PFC	0.44	0.003 *	-0.11	0.767	0.27	0.004**
Objects orientation	576	PFC	0.02	0.458	0.03	0.391		1.000
Letters orientation	576	PFC	0.16	0.193	0.11	0.209		1.000
False-fonts orientation	576	PFC	-0.08	0.698	-0.10	0.727		1.000

Supplementary Table 18. Results of the RSA for each of the investigated stimulus properties (category, identity and orientation) in the PFC ROIs (N patients=28), testing a GNWT model which includes only a representation of information only at stimulus onset.

6.4.4 iEEG cross-task decoding at stimulus offset

To investigate the decodability of stimulus category at stimulus offset, which was relevant to one of the predictions made by GNWT, we trained separate classifiers on electrodes from posterior and prefrontal ROIs (GNWT ROIs $N_{electrodes}$=576, IIT ROIs $N_{electrodes}$=583). Data from all stimulus durations were combined and aligned to the stimulus offset (-0.5 to 0.5 s) for each duration (0.5, 1.0, 1.5 s). Classifiers were trained to discriminate stimulus category (faces vs. objects) in the task irrelevant condition at each time-point and tested in the task relevant condition across all time-points. Significant decoding of stimulus category (faces vs. objects) was observed in the posterior ROI extending to approximately 0.3 s after stimulus offset (Supplementary Figure 44). In the prefrontal ROI, decoding of stimulus category after stimulus offset was not observed.

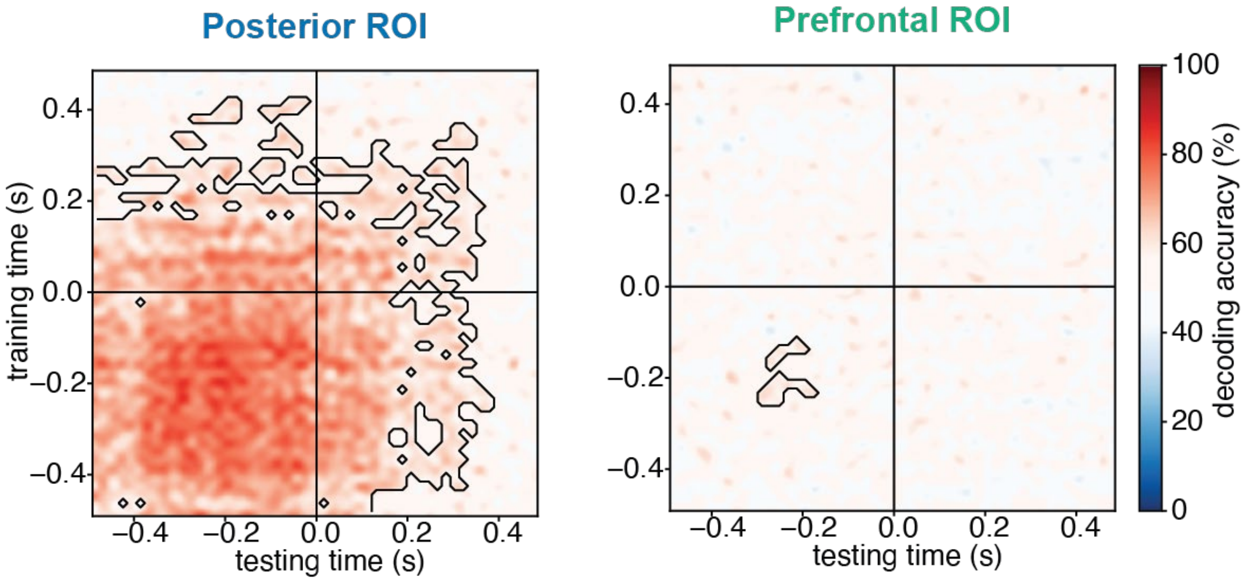

Supplementary Figure 44. Cross-task temporal generalization of decoding aligned to stimulus offset (-0.5 to 0.5 s) for iEEG (N=28). Pattern classifiers were trained to discriminate stimulus category (faces vs. objects) in the task irrelevant condition at each time-point and tested in the task relevant condition across all time-points (left: posterior ROIs; right: prefrontal ROIs). All trials from different durations were aligned to the stimulus offset (which is marked as time 0 in this figure). Contours in the matrices depict statistically significant clusters ($p<0.05$) determined using cluster-based permutation.

6.5 MEG Pre-registered analyses: Representational Similarity Analysis (RSA)

6.5.1 MEG RSA analysis

We performed cross-temporal RSA on MEG cortical time series data, using the same methods as iEEG. For RSA of category and orientation, only 1.5s duration trials were entered into the analysis. We also used pseudotrial aggregation. For RSA of identity we combined 1.0 s and 1.5 s duration trials to compensate for the lower number of repetitions per identity. Pseudotrials

were not applied for the identity analyses also due to too low number of trials (less than 20) for each identity.

Neither the posterior ROI nor prefrontal ROI exhibited a pattern consistent with the theory predictions. In the posterior ROI (Supplementary Figure 45), we observed information about category (faces vs. objects) only at stimulus onset, while category information for letters vs. false fonts was absent in the task irrelevant condition. Information about orientation was not observed for any of the four stimulus categories investigated. Identity information was observed for objects, letters and false fonts but not for faces. For the prefrontal ROI (Supplementary Figure 46), other than false font identity (only significant at the early time window of stimulus onset) we did not observe any information for any of the stimulus properties (category, orientation, identity).

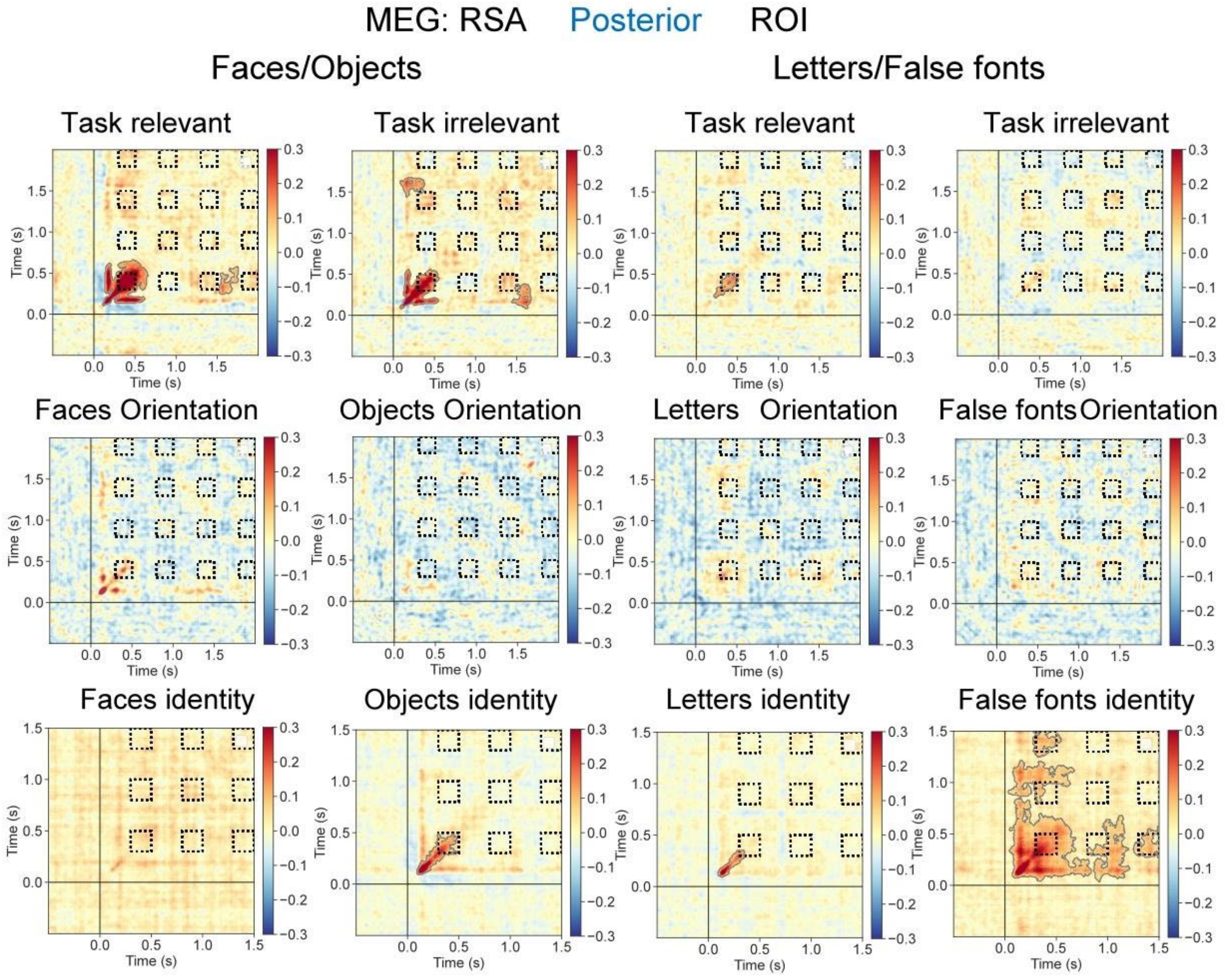

Supplementary Figure 45. Results of temporal generalization RSA for posterior ROIs. For each ROI, results are reported for category level contrasts (top row), orientation level contrasts for each

category separately (middle row) and identity level contrast for each category separately (bottom row). For the category level contrasts, task relevant and irrelevant trials were investigated separately. For identity and orientation contrasts, task relevant and irrelevant trials were combined. Furthermore, for category and orientation, only 1.5 s trials were analyzed. For identity, 1.0 s and 1.5 s trials were combined due to the low number of trials for each identity. The contour in the matrices depicts the statistically significant clusters (p<0.05, one-tailed) determined using cluster-based permutation (N=65).

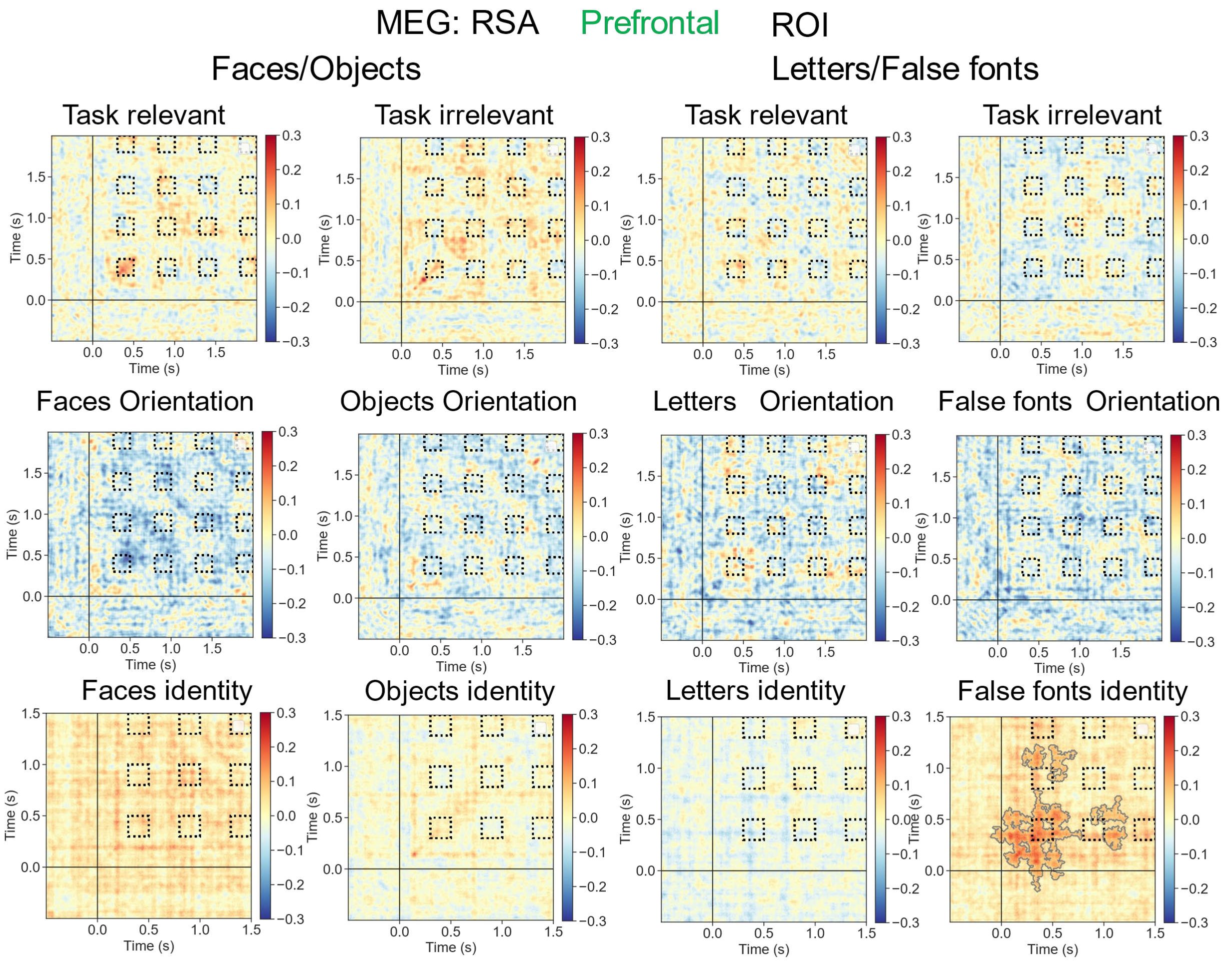

Supplementary Figure 46. Results of temporal generalization RSA for Prefrontal ROIs. Same conventions , statistics and sample as in Supplementary Figure 45.

7. Prediction #3: Interareal functional connectivity

In the main paper, we report the connectivity results for the task irrelevant trials, which constitute the most critical test for the theories. Here, we further describe analyses conducted either on the task relevant condition, where the signal is expected to be stronger due to task-based attentional amplification, or on the combined data from both task conditions, thus improving the signal-to-noise ratio by doubling the number of trials. These additional analyses allowed us to increase the chances of finding the predicted results, as well as to examine how one's choice of different tests influences the assessment of the predicted patterns. Since the fMRI analysis reported in the main text was already conducted across task conditions (i.e., combining all trials), we only report additional results from the iEEG and MEG analyses here (after first providing supplementary results for the fMRI gPPI analysis reported in the main paper).

7.1 Pre-registered analyses

7.1.1 fMRI Generalized Psycho-Physiological Interaction (gPPI) Table

In the main paper, we reported the results of the Generalized Psycho-Physiological Interaction (gPPI) analysis, combining task relevant and irrelevant trials. Supplementary Table 19 provides the full set of results. It shows that several regions such as Inferior Frontal Gyrus, Intra-Parietal Sulcus, Cuneus, and V1/V2 showed content-specific connectivity with the FFA seed. No significant clusters were observed when investigating connectivity with the FFA seed separately for task relevant and irrelevant conditions. Extended Data Figure 6e-f shows the clusters at an uncorrected $p<0.01$. Notably, no significant clusters were observed with the seed in Lateral Occipital Cortex, either when combining task relevant or irrelevant trials, or when performing the analysis separately per task.

**Anatomical ROIs (Destrieux atlas)**	**Task relevant and task irrelevant trials combined**	
	n voxels	% voxels
**Posterior ROI**		
G_and_S_occipital_inf	0	0
G_oc-temp_lat-fusifor	0	0
G_occipital_middle	2	0.008
S_oc_middle_and_Lunatus	12	1.188
G_cuneus	294	11.732
G_occipital_sup	32	1.621
G_oc-temp_med-Lingual	115	3.836
G_oc-temp_med-Parahip	0	0

G_temporal_inf	0	0
Pole_occipital	78	3.161
Pole_temporal	0	0
S_calcarine	204	8.409
S_intrapariet_and_P_trans	463	12.207
S_oc_sup_and_transversal	12	0.848
S_temporal_sup	0	0
**PFC ROI**		
G_and_S_cingul-Mid-Post	0	0
Lat_Fis-ant-Horizont	0	0
Lat_Fis-ant-Vertical	0	0
G_and_S_cingul-Ant	0	0
G_and_S_cingul-Mid-Ant	0	0
G_front_inf-Opercular	0	0
G_front_inf-Orbital	0	0
G_front_inf-Triangul	122	7.345
G_front_middle	22	0.358
S_front_middle	0	0
S_front_sup	0	0
S_front_inf	68	3.274

Supplementary Table 19. Number and percentage of voxels in each ROI found significant in the gPPI analysis with combined task relevant and task irrelevant trials using FFA as a seed.

7.1.2 Task Relevant condition

7.1.2.1 Pre-registered analyses

The same Pairwise Phase Consistency (PPC) analysis was conducted solely on task relevant trials (See Supplementary Figure 47). In the iEEG data, cluster-based permutation tests revealed a significant difference in synchronization between face-selective and object selective electrodes and V1/V2 electrodes. This effect was found in an early time window and in a low-frequency band, in line with what was found in the task irrelevant condition. These effects were mostly explained by the synchronous activity elicited by the stimulus evoked response (Supplementary Figure 47 a-b, top row). In contrast, no content-selective PPC was found between face- and object-selective electrodes and PFC in the relevant time window (Supplementary Figure 47 a-b, bottom row).

In the MEG source data, cluster-based permutation tests revealed a significant difference in content specific synchronization between category selective nodes and V1/V2 in the task relevant condition: higher synchronization was found between face-selective nodes and V1/V2 for face stimuli, which remained significant even after removing the stimulus evoked response. We also found content-specific synchronization between face-selective nodes, object-selective nodes and PFC. Removing the evoked response reduced but did not completely abolish this synchronization. Notably, both of these synchronization effects (between category-selective areas and V1/V2 or PFC) were found in low-frequency bands in early time-windows.

Overall, compared to the main analyses on task irrelevant trials, the results of the PPC analysis on task relevant ones showed stronger modulations in content-specific synchronization. However, after removing the evoked response, the observed effects were too early in time, around the onset of the stimulus, to be considered meaningful, particularly in relation to V1/V2.

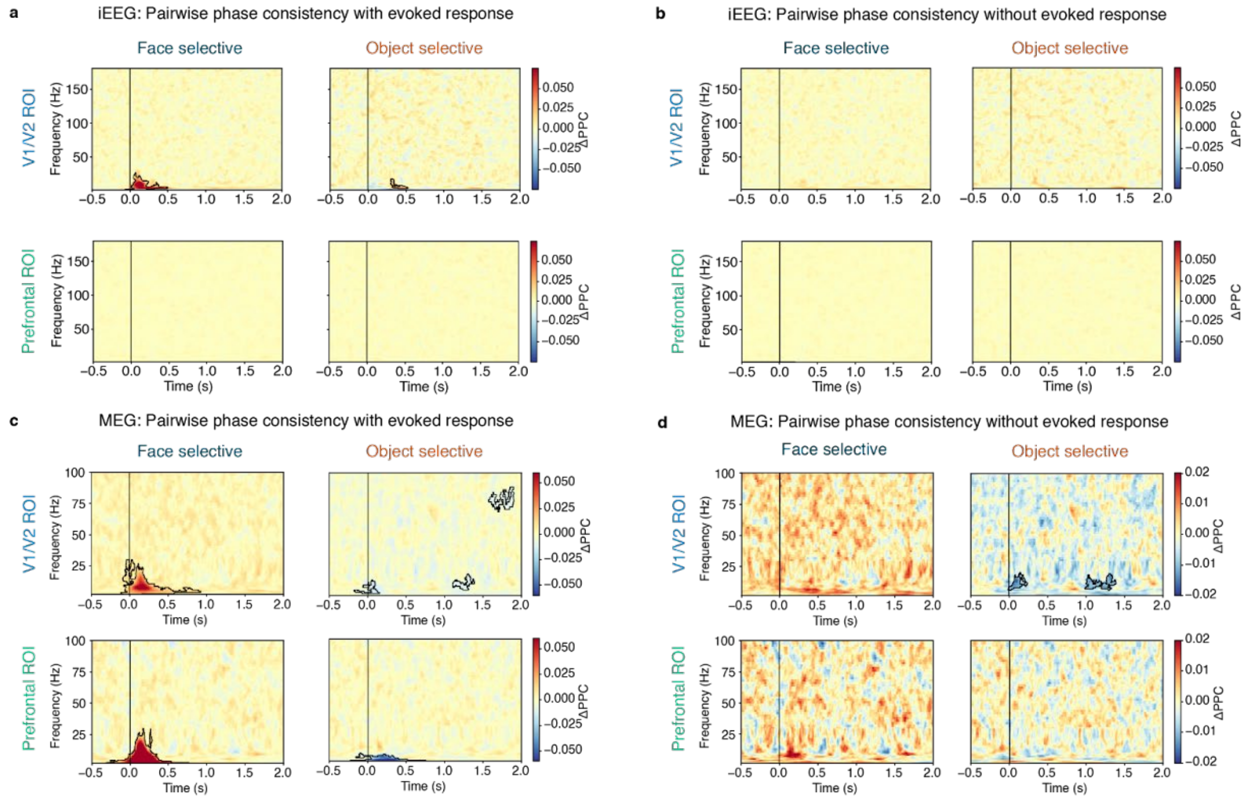

Supplementary Figure 47. Results of the PPC analysis (cluster based permutation tests, $p<0.05$, two-tailed) on task relevant trials before and after removing the evoked response on iEEG and MEG source data. **a** iEEG PPC analysis of task relevant trials revealed significant content-selective synchrony (faces > objects for face-selective electrodes; objects > faces for object-selective electrodes) in V1/V2 ROIs (top row, face-selective: $N_{patients}=4$, $N_{electrodes}=30$, object-selective: $N_{patients}=4$, $N_{electrodes}=21$), but not in PFC ROIs (bottom row, face-selective: $N_{patients}=19$,

$N_{electrodes}$=81, object-selective: $N_{patients}$=14, $N_{electrodes}$=57). **b.** After regressing out the evoked response, iEEG showed no significant content-selective connectivity in task relevant trials. **c.** MEG PPC analysis (N=65) of task relevant trials revealed significant category-selective synchrony below 25 Hz for the face-selective GED filter (i.e., faces > objects for face-selective electrodes) in V1/V2 (top row) and PFC ROIs (bottom row) and for object-selective synchrony (objects > faces for object-selective electrodes) in PFC only. **d.** Removing the evoked response from MEG data significantly reduced but did not completely abolish the synchronization.

7.1.2.2 Exploratory analyses: DFC

The task relevant trials were also analyzed using the Dynamic Functional Connectivity (DFC) method[8], with the same parameters as the PPC analysis, including restricting the analysis to the intermediate (1.0 s) and long (1.5 s) duration trials (see methods). The results are described in Supplementary Figure 48.

In iEEG, we observed significant connectivity between face selective electrodes and V1/V2. Connectivity was sustained up to 1 s after stimulus onset and present across several frequency bands, most predominantly in the gamma band between 50-100 Hz. Significant connectivity between object selective electrodes and V1/V2 was also observed predominantly in the gamma band, but it was briefer, lasting up to 0.5 s after stimulus onset. Significant, content-specific connectivity between face-selective electrodes and PFC was also observed, spanning a range of frequencies from the beta band up to the HG band, which was also extended in time up to 1 s after stimulus onset. In contrast, DFC between object selective electrodes and PFC was spottier and briefer, with an initial, peak in the HG range up until ~0.4 s, followed by a brief increase in the beta/low gamma range around 0.8 s after stimulus onset (but stronger for face stimuli). These effects were not entirely explained by the synchronous activity elicited by the stimulus evoked response as they remain after regressing out the evoked response (Supplementary Figure 48b).

For the MEG cortical time series, the results of the cluster-based permutation tests on the data (without regressing out the evoked response) revealed a significant difference between conditions for all nodes in the time window of 0 to 0.5 s from stimulus presentation (Supplementary Figure 48c). Specifically, we observed a significant increase in content-specific DFC in the low frequency range between the face-selective node and both PFC and V1/V2. This was accompanied by a simultaneous reduction in content-specific DFC in the high frequency range. We also found a smaller but significant modulation in synchrony between the object-selective node and both PFC and V1/V2 within the first 0.5 s after stimulus onset, mainly in the low frequency range.

To further investigate the stability of these results, we repeated the MEG DFC analysis after removing the evoked response, which largely removed the changes in connectivity observed at high frequencies while leaving virtually intact those observed at low frequencies (Supplementary Figure 48d). DFC between V1/V2 and object selective nodes was sustained up to 1.0 second in the alpha band, while briefer in time, lasting up to 0.5 seconds, between face-selective nodes and V1/V2. A comparable pattern of connectivity was observed between PFC and face-selective nodes. Overall, DFC was more pronounced between the face-selective node and

both PFC and V1/V2 than in the object-selective node, in line with what we observed in the main analysis on task irrelevant trials.

These findings demonstrate that including the evoked responses can affect the DFC analysis by altering the high frequency synchronization, as well as potentially blocking the observation of smaller non-evoked synchronization patterns that are only noticeable if the evoked response is removed. This should accordingly be taken into account in future studies.

Overall, the results of our PPC analyses did not provide convincing evidence for either theory's prediction of either sustained (IIT) or phasic (GNWT) connectivity between relevant theory ROIs and content-selective nodes. Most of the results either showed no connectivity, or connectivity attributed to the evoked response and thus not related to content-specific synchrony. On the other hand, the power-based DFC analysis did show evidence of content-specific responses, but were either inconsistent between the iEEG and MEG modalities, or did not align with either theory's prediction. Therefore, no conclusive argument could be drawn for either theory's prediction.

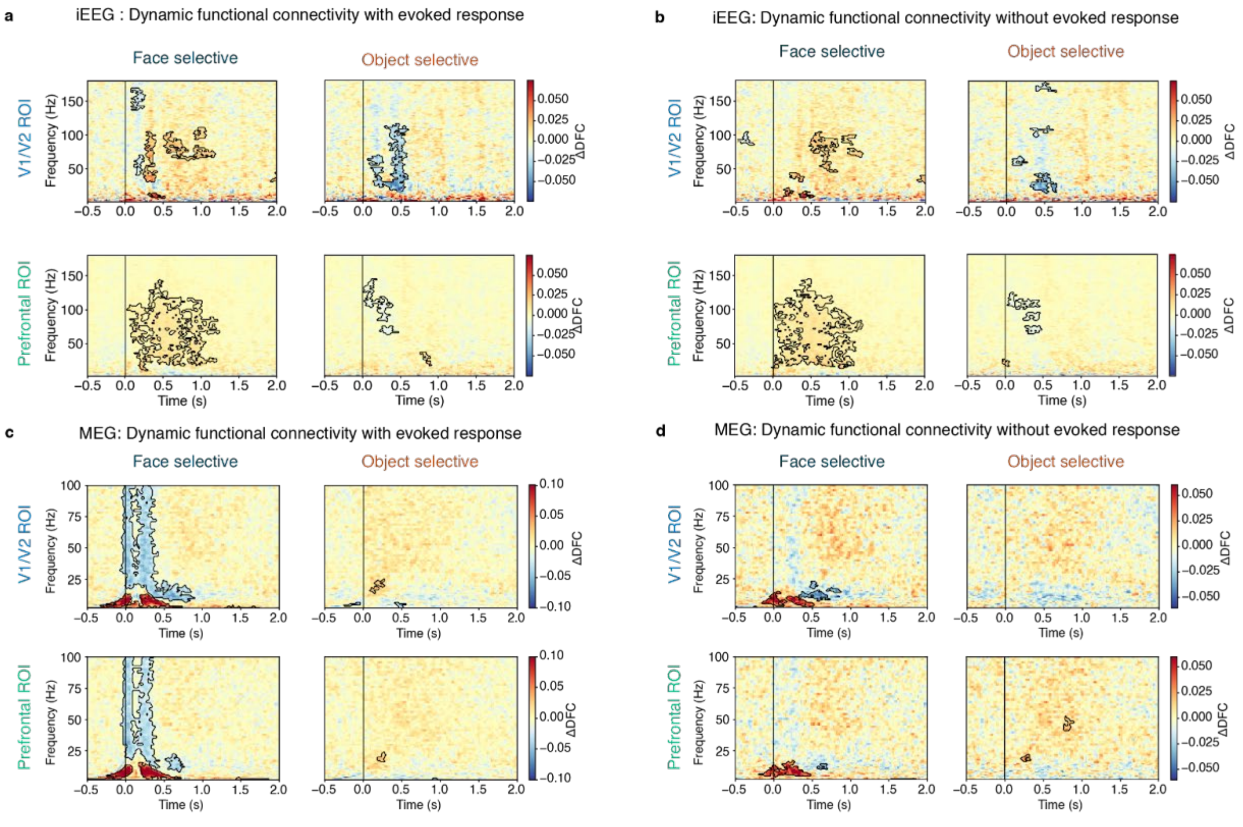

Supplementary Figure 48. Results of the DFC analysis (cluster based permutation tests, p<.05, two-tailed) on task relevant trials before and after removing the evoked response on iEEG and

MEG source data. **a**. iEEG connectivity showed sustained (0-1 s) synchrony for face-selective electrodes to both V1/V2 regions (face-selective: $N_{patients}$=4, $N_{electrodes}$=30, object-selective: $N_{patients}$=4, $N_{electrodes}$=21) and PFC (face-selective: $N_{patients}$=19, $N_{electrodes}$=81, object-selective: $N_{patients}$=14, $N_{electrodes}$=57). **b**. iEEG synchrony remained largely intact after removing the evoked response, but with reduced synchrony during the initial 0- 0.5s window for the V1/V2 ROI. **c**. MEG connectivity (N=65) showed low-frequency DFC (< 25 Hz) between the face-selective node and both V1/V2 and PFC during the initial 0-0.5 s time window for faces (red) and high-frequency DFC (25-100 Hz) for objects (blue) in these same face-selective nodes. **d**. Removing the evoked response from MEG data abolished the high-frequency connectivity, while largely preserving the low-frequency effects, and revealing some object-selective (blue) connectivity in the alpha-band.

7.1.3 Task Relevant and Task Irrelevant combined

7.1.3.1 Pre-registered analyses

In the main paper, we reported the iEEG and MEG results on connectivity focusing on the task irrelevant trials, as those were the most diagnostic to testing the theories' predictions. In the supplement above, we repeated these analyses for the task relevant condition. However, as removing the evoked response from the single trials yielded no consistent connectivity either with V1/V2 or PFC, we conducted the same analyses combining task relevant and task irrelevant trials. The aim of this control analysis was to maximize statistical power by increasing the number of trials. We report the PPC results before and after subtracting the evoked responses from the single trials both for iEEG and MEG (Supplementary Figure 49). For the iEEG data, PPC results were comparable to those found in the analyses conducted separately on the task irrelevant and relevant trials: early content-specific synchronization was observed between face and object selective electrodes and V1/V2 predominantly in a low frequency band. However, this effect was abolished when removing the evoked response. No significant synchronization between PFC and face or object selective electrodes was found. This was true regardless of whether the evoked responses were removed from the data.

In MEG source data, cluster-based permutation tests showed a significant difference in low-frequency phase-synchronization between the face-selective node and PFC and V1/V2 in the period right after stimulus onset. A significant difference was also detected between the object-selective node and V1/V2. However, this effect was not sustained throughout presentation of the stimuli (1.5 s), and instead appeared 0.5 s after stimulus onset. Removing the evoked response again significantly reduced the synchronization and in some cases completely removed it (e.g., face-selective nodes and V1/V2 connectivity and the object-selective node and PFC connectivity).

Overall, the results of the phase-synchronization analyses on iEEG and MEG source data on the combined task relevant and task irrelevant conditions did not provide clear support for either GNWT or IIT, akin to the main conclusion of the analysis conducted on the task irrelevant condition. Specifically, the results provide weak evidence for a content-specific modulation of synchronization between the category-selective nodes and both the PFC and the V1/V2 ROIs. Connectivity with V1/V2 was however not sustained, and observed mostly in low-frequencies <25 Hz, in contrast with IIT's predictions. In contrast, the MEG data revealed synchronization between PFC and the face-selective node at the ignition time window, as predicted by GNWT. However,

this effect appears to be driven mostly by the evoked responses, as after removal of the evoked response, the synchronization that remained was earlier than the predicted GNWT time-window of ignition, i.e., ~0.1-0.3 s.

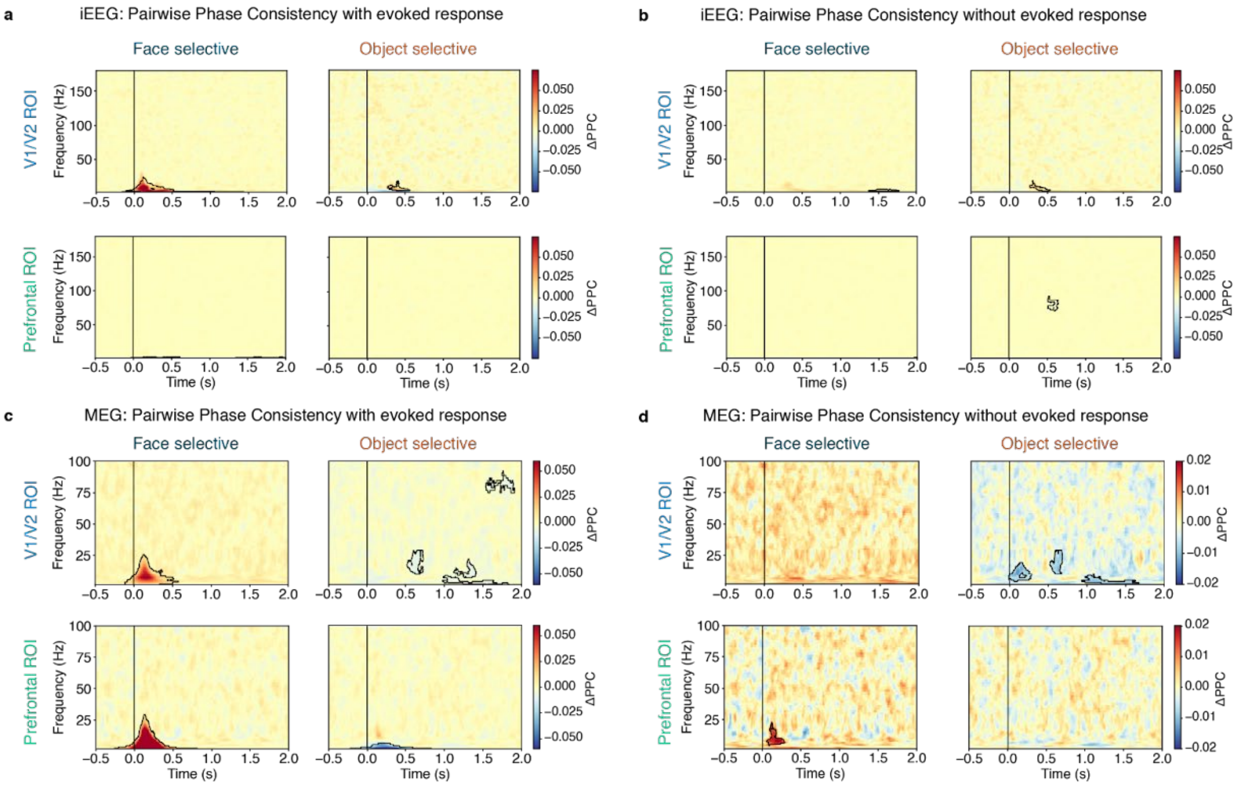

Supplementary Figure 49. Results of the PPC analysis (cluster based permutation tests, p<.05, two-tailed) on the combined task relevant and task irrelevant trials, before and after removing the evoked response on iEEG and MEG source data. **a-b**. iEEG results remained consistent with those found when analyzing the task irrelevant and task relevant conditions separately in V1/V2 regions (face-selective: $N_{patients}$=4, $N_{electrodes}$=30, object-selective: $N_{patients}$=4, $N_{electrodes}$=21) and PFC (face-selective: $N_{patients}$=19, $N_{electrodes}$=81, object-selective: $N_{patients}$=14, $N_{electrodes}$=57). **c-d**. MEG results (N=65) were also consistent with those obtained from separate analyses on task irrelevant and task relevant data.

7.1.3.2 Exploratory analyses: DFC

Following the DFC analysis reported in the main text for task irrelevant trials and above for task relevant trials, we ran the same analysis on the combined task relevant and task irrelevant trials to increase statistical power (Supplementary Figure 50). In iEEG, we observed significant connectivity between face-selective electrodes and V1/V2. Connectivity was sustained up to 1

second after stimulus onset and present across several frequency bands, most predominantly in the high-gamma band between 70-100 Hz. Significant connectivity between object-selective electrodes and V1/V2 was also observed over a broad frequency range (e.g., > 30Hz), but only up to 0.5 s after stimulus onset. Significant, content-specific connectivity between face-selective electrodes and PFC was also observed, spanning a range of frequencies from the beta band up to the HG band, which was also extended in time up to 1 second after stimulus onset. Again, and in contrast to face-selective nodes, DFC between object-selective electrodes and PFC was spottier and briefer. After regressing out the evoked response, these effects remained largely consistent (Supplementary Figure 50b).

In MEG, the results of the DFC analysis indicated a significant difference between the face-selective node and both PFC and V1/V2 ROIs within the initial 0.5-s time window (Supplementary Figure 50c). Similar to our findings in the task relevant condition, we observed a significant increase in content-specific connectivity in the low-frequency range (i.e., DFC for faces in the face-selective node), accompanied by a decrease in content-specific connectivity in the high-frequency range (i.e., DFC for objects in the face-selective node). In addition, smaller yet significant changes in DFC were observed between the object-selective node and both PFC and V1/V2 ROIs during the same time period (0-0.5 s), but this result was difficult to interpret due to the generalized decrease in DFC observed when object stimuli were presented (i.e., indicating stronger DFC for faces in the object-node connectivity).

When we conducted the DFC analysis after removing the evoked response, the significant effects in the low frequency bands persisted, while most of the high-frequency modulations disappeared (Supplementary Figure 50d). A significant modulation in DFC was observed between the face-selective node and both PFC and V1/V2 ROIs in the alpha band up to 0.5 s. Additionally, the transient low-frequency object-selective DFC that was found before with V1/V2 in object selective nodes disappeared once the evoked response was removed.

Overall, the combined analysis of task relevant and task irrelevant conditions in the MEG data replicated our main findings from task irrelevant trials, with a pronounced modulation of DFC between face-selective node and both PFC and V1/V2 in the low-frequency range in the first 0.5 s from stimulus presentation.

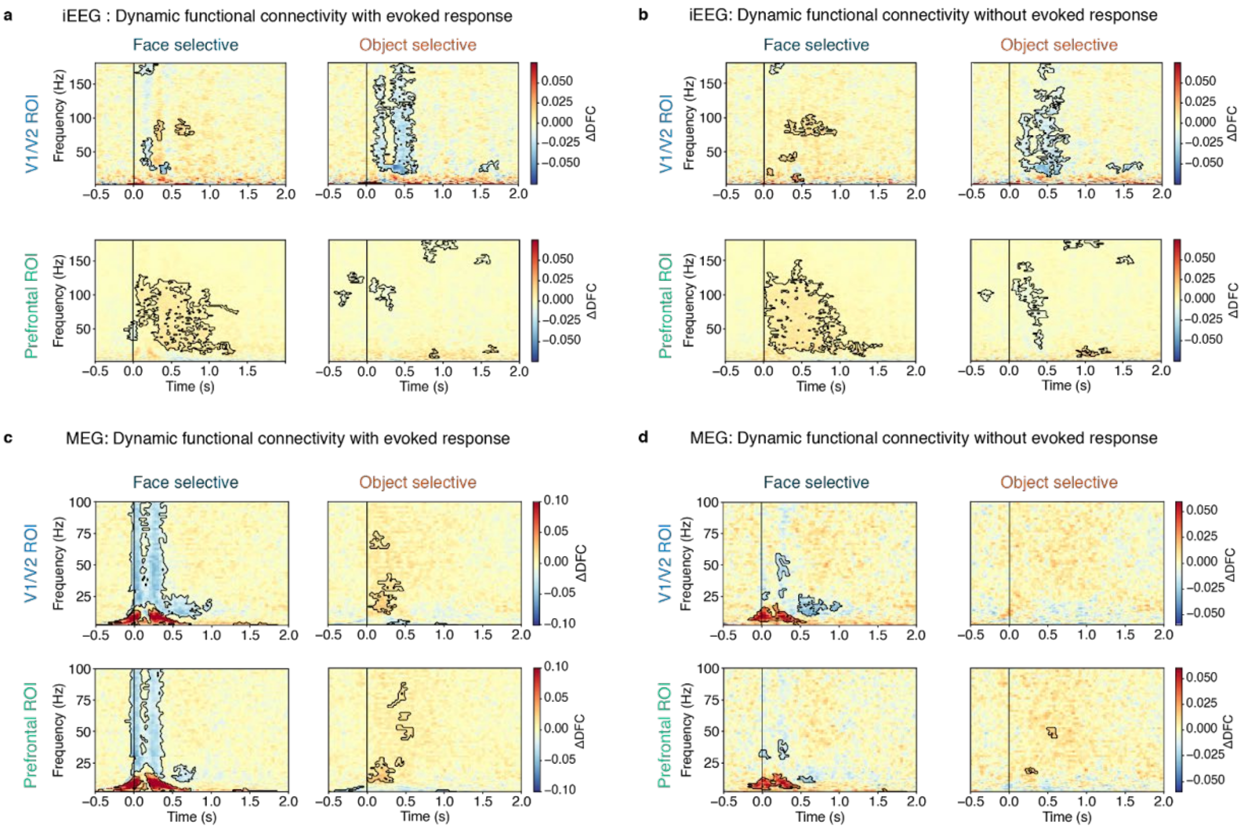

Supplementary Figure 50. Results of the DFC analysis (cluster based permutation tests, p<.05, two-tailed) on combined task relevant and task irrelevant trials before and after removing the evoked response on iEEG and MEG source data. **a-b**. iEEG results remained consistent with those performed on task irrelevant and task relevant, separately in V1/V2 regions face-selective: $N_{patients}=4$, $N_{electrodes}=30$, object-selective: $N_{patients}=4$, $N_{electrodes}=21$) and PFC (face-selective: $N_{patients}=19$, $N_{electrodes}=81$, object-selective: $N_{patients}=14$, $N_{electrodes}=57$). **c-d**. MEG results (N=65) were also consistent with those obtained from separate analyses on task irrelevant and task relevant data.

8. Putative Neural Correlates of (Visual) Consciousness (pNCC)

8.1. Pre-registered analyses

In addition to testing specific predictions of the theories, we also used this rich dataset for an exploratory analysis aimed at delineating cortical areas potentially participating in consciousness after excluding confounding factors related to cognitive/task-related processes[9]. The emphasis of this analysis was on ruling out areas whose presence relates to confounding

factors as opposed to visual consciousness per se. This test, while being excessively broad and thus not critical for the theories, nonetheless carries implications for both theories, considering their distinct predictions regarding the NCC. IIT predicts that the cortical substrate of consciousness should include posterior areas while agreeing that certain PFC areas should be excluded due to task confounds. GNWT predicts an involvement of PFC even after ruling out task-based effects (see methods section for analysis strategy).

We acknowledge that this analysis has limitations, e.g., it can overestimate areas participating in visual consciousness (as not all confounds are removed) and also underestimate it due to imperfect modelling of the haemodynamic response.

To delineate areas putatively involved in visual consciousness, we performed two types of analysis: a univariate and a multivariate analysis on the fMRI data only. Based on our preregistered predictions, we used a contrast-conjunction approach (see Methods), both on univariate activation and multivariate data.

First, in a univariate analysis, we identified voxels sensitive to the task itself, either to its goal, responding to the target, or to task relevance in general. Areas that are sensitive to task goal, were defined as those showing greater activity for task relevant targets vs. baseline, and no differential activity for non-targets vs. baseline (blank ITIs) (defined by the following contrast: [targets > bsl & task relevant = bsl & task irrelevant = bsl]). Areas that were sensitive to task-relevance, were defined as those expected to be responsive to all task relevant stimuli, but not to task irrelevant stimuli (defined by the following contrast: [targets > bsl & task relevant ≠ bsl & task irrelevant = bsl].

As expected, the two conjunction analyses designed to “rule out” areas responsive to task goals and task relevance identified several regions in the PFC ROIs (most prominently in inferior, middle and superior frontal gyrus, but also in cingulate cortex and others). Interestingly, these conjunctions also identified regions in posterior ROIs (e.g., inferior temporal gyrus, supramarginal gyrus and intraparietal sulcus) (Supplementary Figure 51). Areas that are sensitive to task goals and task relevance are listed in Supplementary Table 20. All of the voxels detected by these conjunctions were excluded from the reported putative NCCs.

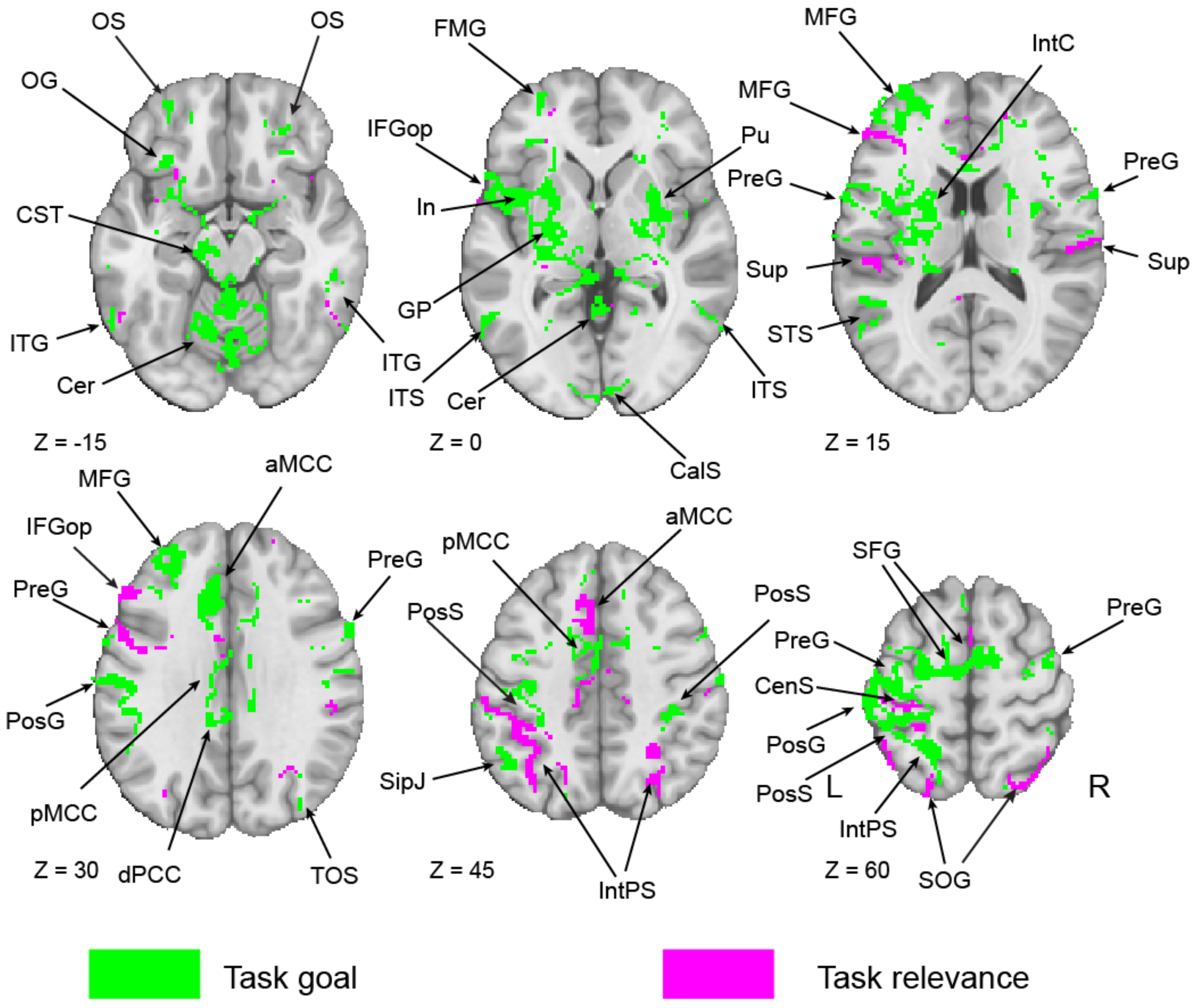

Supplementary Figure 51. Univariate fMRI contrast-conjunction analysis (see Methods) results identifying task goals (green) and task relevance (magenta) areas. Task goals areas were identified as: targets > bsl & task relevant = bsl & task irrelevant = bsl. Task relevance were identified as: targets > bsl & task relevant ≠ bsl & task irrelevant = bsl. The voxels identified as responsive to task goals or task relevance were subsequently removed from the results displayed in Supplementary Figure 53 and 54, as well as from Supplementary Tables 21 to 24. In all plots, axial brain slices are displayed from inferior (top left) to superior (bottom right), left and right hemisphere are displayed to the left and right respectively (N=73). Brain slices are from the Montreal Neurological Institute (MNI)152 template (MNI152NLin2009cAsym).

Neuroanatomical labels: ACC, Anterior Cingulate Gyrus; Accu, Nucleus Accumbens; AG, Angular Gyrus; aMCC, Middle-anterior part of the cingulate gyrus and sulcus; Amy, Amygdala; CalS, Calcarine Sulcus; Cau, Caudate Nucleus; CenS, Central Sulcus; Cer, Cerebellum; CST, Corticol Spinal Tract; Cu, Cuneus; dPCC, Posterior-dorsal part of the cingulate gyrus; FMG,

Fronto-Marginal Gyrus; Fu,Fusiform gyrus; GP, Globus Pallidus; Hipp, Hippocampus; IFGop, Opercular part of the Inferior Frontal Gyrus; IFGtri, Triangular part of the Inferior Frontal Gyrus; In, Insula; IntC, Internal Capsule; IntPS, Intraparietal Sulcus; IOG, Inferior Occipital Gyrus; ITG, Inferior Temporal Gyrus; ITS, Inferior Temporal Sulcus; Li, Lingual Gyrus; LOS, Lateral Orbital Sulcus; MFG, Middle Frontal Gyrus; MOG, Middle Occipital Gyrus; MTG, Middle Temporal Gyrus; OG, Orbital Gyrus; OP, Occipital Pole; OS, Orbital Sulci; PDC, Posterior Dorsal Cingulate; pMCC, Middle-posterior part of the cingulate gyrus and sulcus; PosG, Postcentral Gyrus; PosS, Postcentral sulcus; PP, Planum polare of the superior temporal gyrus; preCu, Precuneus; PreG, Precentral Gyrus; PreSinf, Inferior part of the Precentral Sulcus; PT, Planum Temporale of the Superior Temporal Gyrus; Pu, Putamen; SFG, Superior Frontal Gyrus; SipJ, Sulcus intermedius primus of Jensen; SOG, Superior Occipital Gyrus; STGL, Lateral aspect of the superior temporal gyrus; STS, Superior Temporal Sulcus; SupG, Supramarginal gyru; Thal, Thalamus; TOS, Transverse Occipital Sulcus.

Anatomical ROIs (Destrieux atlas)	Task goals		Task relevance	
Short name	n voxels	% voxels	n voxels	% voxels
**Posterior ROIs**				
G_cuneus	33	1,32	0	0,00
G_occipital_middle	20	0,81	4	0,16
G_occipital_sup	3	0,15	0	0,00
G_oc-temp_med-Lingual	69	2,30	0	0,00
G_oc-temp_med-Parahip	30	2,13	0	0,00
G_pariet_inf-Angular[b]	77	2,21	109	3,13
G_pariet_inf-Supramar[b]	219	5,07	237	5,49
G_temp_sup-Lateral	12	0,32	3	0,08
G_temp_sup-Plan_tempo	0	0,00	9	0,54
G_temporal_inf	93	6,42	42	2,90
G_temporal_middle	100	2,80	4	0,11
Pole_occipital	16	0,66	0	0,00
S_calcarine	55	2,27	0	0,00
S_interm_prim-Jensen[b]	93	11,58	0	0,00
S_intrapariet_and_P_trans	90	2,37	630	16,61
S_oc_middle_and_Lunatus	3	0,30	0	0,00
S_oc_sup_and_transversal	5	0,35	19	1,34
S_occipital_ant[b]	18	2,46	0	0,00
S_oc-temp_lat[b]	7	0,60	10	0,86
S_temporal_inf[b]	73	3,92	12	0,64
S_temporal_sup	168	3,40	7	0,14
**PFC ROIs**				
G_and_S_cingul-Ant[d]	109	3,28	25	0,75
G_and_S_cingul-Mid-Ant[d]	374	18,99	141	7,16
G_and_S_cingul-Mid-Post[a]	301	15,65	86	4,47
G_and_S_frontomargin[cd]	119	15,18	6	0,77
G_front_inf-Opercular[d]	584	26,14	19	0,85
G_front_inf-Orbital[d]	16	2,57	0	0,00

G_front_inf-Triangul[d]	19	1,15	9	0,55
G_front_middle[d]	375	6,10	110	1,79
G_front_sup[d]	1033	9,92	154	1,48
G_orbital[bc]	13	0,86	28	1,84
G_precentral[abc]	725	17,29	27	0,64
G_subcallosal[cd]	22	3,20	1	0,15
Lat_Fis-ant-Horizont	24	4,38	0	0,00
Lat_Fis-ant-Vertical	27	5,86	0	0,00
S_front_inf[b]	161	7,75	61	2,94
S_front_middle[d]	378	17,42	1	0,05
S_front_sup[d]	102	2,73	3	0,08
S_orbital_lateral[cd]	10	1,88	0	0,00
S_orbital_med-olfact[cd]	11	2,46	0	0,00
S_orbital-H_Shaped[cd]	108	8,02	0	0,00
S_precentral-inf-part[abc]	82	3,71	114	5,16
S_suborbital[cd]	4	0,15	0	0,00

[a]Strictly speaking, frontal but not part of the PFC

[b]Areas in which IIT considers that due to their cytoarchitectonic composition, effects might be found, regardless of their location

[c]Areas not indicated as relevant by the GNWT proponents

[d]Regions where an effect found would pose a challenge for IIT

Supplementary Table 20. Results from the univariate fMRI contrast-conjunction pNCC analysis, describing areas responsive to task goals and task relevance. For each of the predefined anatomical ROIs, we count the number of voxels showing activation, and calculate their proportion with respect to the total number of voxels of the ROI.

8.1.1 Univariate pNCC analysis

After having identified and removed areas sensitive to the task itself, among the remaining areas, we identified in a univariate analysis, brain areas sensitive to changes in the content of consciousness, so that they consistently respond to at least one stimulus category (Stimulus>Baseline) in both the task relevant and task irrelevant conditions. The contrast conjunction used to identify visually-responsive cortical areas was defined as: [(task relevant stimulus > baseline) & (task irrelevant > baseline)] OR [(task relevant stimulus < baseline) & (task irrelevant < baseline)]. To compute the above conjunctions, we first created thresholded individual z maps by contrasting the presence of stimuli vs. baseline (corrected for multiple comparisons, using gaussian random-field cluster thresholding, with a cluster formation threshold of one-sided $p<0.001$ ($z \geq 3.1$,) and a cluster significance threshold of $p<0.05$, two-tailed) in the task relevant and irrelevant conditions separately. These maps depicting the individual contrast are presented in Supplementary Figure 52. As can be seen, besides effects located in the visual cortex and other posterior regions, the presentation of stimuli elicits both activations and deactivations in several prefrontal areas for both task conditions.

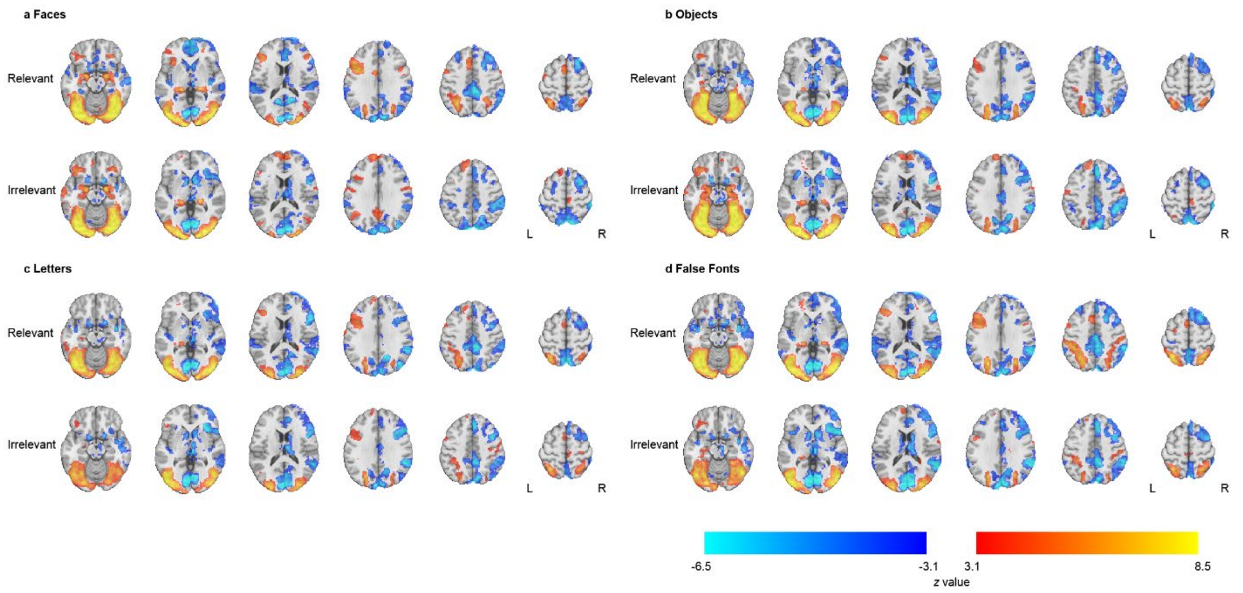

Supplementary Figure 52. Contrast of parameter estimates (stimulus vs. baseline) z maps (N=73) used in the conjunction that identifies the putative NCCs (Supplementary Figure 53). Here we show z maps for each stimulus category (**a**. faces; **b**. objects; **c**. letters; **d**. false fonts) and condition (top, Relevant; bottom, Irrelevant). Brain slices are from the Montreal Neurological Institute (MNI)152 template (MNI152NLin2009cAsym).

The main results of the univariate contrast conjunction analysis identifying visually-responsive cortical areas, after removing (confounding) task-responsive cortical regions are shown in Supplementary Figure 53. This conjunction analysis identified in posterior cortex, several regions in ventral occipito-temporal regions showing consistent task-independent activation for three or all four stimulus categories. In PFC, inferior and middle frontal gyrus and orbital cortex were activated for at least one of the stimulus categories. A number of areas showed deactivations both in posterior cortex (e.g., striate and some extrastriate areas) and PFC (e.g., inferior and middle frontal gyrus and orbital cortex). Supplementary Tables 21 and 22 present the count of activated and deactivated voxels detected in the analysis separately per theory defined anatomical ROIs.

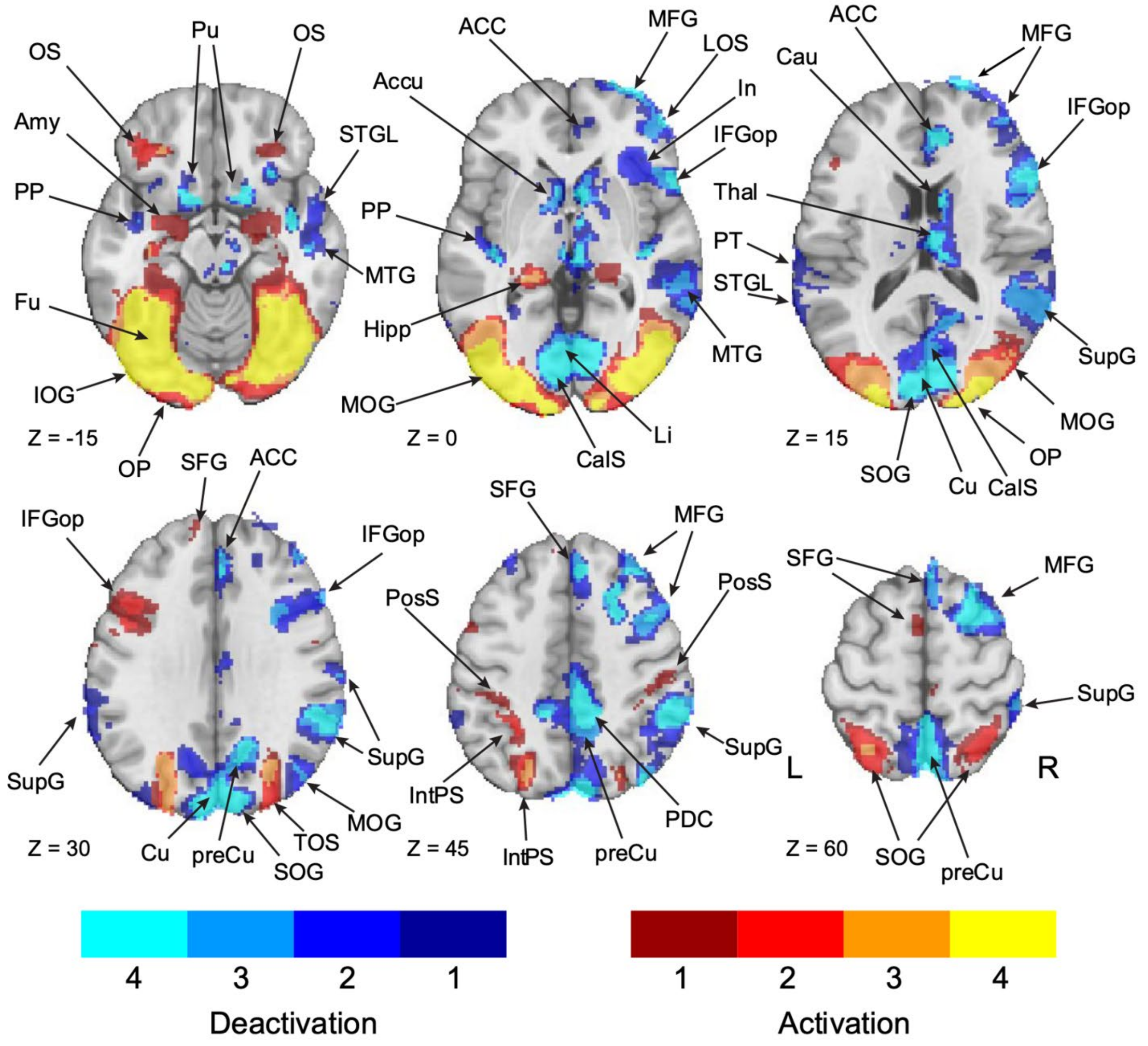

Supplementary Figure 53. fMRI Univariate contrast conjunction analysis (see Methods) identifying visually-responsive cortical areas, after removing (confounding) task-responsive cortical regions (N=73). Axial brain slices show activations (reds-yellows) and deactivations (blues), relative to a blank-screen baseline condition for each of the 4 stimulus categories. Color scales indicate the number of stimulus categories (1-4) passing the contrast-conjunction, as in [(task relevant stimulus > baseline) & (task irrelevant > baseline)] OR [(task relevant stimulus < baseline) & (task irrelevant < baseline)]. Brain slices are from the Montreal Neurological Institute (MNI)152 template (MNI152NLin2009cAsym).

Anatomical ROIs (Destrieux atlas)	Face		Object		Letter		False Font	
	n voxels	% voxels	n voxels	% voxels	n voxels	% voxels	n voxels	% voxels
**Posterior ROI**								
G_and_S_occipital_inf	1897	94,57	1934	96,41	1717	85,59	1798	89,63
G_cuneus	100	3,99	60	2,39	38	1,52	21	0,84
G_occipital_middle	797	32,37	1379	56,01	1045	42,45	1246	50,61
G_occipital_sup	71	3,62	299	15,23	233	11,87	369	18,80
G_oc-temp_lat-fusifor	2144	82,91	2306	89,17	1491	57,66	1838	71,08
G_oc-temp_med-Lingual	377	12,56	520	17,32	326	10,86	309	10,29
G_oc-temp_med-Parahip	168	11,94	321	22,81	0	0,00	36	2,56
G_pariet_inf-Angular[b]	0	0,00	15	0,43	71	2,04	56	1,61
G_pariet_inf-Supramar[b]	0	0,00	0	0,00	1	0,02	58	1,34
G_temporal_inf	83	5,73	296	20,44	337	23,27	472	32,60
G_temporal_middle[b]	2	0,06	5	0,14	7	0,20	19	0,53
Pole_occipital	2105	87,34	2039	84,61	1352	56,10	1278	53,03
S_calcarine	58	2,39	67	2,76	25	1,03	30	1,24
S_intrapariet_and_P_trans	0	0,00	243	6,41	769	20,27	1107	29,19
S_oc_middle_and_Lunatus	646	63,96	931	92,18	832	82,38	889	88,02
S_oc_sup_and_transversal	22	1,55	1118	79,01	807	57,03	1025	72,44
S_occipital_ant[b]	270	36,83	433	59,07	468	63,85	516	70,40
S_oc-temp_lat[b]	682	58,79	820	70,69	781	67,33	876	75,52
S_temporal_inf[b]	23	1,24	97	5,21	101	5,43	146	7,85
S_temporal_sup	13	0,26	3	0,06	1	0,02	4	0,08
**PCF ROI**								
G_front_inf-Opercular[d]	43	1,92	0	0,00	66	2,95	0	0,00
G_front_inf-Orbital[d]	4	0,64	0	0,00	0	0,00	0	0,00
G_front_inf-Triangul[d]	8	0,49	0	0,00	20	1,21	0	0,00
G_front_middle[d]	19	0,31	0	0,00	71	1,16	0	0,00
G_front_sup[d]	21	0,20	11	0,11	123	1,18	1	0,01
G_orbital[bc]	74	4,87	39	2,57	0	0,00	0	0,00
G_precentral[abc]	87	2,07	30	0,72	77	1,84	59	1,41
G_subcallosal[cd]	31	4,51	0	0,00	0	0,00	0	0,00
Lat_Fis-ant-Horizont	0	0,00	5	0,91	0	0,00	0	0,00
S_front_inf[b]	167	8,04	0	0,00	209	10,06	1	0,05
S_front_sup[d]	0	0,00	0	0,00	8	0,21	0	0,00
S_orbital_lateral[cd]	7	1,32	0	0,00	0	0,00	0	0,00
S_orbital_med-olfact[cd]	4	0,89	0	0,00	0	0,00	0	0,00
S_orbital-H_Shaped[cd]	242	17,97	129	9,58	0	0,00	16	1,19
S_precentral-inf-part[abc]	0	0,00	0	0,00	235	10,63	136	6,15

[a]Strictly speaking, frontal but not part of the PFC

[b]Areas in which IIT considers that due to their cytoarchitectonic composition, effects might be found, regardless of their location

[c]Areas not indicated as relevant by the GNWT proponents

[d]Regions where an effect found would pose a challenge for IIT

Supplementary Table 21. Results from the univariate fMRI contrast-conjunction pNCC analysis, aimed at "ruling in" putative NCCs, reporting *activation* in response to the presentation of stimuli, regardless of their relevance. For each of the predefined anatomical ROIs, we count the number of

voxels showing activation, and calculate their proportion with respect to the total number of voxels of the ROI.

Anatomical ROIs (Destrieux atlas)	Face		Object		Letter		False Font	
	n voxels	% voxels	n voxels	% voxels	n voxels	% voxels	n voxels	% voxels
**Posterior ROI**								
G_cuneus	954	38,05	1609	64,18	1589	63,38	1474	58,80
G_occipital_middle	258	10,48	81	3,29	203	8,25	180	7,31
G_occipital_sup	676	34,44	601	30,62	523	26,64	586	29,85
G_oc-temp_lat-fusifor	1	0,04	0	0,00	0	0,00	0	0,00
G_oc-temp_med-Lingual	727	24,22	1010	33,64	1024	34,11	1194	39,77
G_oc-temp_med-Parahip	0	0,00	0	0,00	6	0,43	0	0,00
G_pariet_inf-Angular[b]	293	8,40	740	21,22	509	14,60	806	23,11
G_pariet_inf-Supramar[b]	706	16,34	629	14,56	556	12,87	900	20,83
G_temp_sup-Lateral[b]	42	1,11	31	0,82	198	5,21	355	9,35
G_temp_sup-Plan_tempo[b]	273	16,27	79	4,71	45	2,68	357	21,28
G_temporal_inf	0	0,00	13	0,90	6	0,41	0	0,00
G_temporal_middle[b]	23	0,64	272	7,61	408	11,41	592	16,55
Pole_occipital	1	0,04	1	0,04	0	0,00	2	0,08
S_calcarine	406	16,74	671	27,66	739	30,46	570	23,50
S_interm_prim-Jensen[b]	335	41,72	437	54,42	416	51,81	359	44,71
S_intrapariet_and_P_trans	65	1,71	235	6,20	154	4,06	6	0,16
S_oc_middle_and_Lunatus	0	0,00	0	0,00	1	0,10	0	0,00
S_oc_sup_and_transversal	91	6,43	62	4,38	43	3,04	60	4,24
S_temporal_inf[b]	0	0,00	14	0,75	55	2,96	9	0,48
S_temporal_sup	197	3,99	878	17,78	911	18,45	1526	30,90
**PCF ROI**								
G_and_S_cingul-Ant[d]	313	9,41	339	10,19	359	10,79	376	11,30
G_and_S_cingul-Mid-Ant[d]	3	0,15	130	6,60	93	4,72	34	1,73
G_and_S_cingul-Mid-Post[a]	47	2,44	74	3,85	73	3,80	182	9,46
G_and_S_frontomargin[cd]	29	3,70	81	10,33	44	5,61	61	7,78
G_and_S_transv_frontopol[cd]	79	7,66	118	11,45	134	13,00	198	19,20
G_front_inf-Opercular[d]	183	8,19	562	25,16	573	25,65	427	19,11
G_front_inf-Orbital[d]	3	0,48	56	9,00	29	4,66	46	7,40
G_front_inf-Triangul[d]	37	2,24	280	16,98	398	24,14	292	17,71
G_front_middle[d]	414	6,74	1105	17,98	956	15,55	1409	22,93
G_front_sup[d]	281	2,70	746	7,16	666	6,39	929	8,92
G_orbital[bc]	28	1,84	92	6,06	80	5,27	101	6,65
G_precentral[abc]	6	0,14	111	2,65	89	2,12	41	0,98
G_subcallosal[cd]	111	16,16	44	6,40	64	9,32	97	14,12
Lat_Fis-ant-Horizont	0	0,00	200	36,50	223	40,69	79	14,42
Lat_Fis-ant-Vertical	79	17,14	179	38,83	206	44,69	192	41,65
S_front_inf[b]	62	2,99	314	15,12	428	20,61	193	9,29
S_front_middle[d]	9	0,41	64	2,95	29	1,34	91	4,19
S_front_sup[d]	503	13,46	581	15,55	463	12,39	693	18,54
S_orbital_lateral[cd]	0	0,00	88	16,57	88	16,57	130	24,48

S_orbital_med-olfact[cd]	88	19,69	56	12,53	83	18,57	54	12,08
S_orbital-H_Shaped[cd]	1	0,07	0	0,00	0	0,00	0	0,00
S_precentral-inf-part[abc]	117	5,29	698	31,57	668	30,21	344	15,56
S_suborbital[cd]	2	0,28	0	0,00	0	0,00	0	0,00

[a]Strictly speaking, frontal but not part of the PFC

[b]Areas in which IIT considers that due to their cytoarchitectonic composition, effects might be found, regardless of their location

[c]Areas not indicated as relevant by the GNWT proponents

[d]Regions where an effect found would pose a challenge for IIT

Supplementary Table 22. Results of the univariate fMRI contrast-conjunction pNCC analysis for voxels showing *deactivation* rather than activation (presented in Supplementary Table 21).

8.1.2 Univariate Participant-level pNCC analysis

The preregistered univariate pNCC analysis was aimed at testing the theories' predictions about the areas potentially subserving conscious processing. A critical point of disagreement here pertains to prefrontal areas, where GNWT predicts they should be included in the resulting pNCCs, while IIT claims otherwise. However, the global workspace is held to be widely distributed in prefrontal and parietal areas[10], in a manner that might be idiosyncratic to a specific participant. Thus, a group level analysis might fail to detect prefrontal pNCCs even if they do exist. To account for this possibility, we complemented our analysis with an additional univariate pNCC analysis, performed at the participant-level. Supplementary Table 23 shows the proportion of participants that passed the conjunction analysis for each stimulus category in each a priori defined anatomical ROI.

As the results show, besides the majority of participants showing activations in posterior ROIs, there is some agreement also around activations in prefrontal regions. For example, more than 10% of participants showed activations in superior frontal gyrus across all stimulus categories. Similar results were also found in inferior and middle frontal gyri. Interestingly, these areas showed a considerable proportion of participants also showing deactivations, e.g., in superior and middle frontal gyrus, more than 30% of participants show deactivations in all stimulus categories, and 24% participants showing the same in inferior frontal gyrus.

It should be noted that the conjunction analyses used here implement a logical AND operation between two maps that were corrected for multiple comparisons to have an alpha level of 0.05. Therefore, the conjunction test is conservative, as assuming independence of the maps, the alpha level of the conjunction is 0.0025.

Anatomical ROIs (Destrieux atlas)	Activation (% participants)				Deactivation (% participants)			
	Face	Object	Letter	False Font	Face	Object	Letter	False Font
**Posterior ROI**								
G_and_S_occipital_inf	94,52	94,52	89,04	94,52	1,37	4,11	6,85	6,85
G_cuneus	83,56	72,60	56,16	46,58	72,60	75,34	56,16	68,49
G_occipital_middle	94,52	94,52	91,78	93,15	41,10	17,81	23,29	27,40
G_occipital_sup	89,04	90,41	86,30	87,67	71,23	65,75	52,05	64,38
G_oc-temp_lat-fusifor	94,52	91,78	68,49	89,04	8,22	5,48	9,59	6,85

G_oc-temp_med-Lingual	94,52	94,52	83,56	83,56	64,38	64,38	49,32	50,68
G_oc-temp_med-Parahip	53,42	68,49	2,74	12,33	1,37	0,00	2,74	0,00
G_pariet_inf-Angular[b]	39,73	30,14	35,62	50,68	39,73	35,62	36,99	43,84
G_pariet_inf-Supramar[b]	9,59	8,22	12,33	30,14	30,14	30,14	27,40	38,36
G_temp_sup-Lateral[b]	17,81	2,74	5,48	2,74	23,29	28,77	28,77	35,62
G_temp_sup-Plan_tempo[b]	4,11	4,11	4,11	1,37	17,81	24,66	17,81	19,18
G_temporal_inf	71,23	60,27	54,79	79,45	17,81	13,70	12,33	15,07
G_temporal_middle[b]	53,42	32,88	38,36	58,90	35,62	26,03	34,25	38,36
Pole_occipital	94,52	94,52	91,78	94,52	47,95	49,32	32,88	46,58
Pole_temporal	9,59	0,00	2,74	1,37	1,37	1,37	2,74	2,74
S_calcarine	84,93	89,04	63,01	57,53	65,75	69,86	45,21	54,79
S_interm_prim-Jensen[b]	1,37	1,37	2,74	5,48	23,29	21,92	20,55	23,29
S_intrapariet_and_P_trans	32,88	36,99	53,42	69,86	30,14	24,66	20,55	13,70
S_oc_middle_and_Lunatus	93,15	93,15	91,78	93,15	8,22	1,37	5,48	6,85
S_oc_sup_and_transversal	73,97	91,78	82,19	91,78	46,58	30,14	21,92	35,62
S_occipital_ant[b]	84,93	86,30	78,08	94,52	5,48	4,11	2,74	6,85
S_oc-temp_lat[b]	91,78	86,30	67,12	89,04	1,37	2,74	2,74	2,74
S_temporal_inf[b]	60,27	64,38	60,27	82,19	15,07	13,70	17,81	15,07
S_temporal_sup	52,05	24,66	20,55	26,03	32,88	32,88	32,88	41,10
**PCF ROI**								
G_and_S_cingul-Ant[d]	5,48	2,74	4,11	5,48	20,55	19,18	12,33	16,44
G_and_S_cingul-Mid-Ant[d]	1,37	1,37	4,11	2,74	8,22	15,07	9,59	6,85
G_and_S_cingul-Mid-Post[a]	0,00	0,00	0,00	0,00	8,22	10,96	12,33	8,22
G_and_S_frontomargin[cd]	8,22	2,74	4,11	2,74	10,96	16,44	9,59	17,81
G_and_S_transv_frontopol[cd]	6,85	2,74	5,48	4,11	24,66	16,44	12,33	17,81
G_front_inf-Opercular[d]	23,29	9,59	13,70	12,33	24,66	38,36	31,51	32,88
G_front_inf-Orbital[d]	12,33	6,85	4,11	2,74	13,70	17,81	15,07	16,44
G_front_inf-Triangul[d]	17,81	6,85	12,33	12,33	24,66	26,03	28,77	32,88
G_front_middle[d]	28,77	13,70	19,18	17,81	34,25	39,73	36,99	34,25
G_front_sup[d]	15,07	10,96	16,44	12,33	36,99	41,10	31,51	36,99
G_orbital[bc]	21,92	8,22	4,11	1,37	17,81	19,18	15,07	17,81
G_precentral[abc]	26,03	15,07	13,70	21,92	15,07	21,92	24,66	23,29
G_rectus[cd]	8,22	1,37	2,74	0,00	0,00	0,00	1,37	2,74
G_subcallosal[cd]	4,11	1,37	0,00	0,00	4,11	8,22	2,74	5,48
Lat_Fis-ant-Horizont	4,11	5,48	5,48	2,74	10,96	16,44	19,18	12,33
Lat_Fis-ant-Vertical	1,37	4,11	4,11	1,37	16,44	21,92	21,92	17,81
S_front_inf[b]	27,40	13,70	21,92	17,81	23,29	31,51	31,51	27,40
S_front_middle[d]	6,85	4,11	6,85	2,74	19,18	19,18	13,70	21,92
S_front_sup[d]	12,33	6,85	4,11	4,11	24,66	31,51	23,29	24,66
S_orbital_lateral[cd]	6,85	5,48	4,11	4,11	13,70	12,33	12,33	16,44
S_orbital_med-olfact[cd]	9,59	0,00	0,00	0,00	5,48	5,48	1,37	4,11
S_orbital-H_Shaped[cd]	23,29	10,96	2,74	4,11	5,48	8,22	6,85	5,48
S_precentral-inf-part[abc]	26,03	9,59	23,29	19,18	23,29	34,25	31,51	27,40
S_suborbital[cd]	12,33	1,37	1,37	1,37	2,74	0,00	2,74	5,48

[a]Strictly speaking, frontal but not part of the PFC

[b]Areas in which IIT considers that due to their cytoarchitectonic composition, effects might be found, regardless of their location

[c]Areas not indicated as relevant by the GNWT proponents

[d]Regions where an effect found would pose a challenge for IIT

Supplementary Table 23. Results of the univariate fMRI contrast-conjunction pNCC analysis, performed at the participant-level.

8.1.3 Multivariate pNCC analysis

We also performed a fMRI multivariate contrast-conjunction analysis identifying areas showing consistent whole-brain searchlight decoding of stimulus vs. baseline using thresholded statistical maps obtained at the participant-level. Conjunction was defined as above chance decoding both for task relevant & task irrelevant stimuli for each stimulus category separately. The pNCC analysis taking a multivariate approach revealed regions in extrastriate and early visual cortex and small, right lateralized clusters in PFC (Supplementary Figure 54). See Supplementary Table 24 for number of voxels per anatomical region. Brain slices are from the Montreal Neurological Institute (MNI)152 template (MNI152NLin2009cAsym).

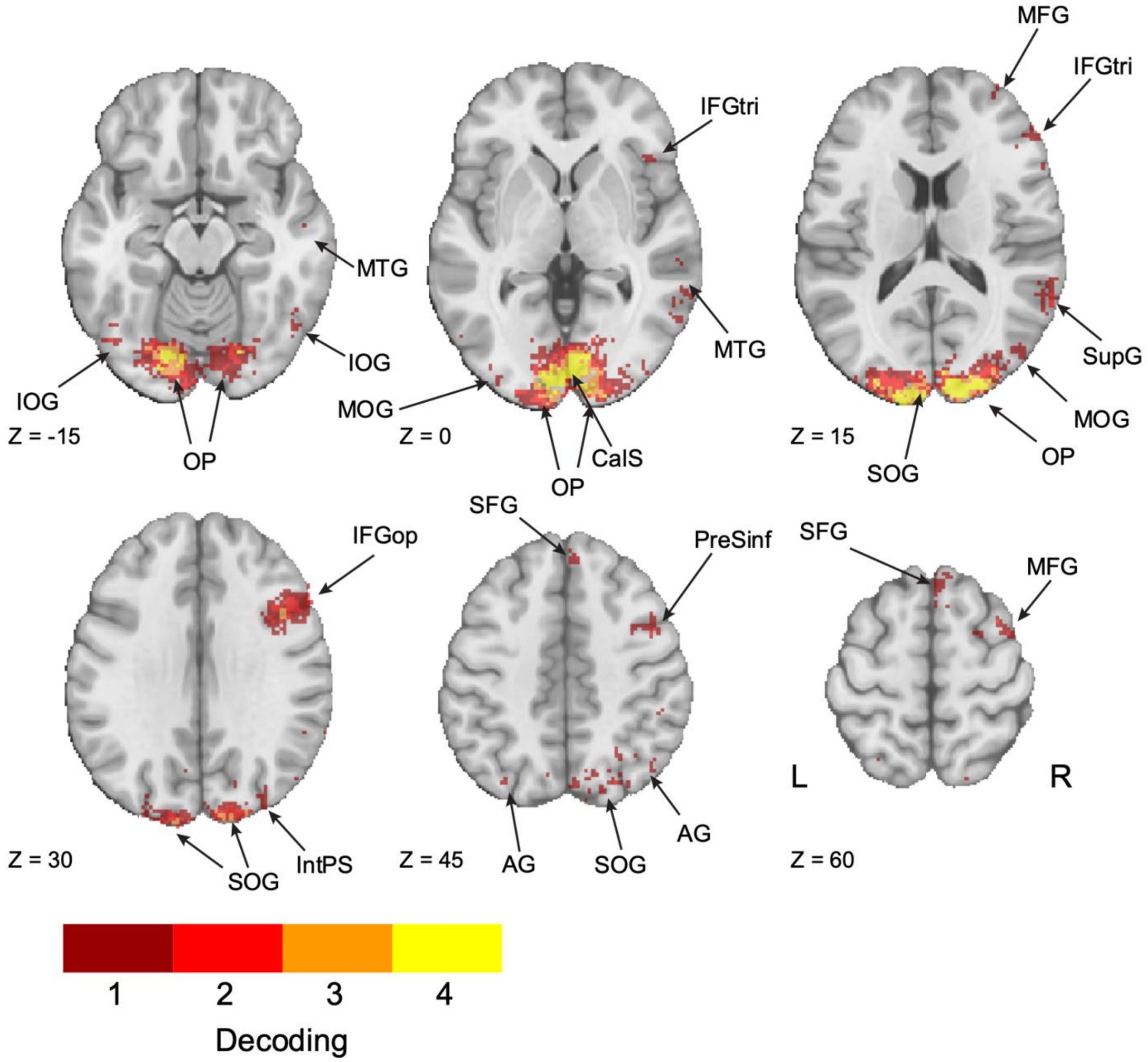

Supplementary Figure 54. fMRI Multivariate contrast conjunction analysis (see Methods) identifying visually-responsive cortical areas, after removing (confounding) task-responsive cortical regions (N=73). Colorbar shows the number of stimulus categories passing the conjunction.

Anatomical ROIs (Destrieux atlas)	Face		Object		Letter		False Font	
	n voxels	% voxels	n voxels	% voxels	n voxels	% voxels	n voxels	% voxels
**Posterior ROI**								
G_and_S_occipital_inf	123	6,13	71	3,54	18	0,90	118	5,88
G_cuneus	429	17,11	346	13,80	433	17,27	355	14,16

G_occipital_middle	128	5,20	109	4,43	87	3,53	213	8,65
G_occipital_sup	598	30,46	563	28,68	397	20,22	512	26,08
G_oc-temp_lat-fusifor	104	4,02	19	0,73	15	0,58	17	0,66
G_oc-temp_med-Lingual	878	29,25	560	18,65	524	17,46	580	19,32
G_pariet_inf-Angular[b]	0	0,00	36	1,03	35	1,00	68	1,95
G_pariet_inf-Supramar[b]	0	0,00	15	0,35	20	0,46	55	1,27
G_temp_sup-Lateral[b]	0	0,00	0	0,00	0	0,00	24	0,63
G_temporal_inf	1	0,07	2	0,14	6	0,41	51	3,52
G_temporal_middle[b]	0	0,00	2	0,06	52	1,45	15	0,42
Pole_occipital	1085	45,02	656	27,22	374	15,52	391	16,22
S_calcarine	310	12,78	298	12,28	287	11,83	241	9,93
S_interm_prim-Jensen[b]	0	0,00	3	0,37	35	4,36	2	0,25
S_intrapariet_and_P_trans	0	0,00	12	0,32	64	1,69	32	0,84
S_oc_middle_and_Lunatus	139	13,76	108	10,69	44	4,36	107	10,59
S_oc_sup_and_transversal	175	12,37	192	13,57	149	10,53	341	24,10
S_occipital_ant[b]	3	0,41	0	0,00	1	0,14	9	1,23
S_oc-temp_lat[b]	0	0,00	0	0,00	0	0,00	29	2,50
S_temporal_inf[b]	0	0,00	0	0,00	11	0,59	23	1,24
S_temporal_sup	2	0,04	29	0,59	39	0,79	40	0,81
**PFC ROI**								
G_and_S_cingul-Ant[d]	0	0,00	0	0,00	6	0,18	0	0,00
G_front_inf-Opercular[d]	1	0,04	66	2,95	114	5,10	96	4,30
G_front_inf-Triangul[d]	2	0,12	4	0,24	43	2,61	64	3,88
G_front_middle[d]	1	0,02	27	0,44	82	1,33	37	0,60
G_front_sup[d]	19	0,18	0	0,00	64	0,61	0	0,00
G_precentral[abc]	0	0,00	11	0,26	37	0,88	50	1,19
Lat_Fis-ant-Horizont	0	0,00	15	2,74	0	0,00	0	0,00
Lat_Fis-ant-Vertical	0	0,00	3	0,65	0	0,00	0	0,00
S_front_inf[b]	0	0,00	17	0,82	136	6,55	17	0,82
S_front_middle[d]	0	0,00	0	0,00	2	0,09	0	0,00
S_front_sup[d]	0	0,00	0	0,00	3	0,08	0	0,00
S_precentral-inf-part[abc]	0	0,00	49	2,22	235	10,63	174	7,87

[a]Strictly speaking, frontal but not part of the PFC

[b]Areas in which IIT considers that due to their cytoarchitectonic composition, effects might be found, regardless of their location

[c]Areas not indicated as relevant by the GNWT proponents

[d]Regions where an effect found would pose a challenge for IIT

Supplementary Table 24. Results of the multivariate fMRI conjunction pNCC analysis. The same conventions from Supplementary Table 20 are used here.

8.1.4 Summary pNCC analysis

Together, the pNCC analysis revealed a pattern of candidate areas that was more spatially restricted than anticipated by the rather extensive preregistered theory ROIs. Specifically, the MFG, IFG and orbital cortex might participate in consciousness, as predicted by GNWT. Furthermore, the scant activation patterns found in PFC compared to the widespread deactivations

was surprising, and suggests a reconsideration of the strong focus on activations (relative to deactivations) when assessing this region's role in conscious perception. However, we consider this analysis an informative yet liberal test, given its potential to overestimate candidate cortical areas for consciousness by including non-conscious sensory precursors.

Overall, the pNCC analysis highlighted portions of PFC as potentially relevant for consciousness. While posterior cortex showed the most consistent activation and decodability of content, IIT must account for the responsiveness of the MFG and IFG, which were not ruled out as task-related despite the minimized cognitive processing of task-irrelevant stimuli[11].

The highly localized decoding of conscious content in PFC, along with restricted activations and deactivations in the pNCC analysis, supports a "localized spark" rather than the "widespread ignition" predicted by GNWT, presenting a significant challenge to the theory[12] .

9. Participants

9.1 iEEG demographics

Below we describe the characteristics of the iEEG patients (Supplementary Table 25). Also, we provide here further details about the three patients whose behavior fell short of the predefined behavioral criteria (i.e. hits < 70%, FA > 30%), but were nonetheless included in the analysis: one of them kept the response button pressed for most of the time during experiment, the other's low performance was driven by one of the categories only (which the patient reported having difficulty to detect), and the third's performance was very close to the threshold (65%) and had very low FA rate (2%).

Particip ant ID	Sex	Age [years]	Handed-ness	Electrode Scheme	Number of Implanted Electrodes	Implant hemisphere	IQ [value, test]	WADA	Seizu re Type	Age of Onset	Native Language
SE103	F	49	L	stereo	58	B	109, FSIQ	N/A	N/A	27	English
SE106	F	18	R	stereo	118	L	>70, FSIQ	N/A	N/A	12	English
SE107	M	24	R	stereo	168	L	>70, FSIQ	N/A	N/A	13	English
SE108	F	16	R	stereo	108	L	>70, FSIQ	N/A	N/A	12	English
SE109	F	50	R	stereo	104	B	>70, FSIQ	N/A	N/A	45	English
SE110	F	15	R	stereo	186	R	>70, FSIQ	N/A	N/A	10	English
SE112	F	17	R	stereo	158	R	>70, FSIQ	N/A	N/A	7	English

SE113	F	26	A	stereo	60	B	>70, FSIQ	N/A	N/A	19	English
SE115	M	17	R	stereo	88	R	>70, FSIQ	N/A	N/A	6	English
SE118	M	11	R	stereo	164	L	>70, FSIQ	N/A	N/A	8	English
SE119	M	29	R	stereo	104	B	>70, FSIQ	N/A	N/A	28	Polish
SE120	M	12	L	stereo	164	L	>70, FSIQ	N/A	N/A	1	English
SF102	F	30	R	subdural grid & strips, depths	133	L	98, VCI; 90, POI; 83, WMI; 100, PSI	L	FBTC	22	English
SF103	M	24	R	subdural grid & strips, depths	189	L	145, VCI; 96, POI; 95, WMI; 86, PSI	predominantly L, mild R contribution	FA	11	English
SF104	F	23	R	subdural grid & strips, depths	116	L	79, VCI; 62, POI	L	FBTC	13	English
SF105	M	31	R	subdural grid & strips, depths	176	L	116, VCI; 111, POI; 102, WMI; 114, PSI	L	FBTC	22	English
SF106	M	17	R	subdural grid & strips, depths	156	L	N/A	N/A	FM	11	English
SF107	F	31	R	subdural grid & strips, depths	242	R	104, VCI	N/A	FIA	23	English
SF109	F	30	L	subdural grid & strips, depths	102	R	107, VCI; 86, POI; 95, WMI; 92, PSI	predominantly L, mild R contribution	FIA	3	English
SF110	F	17	R	subdural grid & strips, depths	174	L	89, VCI; 100, POI; 83, WMI; 70, PSI	N/A	FIA, FBTC	2	English
SF112	M	23	R	subdural strips, depths	180	B	107, VCI; 123, POI; 131, WMI; 100, PSI	L	FIA	19	English

SF113	F	38	R(converted from L)	subdural grid & strips, depths	132	R	100, VCI; 102, POI; 114, WMI; 102, PSI	L	FA, FIA	34	English
SF116	M	43	L	stereo	166	B	144, VCI	N/A	FIA	38	English
SF117	M	28	R	stereo	174	B	105, VCI; 107, POI; 95, WMI; 108, PSI	N/A	FBTC, FIA	26	English
SF119	M	37	R	subdural grid & strips, depths	104	L	102, VCI; 98, POI; 97, WMI; 108, PSI	L	FBTC	36	English
SF120	F	61	R	stereo	79	L	N/A	N/A	FA, FIA	44	English
SF121	F	50	R	stereo	75	R	83, VCI; 77, POI; 77, WMI; 76, PSI	L	FIA	1	English
SF122	F	27	R	stereo	99	R	114, VCI; 96, POI; 94, WMI; 94, PSI	N/A	FH	14	English
SG101	M	40	R	stereo	104	B	115, FSIQ	N/A	FIA	24	English
SG102	M	49	R	stereo	86	B	86, FSIQ	N/A	FA, FIA, FBTC	12	English
SG103	F	57	R	stereo	76	B	77, FSIQ	N/A	FA, FIA, FBTC	1.5	English
SG104	F	48	R	stereo	72	B	90, FSIQ	N/A	FIA	30	English

N/A – not applicable, F – female, M – male, L – left, R – right, A – ambidextrous, B – bilateral; FSIQ – Full Scale Intelligence Quotient, VCI – Verbal Comprehension Index, POI – Perceptual Organization Index, WMI – Working Memory Index, PSI – Processing Speed Index; FBTC – focal to bilateral tonic-clonic, FIA – focal impaired awareness, FA – focal aware seizures, FM – focal motor, FH – focal hemiclonic seizure.

Supplementary Table 25. Characteristics of iEEG patients.

10. Anatomical Regions-of-interest (ROIs)

Anatomical ROIs (Destrieux atlas)	
**Short name**	**Long Name**
**Posterior ROI**	
G_and_S_occipital_inf	Inferior occipital gyrus (O3) and sulcus
G_cuneus	Cuneus (O6)
G_occipital_middle	Middle occipital gyrus (O2, lateral occipital gyrus)
G_occipital_sup	Superior occipital gyrus (O1)
G_oc-temp_lat-fusifor	Lateral occipito-temporal gyrus (fusiform gyrus, O4-T4)
G_oc-temp_med-Lingual	Lingual gyrus, ligual part of the medial occipito-temporal gyrus, (O5)
G_oc-temp_med-Parahip	Parahippocampal gyrus, parahippocampal part of the medial occipito-temporal gyrus, (T5)
G_temporal_inf	Inferior temporal gyrus (T3)
Pole_occipital	Occipital pole
Pole_temporal	Temporal pole
S_calcarine	Calcarine sulcus
S_intrapariet_and_P_trans	Intraparietal sulcus (interparietal sulcus) and transverse parietal sulci
S_oc_middle_and_Lunatus	Middle occipital sulcus and lunatus sulcus
S_oc_sup_and_transversal	Superior occipital sulcus and transverse occipital sulcus
S_temporal_sup	Superior temporal sulcus (parallel sulcus)
**PFC ROI**	
G_and_S_cingul-Ant	Anterior part of the cingulate gyrus and sulcus (ACC)
G_and_S_cingul-Mid-Ant	Middle-anterior part of the cingulate gyrus and sulcus (aMCC)
G_and_S_cingul-Mid-Post[a]	Middle-posterior part of the cingulate gyrus and sulcus (pMCC)
G_front_inf-Opercular	Opercular part of the inferior frontal gyrus
G_front_inf-Orbital	Orbital part of the inferior frontal gyrus
G_front_inf-Triangul	Triangular part of the inferior frontal gyrus
G_front_middle	Middle frontal gyrus (F2)
G_front_sup	Superior frontal gyrus (F1)
Lat_Fis-ant-Horizont	Horizontal ramus of the anterior segment of the lateral sulcus (or fissure)
Lat_Fis-ant-Vertical	Vertical ramus of the anterior segment of the lateral sulcus (or fissure)
S_front_inf	Inferior frontal sulcus
S_front_middle	Middle frontal sulcus
S_front_sup	Superior frontal sulcus

[a]Not usually considered part of the PFC, but relevant for GNWT

Supplementary Table 26. Theory defined anatomical regions-of-interest (ROIs) labelled in Destrieux et al. (2010) atlas used for testing theories predictions (unless otherwise specified).

10.1 Process for the definition of the ROIs

At the start of the Cogitate collaboration in March 2018, the literature lacked precise definitions of Regions of Interest (ROIs) for the neural bases of the Global Neuronal Workspace

Theory (GNWT) and Integrated Information Theory (IIT). Terms like 'fronto-parietal network' for GNWT and 'posterior hot zone' for IIT were vague. We noted in an earlier paper[60] that this vagueness allowed for flexible, often biased interpretations of findings.

In 2021, we compiled a list of ROIs from past studies using the Desikan/Killiany atlas[61]. However, during our optimization phase, we found these ROIs too coarse. The adversaries were then asked to provide a more fine-grained ROIs lists. The selected ROIs were aimed at testing the predictions put forward by the theory proponents, rather than representing the full set of brain regions relevant to each theory. Therefore, we selected only the ROIs relevant for the visual experiment performed here. For example, this is the reason the precuneus and posterior cingulate cortex (which are thought to play a role in self, episodic memory, spatial navigation, etc.[62–65] were not included in the IIT ROIs. The list was received in December 2021. These were then refined using the Destrieux atlas for greater accuracy, and finally approved in June 2022

During this time, only initial results from the optimization phase were shared within the consortium. After finalizing the ROIs, we tested the main theories using two-thirds of the data reserved for this purpose. The final ROIs from the adversaries matched our initial list, suggesting no bias in ROI selection, despite initially using broader ROIs.

Supplementary Discussion

11. Cogitate Consortium

Our adversarial collaboration is designed more to challenge theories than to confirm them. Both theories have some predictions confirmed, but these predictions are also consistent with other theories, so the successful predictions cannot serve as evidence for IIT or GNWT specifically. However, the disconfirmed predictions are certainly challenges to both theories (and to others, as discussed above). These challenges can be met by altering the theories or their proposed biological implementation, but such alteration typically comes at some cost to the theoretical framework, because the relevant features of the theory or the implementation were motivated by the framework. In this respect, our adversarial collaboration approach subscribes to the approach advocated by Lakatos[13], a sophisticated version of Popper's falsificationism[14], whereby scientific knowledge advances through a process of conjectures and refutations. When a theory makes an unsuccessful prediction, the challenged theory can survive by refining its details. But if unsuccessful predictions continue, the theory can be deemed a degenerate rather than a progressive research program[15]. This process is expected to be continued by the results of our second experiment (reported in a future manuscript), alongside those of a follow-up adversarial collaboration using a comparable experimental design in animal models (i.e., mice and non-human primates). With time, we hope that substantial evidence will be gathered, allowing the scientific community to form an informed judgment about both theories and possibly others (through the open data). This might be important, as some have proposed a theory-inspired approach to inferring consciousness in non-responsive populations such as unresponsive patients, infants, non-human animals and artificial systems[16–18].

Our study, while comprehensive, is not without its limitations. First, despite our best efforts to minimize the contribution of task relevance by making some stimulus features relevant on some trials and irrelevant in others, we cannot rule out some residual task engagement with respect to category. However, this potential bias is addressed by our deliberate choice to make features like orientation and duration always irrelevant to the task. This approach strengthens the test for the theories we are examining, as any detected effects on these features cannot be attributed to selective attention driven by task requirements, no matter how minimal. Second, although we made our best efforts to capture the richness of experience by investigating multiple dimensions of conscious experience (i.e., category, orientation, identity and duration), we acknowledge that our efforts are still far from measuring consciousness in a way that truly captures its apparent phenomenal richness (e.g., an object's brightness and hue, its precise shape and location, the highly specific viewpoint from which an object is perceived, etc.). Future studies will be needed to address this further. Third, although our study offers superior spatial and temporal resolution across the brain by integrating three distinct brain imaging techniques—fMRI, MEG, and iEEG—it falls short of incorporating single-unit recordings. Such recordings, typically reserved for a small subset of epilepsy patients and limited to certain brain areas like the Medial Temporal Lobe, are impractical for directly testing our theories. Studies in other animal models, including Neuropixels and causal manipulations, are underway as part of a different adversarial collaboration, and are expected to complement our findings. Despite the inherent challenges of using animal studies to probe consciousness (difficulty of measuring consciousness in non-human participants and the limited spatial coverage of Neuropixels probes, and overtraining), we see these two adversarial collaborations as synergistic, providing a stronger test for the theories than either one alone.

12. Integrated Information Theory: Melanie Boly, Christof Koch, Giulio Tononi

The results corroborate IIT's overall claim that posterior cortical areas are sufficient for consciousness, and neither the involvement of PFC nor global broadcasting are necessary. They support preregistered prediction #1, that decoding conscious contents is maximal from posterior regions but often unsuccessful from PFC, and prediction #2, that these regions are sustainedly activated while seeing a stimulus that persists in time. They do not support prediction #3 concerning sustained synchrony, although this negative finding is quite possibly the result of sparse electrode coverage (see 12.1 Further observations). Below we illustrate how these predictions were motivated by IIT.

Posterior regions are often considered mere 'information processors'; their activation, it is claimed, may be necessary but not sufficient for experiencing specific contents. For example, they may show activations during deep sleep or anesthesia and for unreported stimuli under contrastive, near-threshold paradigms[19]. This seems to warrant the need for additional ingredients, such as 'global broadcasting'[19] or 'higher-order monitoring' by PFC[20].

For IIT, however, posterior regions are sufficient for consciousness as long as they satisfy the requirements for maximal integrated information. Why this prediction? Unlike other approaches, IIT infers the essential physical requirements for the substrate of consciousness from the essential properties of experience[21,22]. This leads to the claim that the quality and quantity of an experience are accounted for by the 'cause–effect structure' specified by a substrate with maximal integrated information, called the 'main complex'[21,22]. We conjectured that posterior cortical regions should provide an excellent substrate for the main complex owing to their dense local connections arranged topographically into a hierarchical, divergent–convergent 3D lattice[22], leading to prediction #1. Nevertheless, by IIT, posterior regions can only support consciousness if their physiology ensures high integrated information—which indeed breaks down[23] due to bistability when consciousness is lost in deep sleep and anesthesia[24–26].

Much of PFC, in contrast, seems to be organized not as a grid but as a patchwork of segregated columns[27], unfavorable for high integrated information. Even so, any PFC region organized in a grid-like way with dense interconnections with posterior regions may well be part of the main complex. As previously emphasized[28], *"…we bear no preconceived enmity to the prefrontal cortex. Indeed, searching for the NCC of specific aspects of experience…in certain anterior regions is an important task ahead*." For example, parts of IFG might contribute to, say, an abstract/evaluative/actionable experiential aspect of faces, which could be consistent with some pNCC analysis results. However, IIT predicts that we would still experience faces (sans aspects contributed by PFC regions) if PFC were selectively inactivated.

For IIT, all quality is structure: all properties of an experience are accounted for by properties of the cause–effect structure specified by the main complex. Every conscious content (face, object, letter, blank screen) is thus a (sub)structure of integrated information (irreducible cause-effects and their overlaps[21]); it is neither a message[29] that is encoded and broadcasted globally[19,30,31], nor a distributed activity pattern, nor a neural process. Indeed, IIT's research program aims to account for specific conscious contents—why space feels extended, time feels flowing, and phenomenal objects feel like binding general concepts (invariants) with particular features—all exclusively in terms of their corresponding cause-effect structures[21,32,33]. As highlighted in the Introduction, when we see Mona Lisa, we see that it is a face, with her particular features, at a particular location on the canvas, and we see her for as long as we look at her. This is why we predicted (prediction #2) that the NCC in posterior cortex would last for the duration of the percept, notwithstanding the widespread evidence for neural adaptation and onset/offset neural responses (probably due to transient excitation/inhibition imbalance), and (prediction #3) that synchrony would occur (reflecting causal binding) between units in higher and lower areas, supporting respectively invariant concepts and particular features.

To conclude, moving beyond the contrastive paradigm between seen and unseen stimuli and beginning to account for how experience feels is one key reason why the experiments reported in this adversarial collaboration mark an important development. Another is that they inaugurate a powerful new way of making progress on a problem often considered beyond the reach of science. The group that carried out this endeavor did so in a way that was explicit, open, and truly collaborative—in short, in a way that is paradigmatically scientific.

12.1 Further observations

The present results failed to confirm IIT's prediction about sustained, content-specific synchrony in the gamma range between category-specific cortical regions and V1/V2. By IIT, when we see, say, a face with its contours, features, and location in space, there should be 'causal relations' among all the units contributing those contents. Relations require an overlap between the intrinsic causes and effects of those units[21,32], and those are likely to result in increased synchrony among active units, hence the prediction. Given the lack of evidence for sustained synchrony in the gamma band, the prediction may be wrong with respect to the frequency range. Synchrony may occur instead in lower frequency ranges that may be more sensitive to broader cortical interactions (see e.g. Casimo et al.[34]). Indeed, main results using iEEG (Figure 4b) show increased content-specific synchrony (PPC) between category-specific cortical regions and V1/V2 (but not with PFC) in the 2-25 Hz frequency range between 0 and ~750 ms post-stimulus onset.

The failure to confirm sustained synchrony in the gamma range with iEEG in the present study may also stem from technical limitations. As pointed out in the main text, there were only 12 iEEG electrodes in V1/V2 (against 472 in PFC), with only a minority showing sustained activity for longer trials – which was required to include them in the synchrony analysis. Of note, the analysis of iEEG data did not strictly follow pre-registered analyses methods for selecting ROIs, which required restricting measures of synchrony with anatomical V1/V2 to 'category-selective' channels (showing a stronger response to either faces compared to objects or vice versa) exclusively showing sustained activation compared to baseline in all three pre-specified time windows (0.3-0.5, 0.5-0.8 and 1.3-1.5 sec). Instead, the final analysis included 'category-selective' channels showing an increase in activity, compared to baseline, in any time window. Such a lenient selection of ROIs decreased the relevance of the synchrony analysis to test IIT by including many areas which were less likely to be NCC. It may also partially explain findings of transient rather than sustained synchrony patterns. Results of an ongoing adversarial collaboration replicating the present paradigm using large-scale iEEG recordings along with single-units in monkeys (https://www.templetonworldcharity.org/accelerating-research-consciousness-our-structured-adversarial-collaboration-projects) may help resolve these issues and further test this prediction of IIT.

In the present experiment, MEG ROIs for synchrony analyses were chosen using spatial filters defined on broad-band ERFs, rather than based on local gamma-band activation. Because broad-band ERFs likely comprise signals from both activated and deactivated brain areas (e.g. Vidal et al.[35]; Mukamel et al.[36]), measuring changes in synchrony averaged across such ROIs did not formally test IIT's pre-registered prediction, which only concerns activated areas.

While several findings revealed sustained tracking of duration in iEEG HGP both within V1/V2 and inferior temporal cortex, results of other analyses (especially for orientation RSA and synchrony) failed to identify such a sustained pattern (instead decaying beyond 500 ms). One possible explanation for such discrepancy is that the two latter analyses strongly depend on content-specificity over time. This poses a challenge in the case of V1-V2 units, which contribute low-level stimulus features and were sparsely sampled. Note that micro-saccades appear to be more numerous beyond 500 ms after stimulus onset (Supplementary Figure 7), implying that the perceived location of faces or objects in the visual field may have shifted slightly. If so, IIT predicts that a different set of units in V1/V2 (sensitive to the different retinotopic location) would

contribute to experienced low-level features, and thus to representational similarity and synchronization with category-specific units in higher areas. Again, animal experiments with denser iEEG sampling of V1/V2 may be necessary to resolve this issue.

The finding of a few fMRI-activated voxels in the PFC in the pNCC analysis may have various explanations. One possibility, mentioned in the main text, is that such areas may genuinely contribute some content of consciousness (such as some abstract/evaluative/actionable aspect of faces[11,28,37]). However, the pNCC contrast against baseline is admittedly not very specific (much less than duration-tracking using iEEG). Additionally, none of the activated PFC areas also showed significant fMRI decoding against baseline, further questioning their relevance for consciousness. The weak PFC fMRI activation patterns may also reflect inputs to PFC from posterior cortex or non-specific onset responses (found to be widespread within PFC areas using iEEG, in contrast with absent PFC duration-tracking). Further experiments employing slowly morphing stimuli may shed light on what determines such onset (or offset) responses.

IIT's prediction #1 of less consistent decoding of conscious contents in PFC compared to posterior cortex was verified not only through 1) a failure of decoding orientation in both iEEG and fMRI datasets, but also by 2) a failure of cross-task decoding for letter vs false fonts in PFC using fMRI (Extended Data Figure 1a); 3) a lack of duration tracking for decoding in PFC using iEEG (Extended Data Figure 1d-e); and 4) a lack of cross-task iEEG decoding for faces vs. objects using pseudotrials (Extended Data Figure 1g). In contrast, positive results were consistently found in posterior cortical areas. IIT's prediction of maximum decodability within posterior regions, with no significant additional information about content added by PFC regions, was confirmed using two different multivariate model comparison methods. These results all fit with IIT's prediction that posterior cortical areas are the primary constituents of a complex having maximal integrated information.

Of note, both the sparsity of iEEG electrodes showing duration-tracking (prediction #2) and the widespread deactivation patterns found in PFC (pNCC analysis) strongly suggest that changes in contents of consciousness may be supported by localized activity changes, rather than by a global 'broadcasting' and ignition across the brain. These findings are in line with IIT's prediction that local changes in the state of the substrate of consciousness are sufficient to determine changes in its contents.

13. Global Neuronal Workspace Theory: Stanislas Dehaene

This unprecedented data collection effort brings several new insights relevant to our theory. Most importantly, the results confirm that PFC exhibits a metastable bout of activity ("ignition") for about ~200 ms, in a content-specific manner, even for task-irrelevant stimuli, irrespective of stimulus duration (Figures 2b, 3f, Supplementary Figure 24), and with a concomitant transient increase in long-distance dynamic functional connectivity with face- and object-selective posterior areas (Figure 4a-d). Those findings, unpredicted by IIT but predicted by GNWT, support previous findings that PFC contains a detailed code for conscious visual contents[38–43]. They also counter previous conclusions that were, in our opinion, too hastily drawn

on the basis of insufficient evidence[44]: with suitably sensitive experiments, content-specific PFC regions do show a transient ignition even for irrelevant stimuli. While agreeing with previous results[43,45–48], the convergence of iEEG, MEG and fMRI in the same task alleviates concerns associated with a possible mis-reconstruction of MEG sources. It also resolves a controversy related to the timing of conscious ignition, which was initially thought to be associated with the P300 ERP waveform[19], but can obviously arise earlier (~200 ms post-onset)[45,47]. GNWT would further predict that this latency should vary depending on the strength of both bottom-up accumulating evidence (e.g., contrast[49]) and top-down attention/distraction by other tasks[45,48,50].

While some results do challenge GNWT, they do not seem insurmountable given experimental limitations. First, note that there is a considerable asymmetry in the specificity of the theories' predictions. None of the massive mathematical backbone of IIT, such as the φ measure of awareness, was tested in the present experiment. Instead, what are presented as unique predictions of IIT (posterior visual activation throughout stimulus duration) are just what any physiologist familiar with the bottom-up response properties of those regions would predict, since visual neurons still respond selectively during inattention or general anesthesia[51–53]. Such posterior stimulus-specific, duration-dependent responses are equally predicted by GNWT, but attributed to non-conscious processing.

Unfortunately, here, it is impossible to decide which of the activations reflected conscious versus non-conscious processing, because the experimental design did not contrast conscious versus non-conscious conditions (fortunately, a second experiment by the Cogitate consortium will include such a contrast). The present experiment relied on the seemingly innocuous hypothesis that stimuli were "indubitably consciously experienced" for their entire duration. However, it is well known that perfectly *visible* stimuli, depending on attention orientation, may fail to be *seen* (attentional blink, inattentional blindness)[54,55] or may become conscious at a time decoupled from stimulus presentation (psychological refractory period, retro-cueing)[50,56–58]. Here, it seems likely that participants briefly gained awareness of all the images (since they remembered them later), but then reoriented their conscious thoughts to other topics, without waiting for image offset – and this interpretation perfectly fits the ignition profile that was found in PFC. It would be surprising if participants' consciousness remained tied to each image for its full duration on every trial of this long experiment. It is also unclear whether participants were ever aware of stimulus orientation, which was always irrelevant. A new experiment, using quantified introspection[50], will be needed to assess for how long participants maintained the visual image in consciousness.

For the same reason, the absence of decodable activation at stimulus offset, while challenging, may simply indicate that participants never consciously attended to that event, which was always uninformative and irrelevant. Making stimulus offset more attractive, for instance by turning it into an occlusion event where an object hides behind a screen, could yield different results.

For GNWT, the prefrontal code for a conscious mental object is thought to involve a vector code distributed over millions of neurons which, unlike in posterior regions, are not clustered but spatially intermingled[40,59]. Thus, we are not surprised that PFC responses are hard to decode from the macro- or mesoscopic signals measured by fMRI, MEG, or large intracranial electrodes that pool over tens of thousands of neurons. Therefore, the present positive results, indicating transient PFC ignition and decoding of faces and objects, seem to us more important

than the null ones, especially as there is already much single-neuron evidence that PFC contains even more precise stimulus-specific neural codes [38–41].

Finally, while the theories concern the necessary regions for conscious experience, the present methods are purely correlational and do not evaluate causality. This limitation is not unique to the present work, but applies to any brain-imaging experiment. While applauding the present efforts, we therefore eagerly await the results of other adversarial collaborations using causal manipulations in animal models.

Supplementary Notes

14. Deviations from the preregistration document

The theoretical predictions and analyses presented in this work are described in a preregistration document (https://osf.io/92tbg/), which was updated following the optimization phase of the study and before the analysis of the held-out 2/3 of the data. Here we list the points where this work deviated from the preregistration.

1) In the preregistration document, it is stated that iEEG patients with poor behavioral performance, defined as <70% hits or >30% FAs, were to be excluded (Data quality checks and exclusion of subjects, page 15). This threshold was considered based on a target recruitment of 50 patients. However, due to the coronavirus pandemic and despite our best efforts, only 34 patients were collected at the time of manuscript completion. To weigh the pros and cons of data inclusion and to increase sample size and coverage to better test the theories, it was decided to include in the analysis three iEEG patients whose behavior fell marginally short of the predefined behavioral criteria (i.e., hits < 70%, FA > 30%) to compensate for the lower number of participants.

2) In the preregistration document, the section Exclusion criteria (page 15), mentions the minimum number of trials per condition needed to include participants. For fMRI, this number was set to 20 trials. As individual fMRI trials are not rejected, this criterion was not applied. Instead, unless excluded for other reasons, all participants who completed the full experiment were included. Two participants were excluded because data acquisition was interrupted during a run.

3) In the Data quality checks and exclusion of subjects (page 16) of the preregistration document, it is stated that out of the 120 collected datasets, "overall, 110 fMRI datasets (55 from DCCN and 55 from Yale) passed the quality checks." This is correct, but after the optimization phase, while running the analyses on the held-out data, we found that two datasets collected at Yale had incomplete data (i.e., a run was interrupted before completion). These datasets had to be excluded, bringing the number of included datasets down to 108, which is correctly reported in the Methods document.

4) In the preregistration document, the description of the Representation Dissimilarity Matrices (RDM) computation (page 30) proposes a method in which, to avoid overfitting

to temporally correlated noise fluctuations, trials are split into two separate sets. This method does not use the data maximally, as it halves the number of samples on which to compute the within-class similarity. Instead, the method adopted here does not split the data and computes an RDM by calculating the correlation across all pairs of trials.

5) The description of the fMRI generalized psychophysiological interaction (GPPI) analysis in the preregistration document (page 47) states that this analysis was to be performed independently in the task-relevant and task-irrelevant conditions. These results are presented in the Extended Data Figure 6. To increase statistical power, the results presented in the Figure 4 combined the data of the task-relevant and task-irrelevant conditions.

6) In the same section as the previous point, it was stated that for the GPPI analysis, FDR would be used as the method for correcting for multiple comparisons. Instead, we used the cluster-based permutation test method, as it can profit from the spatial structure of the data and therefore affords higher statistical power.

7) In addition to the preregistered phase synchrony analysis, we also performed a connectivity analysis based on dynamical functional connectivity (DFC). The reason for adding this extra analysis was that we could not find any connectivity through phase-locked synchrony. Since connectivity analyses are very sensitive to noise, especially those that are phase-dependent, we decided to evaluate methods that are more robust. DFC is based on amplitude, making it less susceptible to noise issues.

8) Due to the low number of electrodes in V1/V2 and FFA/LOC, the preregistered criterion of ‘sustained content-specific activation’ was relaxed. Instead, all electrodes located in V1/V2 and those showing content specificity for faces or objects were included in the analysis, regardless of whether they showed sustained activation. It was sufficient if these electrodes responded to the images (V1/V2) and/or were selective to the measured category.

During the review process, we identified other minor deviations from the preregistration document, or internal inconsistencies in the document, which for clarity, we list here:

1) In the preregistration document, in the Design and variables section (page 13) and some other sections, the experimental factor “orientation” is described as having two levels (side view and front view). But as described in the Stimuli section (page 17), half of the stimuli had a frontal orientation, one-fourth were oriented toward the left, and one-fourth toward the right. As left and right-oriented stimuli are experienced as being different, we now more correctly describe the factor “orientation” as having three levels (left, right, and front).

2) During the last amendment to the preregistration, which happened after the optimization phase, the section Decoding performance evaluation (page 47) was edited to correctly describe orientation decoding problems as 3-class problems (left, right, and front views). Unfortunately, other sections of the preregistration documentation were overlooked during the amendment and still present the factor “orientation” as only having two levels (front

view and side view). Note that this is not a deviation from the preregistration but rather an inconsistency within the preregistration document.

3) In the "Subjects, sample size, stopping rule section" (page 14) of the preregistration document, it is stated that in the case of fMRI, data from 122 participants were collected. Two of these participants took part in Experiment 2 but did not take part in Experiment 1. Therefore, the correct number of fMRI datasets for Experiment 1 is 120. This is correctly reported in the Methods document.

4) In the Division of data into phases (page 16) of the preregistration document, it is stated that for the fMRI modality, 36 datasets were included in the optimization phase and 80 in the final testing phase. This is incorrect and inconsistent with the Data quality checks and exclusion of subjects' section of the preregistration document (page 15). The correct numbers of datasets are reported in the Main and Methods documents. These are 17 datasets acquired at the Donders Institute and 18 acquired at Yale for the optimization phase (35 in total), and 38 datasets acquired at the Donders Institute and 35 acquired at Yale for the final testing phase (73 in total). Note that this is not a deviation from the preregistration but rather an inconsistency within the preregistration document.

5) In the preregistration document, in the description of the preprocessing for the decoding analysis for the MEG modality (page 38), there is mention of "twelve GNW ROIs" and "twelve IIT ROIs." The Methods document specifies six ROIs per theory. The reason for the discrepancy is that in the preregistration, each ROI was counted twice (once for each hemisphere).

6) In the preregistration document, in the section Analysis-specific functional preprocessing for the fMRI modality (page 45), it is stated that ROI analyses were going to be performed in each subject's native T1w space. Instead, all analyses were performed after resampling data into standard space.

15. Author Contributions Grid

In an effort to provide greater transparency and assign the appropriate credit to the authors of this paper, Supplementary Figure 55 illustrates the rated CRediT Contribution Matrix. All listed authors provided a self-assessment of their respective contribution for each of the fourteen CRediT categories on a four-point scale. Here we clearly see each person's total contribution across the project (vertically) and the distribution of work for each role (horizontally). After we accumulated all author's self-rankings, all members were given the opportunity to view a draft of the Contribution Matrix and adjust their own ratings relative to their peers, as well as review and comment on other authors' ratings, as a means of normalizing the ratings.

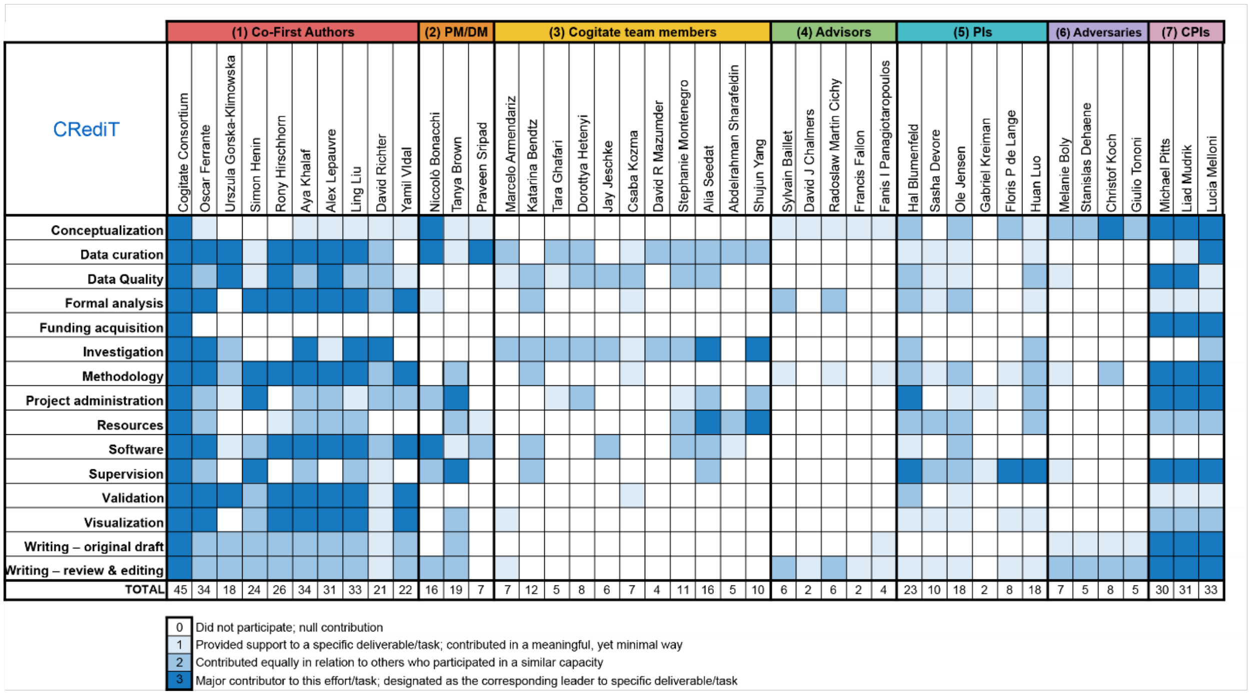

Supplementary Figure 55. Contribution matrix towards the work that went into the production of this paper. CRediT roles are listed vertically in the left column, while each author's name is listed horizontally along the top, in the order in which they appear in the author listing. Above the author names is the seven designated author categories, in accordance with the Cogitate Publication Policy v2; (1) co-first authors; (2) Project/Data Managers; (3) additional Cogitate members; (4) Scientific advisors; (5) Site Principal Investigators (PIs); (6) Adversaries; and (7) Centre PIs. Within each author category, authors are listed in alphabetical order. Each member ranked their own contributions for each of the fourteen CRediT roles according to a four-point scale: 0 – null contribution; 1 – support or minimal contributor; 2 – equal or moderate contributor; and 3 – lead or major contributor.

References

1. Hautus, M. J. Corrections for extreme proportions and their biasing effects on estimated values of d′. *Behav. Res. Methods Instrum. Comput.* **27**, 46–51 (1995).

2. Melloni, L. *et al.* An adversarial collaboration protocol for testing contrasting

predictions of global neuronal workspace and integrated information theory. *PLOS ONE* **18**, e0268577 (2023).

3. Pelli, D. G. The VideoToolbox software for visual psychophysics: transforming numbers into movies. *Spat. Vis.* **10**, 437–442 (1997).
4. O'Reilly, J. X., Woolrich, M. W., Behrens, T. E. J., Smith, S. M. & Johansen-Berg, H. Tools of the trade: psychophysiological interactions and functional connectivity. *Soc. Cogn. Affect. Neurosci.* **7**, 604–609 (2012).
5. Mumford, J. A., Turner, B. O., Ashby, F. G. & Poldrack, R. A. Deconvolving BOLD activation in event-related designs for multivoxel pattern classification analyses. *NeuroImage* **59**, 2636–2643 (2012).
6. Benjamini, Y. & Hochberg, Y. Controlling the False Discovery Rate: A Practical and Powerful Approach to Multiple Testing. *J. R. Stat. Soc. Ser. B Methodol.* **57**, 289–300 (1995).
7. Gerber, E. M., Golan, T., Knight, R. T. & Deouell, L. Y. Cortical representation of persistent visual stimuli. *NeuroImage* **161**, 67–79 (2017).
8. Combrisson, E., Basanisi, R., Cordeiro, V. L., Ince, R. A. A. & Brovelli, A. Frites: A Python package for functional connectivityanalysis and group-level statistics of neurophysiological data. *J. Open Source Softw.* **7**, 3842 (2022).
9. Aru, J., Bachmann, T., Singer, W. & Melloni, L. Distilling the neural correlates of consciousness. *Neurosci. Biobehav. Rev.* **36**, 737–746 (2012).
10. Mashour, G. A., Roelfsema, P., Changeux, J.-P. & Dehaene, S. Conscious Processing and the Global Neuronal Workspace Hypothesis. *Neuron* **105**, 776–798 (2020).

11. Boly, M. *et al.* Are the Neural Correlates of Consciousness in the Front or in the Back of the Cerebral Cortex? Clinical and Neuroimaging Evidence. *J. Neurosci.* **37**, 9603–9613 (2017).

12. Dehaene, S. Towards a cognitive neuroscience of consciousness: basic evidence and a workspace framework. *Cognition* **79**, 1–37 (2001).

13. Lakatos, I. *Proofs and Refutations: The Logic of Mathematical Discovery*. (Cambridge University Press, Cambridge New York Melbourne, 1976).

14. Popper, K. *The Logic of Scientific Discovery*. (Routledge, 1959). doi:10.4324/9780203994627.

15. Melloni, L. On keeping our adversaries close, preventing collateral damage, and changing our minds. Comment on Clark et al. *J. Appl. Res. Mem. Cogn.* **11**, 45–49 (2022).

16. Birch, J. Should Animal Welfare Be Defined in Terms of Consciousness? *Philos. Sci.* **89**, 1114–1123 (2022).

17. Aru, J., Larkum, M. E. & Shine, J. M. The feasibility of artificial consciousness through the lens of neuroscience. *Trends Neurosci.* **46**, 1008–1017 (2023).

18. Mudrik, L., Mylopoulos, M., Negro, N. & Schurger, A. Theories of consciousness and a life worth living. *Curr. Opin. Behav. Sci.* **53**, 101299 (2023).

19. Dehaene, S. & Changeux, J.-P. Experimental and Theoretical Approaches to Conscious Processing. *Neuron* **70**, 200–227 (2011).

20. Dehaene, S., Lau, H. & Kouider, S. What is consciousness and could machines have it? *Science* (2017) doi:10.1126/science.aan8871.

21. Albantakis, L. *et al.* Integrated information theory (IIT) 4.0: Formulating the properties

of phenomenal existence in physical terms. *PLOS Comput. Biol.* **19**, e1011465 (2023).

22. Tononi, G., Boly, M., Massimini, M. & Koch, C. Integrated information theory: from consciousness to its physical substrate. *Nat. Rev. Neurosci.* **17**, 450–461 (2016).
23. Pigorini, A. *et al.* Bistability breaks-off deterministic responses to intracortical stimulation during non-REM sleep. *NeuroImage* **112**, 105–113 (2015).
24. Sarasso, S. *et al.* Consciousness and Complexity during Unresponsiveness Induced by Propofol, Xenon, and Ketamine. *Curr. Biol.* **25**, 3099–3105 (2015).
25. Ferrarelli, F. *et al.* Breakdown in cortical effective connectivity during midazolam-induced loss of consciousness. *Proc. Natl. Acad. Sci.* **107**, 2681–2686 (2010).
26. Massimini, M. *et al.* Breakdown of Cortical Effective Connectivity During Sleep. *Science* **309**, 2228–2232 (2005).
27. Watakabe, A. *et al.* Local and long-distance organization of prefrontal cortex circuits in the marmoset brain. *Neuron* **111**, 2258-2273.e10 (2023).
28. Koch, C., Massimini, M., Boly, M. & Tononi, G. Posterior and anterior cortex — where is the difference that makes the difference? *Nat. Rev. Neurosci.* **17**, 666–666 (2016).
29. Zaeemzadeh, A. & Tononi, G. Shannon information and integrated information: message and meaning. Preprint at https://doi.org/10.48550/arXiv.2412.10626 (2024).
30. Blum, L. & Blum, M. A theory of consciousness from a theoretical computer science perspective: Insights from the Conscious Turing Machine. *Proc. Natl. Acad. Sci.* **119**, e2115934119 (2022).
31. Baars, B. J. *A Cognitive Theory of Consciousness*. (Cambridge University Press, Cambridge, 1989).

32. Haun, A. & Tononi, G. Why Does Space Feel the Way it Does? Towards a Principled Account of Spatial Experience. *Entropy* **21**, 1160 (2019).

33. Comolatti, R., Grasso, M. & Tononi, G. Why does time feel the way it does? Towards a principled account of temporal experience. Preprint at https://doi.org/10.48550/arXiv.2412.13198 (2024).

34. Casimo, K. *et al.* Regional Patterns of Cortical Phase Synchrony in the Resting State. *Brain Connect.* **6**, 470–481 (2016).

35. Vidal, J. R. *et al.* Category-Specific Visual Responses: An Intracranial Study Comparing Gamma, Beta, Alpha, and ERP Response Selectivity. *Front. Hum. Neurosci.* **4**, (2010).

36. Mukamel, R. *et al.* Coupling Between Neuronal Firing, Field Potentials, and fMRI in Human Auditory Cortex. *Science* **309**, 951–954 (2005).

37. Koch, C., Massimini, M., Boly, M. & Tononi, G. Neural correlates of consciousness: progress and problems. *Nat. Rev. Neurosci.* **17**, 307–321 (2016).

38. Bellet, M. E. *et al.* Spontaneously emerging internal models of visual sequences combine abstract and event-specific information in the prefrontal cortex. Preprint at https://doi.org/10.1101/2021.10.04.463064 (2021).

39. Panagiotaropoulos, T. I., Deco, G., Kapoor, V. & Logothetis, N. K. Neuronal Discharges and Gamma Oscillations Explicitly Reflect Visual Consciousness in the Lateral Prefrontal Cortex. *Neuron* **74**, 924–935 (2012).

40. Kapoor, V. *et al.* Decoding internally generated transitions of conscious contents in the prefrontal cortex without subjective reports. *Nat. Commun.* **13**, 1535 (2022).

41. Rainer, G., Asaad, W. F. & Miller, E. K. Selective representation of relevant information

by neurons in the primate prefrontal cortex. *Nature* **393**, 577–579 (1998).

42. Liu, S., Yu, Q., Tse, P. U. & Cavanagh, P. Neural Correlates of the Conscious Perception of Visual Location Lie Outside Visual Cortex. *Curr. Biol.* **29**, 4036-4044.e4 (2019).

43. Hatamimajoumerd, E., Ratan Murty, N. A., Pitts, M. & Cohen, M. A. Decoding perceptual awareness across the brain with a no-report fMRI masking paradigm. *Curr. Biol.* **32**, 4139-4149.e4 (2022).

44. Frässle, S., Sommer, J., Jansen, A., Naber, M. & Einhäuser, W. Binocular Rivalry: Frontal Activity Relates to Introspection and Action But Not to Perception. *J. Neurosci.* **34**, 1738–1747 (2014).

45. Sergent, C., Baillet, S. & Dehaene, S. Timing of the brain events underlying access to consciousness during the attentional blink. *Nat. Neurosci.* **8**, 1391–1400 (2005).

46. Del Cul, A., Baillet, S. & Dehaene, S. Brain Dynamics Underlying the Nonlinear Threshold for Access to Consciousness. *PLoS Biol.* **5**, e260 (2007).

47. Dellert, T. *et al.* Dissociating the Neural Correlates of Consciousness and Task Relevance in Face Perception Using Simultaneous EEG-fMRI. *J. Neurosci.* **41**, 7864–7875 (2021).

48. Marti, S., King, J.-R. & Dehaene, S. Time-Resolved Decoding of Two Processing Chains during Dual-Task Interference. *Neuron* **88**, 1297–1307 (2015).

49. Van Vugt, B. *et al.* The threshold for conscious report: Signal loss and response bias in visual and frontal cortex. *Science* **360**, 537–542 (2018).

50. Marti, S., Sackur, J., Sigman, M. & Dehaene, S. Mapping introspection's blind spot: Reconstruction of dual-task phenomenology using quantified introspection. *Cognition* **115**, 303–313 (2010).

51. Pack, C. C., Berezovskii, V. K. & Born, R. T. Dynamic properties of neurons in cortical area MT in alert and anaesthetized macaque monkeys. *Nature* **414**, 905–908 (2001).

52. Desimone, R., Albright, T., Gross, C. & Bruce, C. Stimulus-selective properties of inferior temporal neurons in the macaque. *J. Neurosci.* **4**, 2051–2062 (1984).

53. Moran, J. & Desimone, R. Selective Attention Gates Visual Processing in the Extrastriate Cortex. *Science* **229**, 782–784 (1985).

54. Mack, A. & Rock, I. *Inattentional Blindness*. (MIT Press, Cambridge, Mass., 2000).

55. Simons, D. J. & Chabris, C. F. Gorillas in Our Midst: Sustained Inattentional Blindness for Dynamic Events. *Perception* **28**, 1059–1074 (1999).

56. Sergent, C. *et al.* Cueing Attention after the Stimulus Is Gone Can Retrospectively Trigger Conscious Perception. *Curr. Biol.* **23**, 150–155 (2013).

57. Thibault, L., Van Den Berg, R., Cavanagh, P. & Sergent, C. Retrospective Attention Gates Discrete Conscious Access to Past Sensory Stimuli. *PLOS ONE* **11**, e0148504 (2016).

58. Sigman, M. & Dehaene, S. Brain Mechanisms of Serial and Parallel Processing during Dual-Task Performance. *J. Neurosci.* **28**, 7585–7598 (2008).

59. Xie, Y. *et al.* Geometry of sequence working memory in macaque prefrontal cortex. *Science* **375**, 632–639 (2022).

60. Yaron, I., Melloni, L., Pitts, M. & Mudrik, L. The ConTraSt database for analysing and comparing empirical studies of consciousness theories. *Nat. Hum. Behav.* **6**, 593–604 (2022).

61. Desikan, R. S. *et al.* An automated labeling system for subdividing the human cerebral cortex on MRI scans into gyral based regions of interest. *NeuroImage* **31**, 968–980

(2006).

62. Epstein, R. A., Patai, E. Z., Julian, J. B. & Spiers, H. J. The cognitive map in humans: spatial navigation and beyond. *Nat. Neurosci.* **20**, 1504–1513 (2017).

63. Jacobs, J. *et al.* Right-lateralized Brain Oscillations in Human Spatial Navigation. *J. Cogn. Neurosci.* **22**, 824–836 (2010).

64. Kumral, E., Bayam, F. E. & Özdemir, H. N. Cognitive and Behavioral Disorders in Patients with Precuneal Infarcts. *Eur. Neurol.* **84**, 157–167 (2021).

65. Sestieri, C., Shulman, G. L. & Corbetta, M. The contribution of the human posterior parietal cortex to episodic memory. *Nat. Rev. Neurosci.* **18**, 183–192 (2017).
