# Supplementary material for: Adversarial testing of global neuronal workspace and integrated information theories of consciousness
Source: Nature. 2025 Apr 30;642(8066):133–42. doi: 10.1038/s41586-025-08888-1 (PMC12137136; doi:10.1038/s41586-025-08888-1)
Supplement: Supplementary file 2 — Reporting Summary [file 41586_2025_8888_MOESM2_ESM.pdf]

nature portfolio

Corresponding author(s): Lucia Melloni

Last updated by author(s): Jan 3, 2025

Reporting Summary

Nature Portfolio wishes to improve the reproducibility of the work that we publish. This form provides structure for consistency and transparency in reporting. For further information on Nature Portfolio policies, see our Editorial Policies and the Editorial Policy Checklist.

Statistics

For all statistical analyses, confirm that the following items are present in the figure legend, table legend, main text, or Methods section.

n/a	Confirmed	
☐	☒	The exact sample size (*n*) for each experimental group/condition, given as a discrete number and unit of measurement
☐	☒	A statement on whether measurements were taken from distinct samples or whether the same sample was measured repeatedly
☐	☒	The statistical test(s) used AND whether they are one- or two-sided<br>*Only common tests should be described solely by name; describe more complex techniques in the Methods section.*
☒	☐	A description of all covariates tested
☐	☒	A description of any assumptions or corrections, such as tests of normality and adjustment for multiple comparisons
☐	☒	A full description of the statistical parameters including central tendency (e.g. means) or other basic estimates (e.g. regression coefficient) AND variation (e.g. standard deviation) or associated estimates of uncertainty (e.g. confidence intervals)
☐	☒	For null hypothesis testing, the test statistic (e.g. *F*, *t*, *r*) with confidence intervals, effect sizes, degrees of freedom and *P* value noted<br>*Give P values as exact values whenever suitable.*
☐	☒	For Bayesian analysis, information on the choice of priors and Markov chain Monte Carlo settings
☒	☐	For hierarchical and complex designs, identification of the appropriate level for tests and full reporting of outcomes
☒	☐	Estimates of effect sizes (e.g. Cohen's *d*, Pearson's *r*), indicating how they were calculated

Our web collection on statistics for biologists contains articles on many of the points above.

Software and code

Policy information about availability of computer code

Data collection	iEEG - New York University (NYU), Harvard University (Harvard), University of Wisconsin (WI)<br>Natus Quantum and Neuralynx amplifiers, Matlab versions: Harvard: R2020b; NYU: R2020a, WU: 2021a, Psychtoolbox v3, Eyetracking: Harvard and WU: Eyelink 1000+, NYU: Tobii4c<br><br>MEG- Birmingham University (BU)<br>FaceGen Modeler 3.1, MAXON CINEMA 4D Studio (RC - R20) 20.059, Matlab R2019B, Psychtoolbox 3, MEGIN DALQ 6.0, Eyelink Host PC Software 5.15<br><br>MEG - Peking University (PKU)<br>Matlab R2018B with Psychtoolbox 3, Eyelink Host PC Software 5.15, MEGIN DALQ 6.0<br><br>fMRI - Donders Institute, Centre for Cognitive Neuroimaging (DCCN) and Yale University (Yale)<br>Matlab R2019b, Psychtoolbox v.3.<br><br>Pilot (MPIEA)<br>Psychtoolbox 3 (Brainard, 1997; Pelli, 1997) on Matlab 2017a; Windows 10<br><br>*All experimental paradigm code, used for data collection, can be found at: https://doi.org/10.5281/zenodo.14362838
Data analysis	iEEG<br>Python (v3.9) using open-source packages, MNE (0.24), NumPy (l.23.5), SciPy (l.9.3), Frites (0.4.3), nilearn (0.9.2), ibabel (4.0.2), JZS Bayes

factor (Rouder, et al., 2009)

MEG (BU)
Python (3.9.7), MNE-python (0.24.0), Freesurfer (6.0.1), MNE-BIDS (0.8), Frites (0.4.3), FLUX (1.0)
Algorithms:
Signal-Space Separation (SSS), FastICA, dynamic statistical parametric mapping (dSPM), minimum-norm estimates (MNE), pairwise phase consistency (PPC) - All the algorithms above were performed as implemented in MNE-Python (see versions above).
MEG (PKU)
Python (3.9.7), MNE-python(0.24.0), Freesurfer (6.0.1), Spyder (5.3.3), MNE-bids (0.8), scikit-learn.
When conducting Bayesian analyses, we used the bayes_ttest function in the code "bayes_factor_fun.py" found in our shared github repository (see below).

fMRI (DCCN & Yale)
BIDScoin v3.6.3, BIDS-Validator, MRIQC 0.16.1, fMRIPrep 20.2.3, Nipype 1.6.1, FSL 6.0.2, SPM8, SPM12, Freesurfer 6.0.1, NiBetaSeries 0.6.0, Pingouin 0.5.1, NumPy 1.19.2, Pandas 1.1.3, NiBabel 3.2.2, SciPy 1.8.0, Matplotlib 3.3.2, Scikit-learn 0.23.2
Algorithms: JZS Bayes Factor (Rouder et al. 2009), Support Vector Machine classifier, and generalized Psycho-Physiological Interaction

Behavioural analysis
R 4.3.1, ordinal 2023.12.4, tidyR 1.3.0, dplyr 1.1.4, lmerTest 3.1.3, bayestestR 0.14.0, emmeans 1.10.4, Python 3.9, pandas 1.5.2, numpy 1.21.2, matplotlib 3.6.2, seaborn 0.12.1, scipy 1.7.1

Eye tracking analysis
R 4.3.1, ordinal 2023.12.4, tidyR 1.3.0, dplyr 1.1.4, lmerTest 3.1.3, bayestestR 0.14.0, emmeans 1.10.4, Python 3.9, numpy 1.21.2, pandas 1.5.2, scipy 1.7.1, pycircstat 0.0.2, astropy 4.3.post1, seaborn 0.12.1, matplotlib 3.6.2, statsmodels 0.14.0, matlab.engine 9.11.19 (must use this version), saccade analysis features are based on Engbert & Mergenthaler, 2006, but the vfac parameter is based on Engbert & Kliegl, 2003.

Pilot (MPIEA)
MATLAB 2019

**All analysis code (for all modalities) used for data analysis, can be found at: https://doi.org/10.5281/zenodo.13891328

For manuscripts utilizing custom algorithms or software that are central to the research but not yet described in published literature, software must be made available to editors and reviewers. We strongly encourage code deposition in a community repository (e.g. GitHub). See the Nature Portfolio guidelines for submitting code & software for further information.

Data

Policy information about availability of data

All manuscripts must include a data availability statement. This statement should provide the following information, where applicable:

- Accession codes, unique identifiers, or web links for publicly available datasets
- A description of any restrictions on data availability
- For clinical datasets or third party data, please ensure that the statement adheres to our policy

The full study protocol is available in the preregistration on the OSF webpage (https://osf.io/92tbg/), including a detailed description of the experimental design, the theories' predictions and agreed-upon interpretations of the results, as well as iEEG, MEG, and fMRI data acquisition details, preprocessing pipelines, and data analysis procedures. Deviations from the preregistration are documented throughout the manuscript and summarized in Section 14 of the Supplementary Materials.
All data generated in this study are available under a CC BY 4.0 license. The M-EEG, fMRI, and iEEG datasets are distributed through two methods: as downloadable data bundles and via an XNAT instance, which enables search functionality and single-participant downloads. Data bundles can be accessed at https://www.arc-cogitate.com/data-bundles in raw format (M-EEG raw, fMRI raw, iEEG raw) and BIDS format (M-EEG BIDS, fMRI BIDS, iEEG BIDS). Alternatively, the datasets are accessible via the Cogitate XNAT instance at https://cogitate-data.ae.mpg.de. All distribution formats include robust metadata, and detailed documentation of experimental procedures and dataset structure is available at https://cogitate-consortium.github.io/cogitate-data/.

Research involving human participants, their data, or biological material

Policy information about studies with human participants or human data. See also policy information about sex, gender (identity/presentation), and sexual orientation and race, ethnicity and racism.

Reporting on sex and gender	Findings do not apply to only one sex or gender; gender was not considered in the study design and was determined based on self-reporting by participants/patients
Reporting on race, ethnicity, or other socially relevant groupings	No socially relevant categorization variables were collected during the study
Population characteristics	iEEG participants (mean age 30.88±13.94 years, 18 females, 26 right-handed), and all had a clinical diagnosis of epilepsy<br><br>MEG participants: (mean age 22.79±3.59 years, 54 females, all right-handed), 32 of those datasets were included in the optimization phase (mean age 22.50±3.43 years, 19 females, all right-handed), and 65 in the replication sample (Age = 22.93 ±3.66, 35 of them females, all right-handed).<br><br>fMRI participants (mean age 23.31±3.45 years, 72 female, 107 right handed), 35 of those datasets were included in the

optimization sample (mean age 23.25±3.64 years, 21 females, 34 right handed), and 73 in the replication sample (mean age = 23.29±3.37, 49 females, 71 right-handed).

Pilot (MPI) Thirty-nine participants (26 females, aged between 18 and 59, mean=32.6, std=l2.82, all right-handed) took part in the study. All participants had normal or corrected-to-normal vision. They were recruited from the participant pool of the MPI and received monetary compensation for their participation

Recruitment

iEEG (NYU, Harvard, WI)
Participants for iEEG studies consisted of clinical patients admitted to the Epilepsy Monitoring Unit for the surgical management of epilepsy. Participants were approached to participate in research, and if agreeable, they were consented based on each site's IRB protocol for Research with Human Participants. Referral biases may exist, as not all epilepsy patients are referred for surgery, and those who are tend to share specific characteristics—such as disease severity, epilepsy risk factors, cognitive symptoms, comorbid conditions, and seizure onset zone. Since refractory epilepsy is often associated with cognitive dysfunction and neuropsychological disorders, this referral pattern is an important consideration. Patient enrollment is determined by the clinical teams at each site, not the iEEG research group, making this study akin to a cohort design. Our team only excludes patients who lack cognitive capacity to consent or are too young for participation.

MEG
Campus flyers advertisements and targeted mailing lists (BU); campus advertisements (PKU). Most participants were highly educated, middle/high-class university students for both sites.

fMRI (DCCN & Yale)
Both sites recruited neurotypical adult participants via flyers, online community listservs, and social media. In addition to this, the DCCN recruited participants from an existing pool (SONA subject management tool). There was no selection-bias or any other biased since we targeted enrollment of a representative sample across the different gender, racial, and ethnic groups. Due to safety requirements, anyone with a contraindication for MRI, would not be eligible to participate (e.g. metal or electronic implants, pregnant individuals or persons that experience claustrophobia).

Unlike most neuroscience studies that collect data from a single site and/ore single modality, our study minimizes site-specific biases by integrating data from seven sites across three continents. This diverse, multi-site approach enhances the generalizability and robustness of our findings, reducing the limitations associated with localized participant pools and single-laboratory methodologies.

Pilot (MPIEA)
All participants were recruited using an Max Planck Institute for Empirical Aesthetics (MPIEA) internal recruitment tool (MORLA).

Ethics oversight

Across our 7 data collection sites, ethics approvals metadata is as follows :
1. Responsible institute
2. Protocol number
3. Approving committee

1. Centre for Human Brain Research, University of Birmingham
2. ERN_18-0226AP20
3. Science, Technology, Engineering and Mathematics Ethical Review Committee

1. School of Psychological and Cognitive Sciences, Peking University
2. 2020-05-07e
3. Committee for Protecting Human and Animal Subjects

1. Donders Institute (Centre for Cognitive Neuroimaging) - DCCN
2. File number : 2014-288NL number NL45659.091.14
3. Commissie Mensgebonden Onderzoek Regio Arnhem-Nijmegen

1. Yale School of Medicine
2. 2000027591
3. Human Research Protection Program Institutional Review Board

1. New York University Langone Health
2. i14-02101_CR6
3. Office of Science and Research Institutional Review Board

1. Children's Hospital Corporation d/b/a Boston Children's Hospital
2. 04-05-065R
3. Boston Children's Hospital Institutional Review Board (IRB)

1. University of Wisconsin-Madison
2. ID : 2017-1299
3. IRB UW-Madison

1. Max Planck Institute for Empirical Aesthetics (MPIEA)
2. Nr. 2017 12
3. Ethics Council of the Max Planck Society

Note that full information on the approval of the study protocol must also be provided in the manuscript.

Field-specific reporting

Please select the one below that is the best fit for your research. If you are not sure, read the appropriate sections before making your selection.

☒ Life sciences ☐ Behavioural & social sciences ☐ Ecological, evolutionary & environmental sciences

For a reference copy of the document with all sections, see nature.com/documents/nr-reporting-summary-flat.pdf

Life sciences study design

All studies must disclose on these points even when the disclosure is negative.

Sample size

We collected data in 3 different neuroimaging modalities (iEEG, MEG, fMRI), in addition to behavioural and eye tracking data for all datasets, across 7 sites. Sample sizes for fMRI and MEG were determined as being 2.5 times larger than common sample sizes in the literature for that methodology (Simonshon, 2015; 50 participants per site for fMRI and for MEG). Since we used a within-subject design, this sample size gives us >90% power to detect differences of medium effect size (Cohen's d=>0.5). For iEEG, data collection is variable, as it is based on patient availability.

iEEG (NYU, Harvard, WI): N= 34 (2 excluded)= 32
MEG (BU, PKU) :N= 102 (5 excluded)= 97
fMRI (DCCN, Yale): N=120 (12 excluded)= 108

Total collected; N = 256
Total datasets included in analyses; N=237

____

Pilot Study (reported in Supplementary Information): N= 39

Data exclusions

Data from all modalities were checked at three levels by a Data Monitoring Team (DMT). The first level checks tested whether the datasets contained all expected files keeping their naming conventions, and that all personal information had been removed. The second level checks tested participant's performance with respect to behavior; participants were excluded if their hit rate was lower than 80% or false alarms (FAs) higher than 20% for MEG and fMRI, and for iEEG, a more relaxed criteria of 70% Hits and 30% FAs was used. The third level checks assessed the quality of the neural data. For iEEG, channel rejection was performed independently by both the DMT and iEEG teams, and compared to make sure there were no discrepancies. We then verified that the electrode reconstruction performed by the iEEG team matched the alignment of contacts in participants' MRI. Finally, we checked for massive disturbances in the spectra.

For M-EEG, the first stage of the third-level checks focused on system-related and external noise generators. It was tested using the signal spectra in the empty room recording, the resting state session, and the experiment itself for all sensors. Any sensor and/or specific frequency revealing extensive noise using visual inspection, was flagged to document potential problems. [Ultimately, this did not lead to any exclusions.] Next, all experimental data blocks were visually inspected for abnormalities in spectra (peaks not explainable by physiology), and in ICA components, and checked for extremely noisy (based on the score of differences between the original and Maxwell-filtered data> 7) and flat sensors. The latter step was performed in collaboration between the DMT and members of BU and PKU to check whether any potential changes in preprocessing for particular participants were needed. Finally, we tested if all experimental cells (i.e., task-relevant non-targets and task-irrelevant stimuli for each one of the four categories) had enough trials (N=30).

For fMRI, we combined visual inspection of structural and functional images with automatic criteria for motion-related artifacts. Third level checks of data quality were done using both MRIQC (Esteban et al., 2017) and fMRIprep (Esteban et al., 2020), separately for optimization and replication datasets. The output from MRIQC and fMRIprep was visually inspected. Datasets with clear artifacts and other indicators of low data quality (incorrect reconstructions, and substantial signal dropout or distortion) were marked by a trained observer, and if the detected problems were judged severe enough to warrant potential exclusion, data were additionally inspected together by the DMT and collaborators at DCCN and Yale. In practice, we rejected participants where a significant part of the cortex, roughly > 5%, was not covered by the brain mask (tissue was not segmented). Next, datasets were checked for extensive motion, using MRIQC image quality metrics. Specifically, the percentage of fMRI volumes that exceeded a threshold of 0.2mm framewise displacement (FD) and DVARS (Power et al., 2012) measure were calculated per run and averaged per session. Finally, each MRI session whose percentage framewise displacement or DVARS deviated by more than 2 standard deviations above the group mean were marked for rejection.

____

iEEG
Two (N=2) patients were excluded due to incomplete datasets. Three patients whose behavior fell short of the predefined behavioral criteria (i.e. hits< 70%, FA> 30%), were nonetheless included in the analysis: one kept the response button pressed for most of the time during experiment, the other's low performance was driven by one of the categories only (which the patient reported having difficulty to detect), and the third's performance was very close to the threshold (65%) and had very low FA rate (2%).

MEG
Five (N=5) participants were excluded from the MEG dataset: two due to failure to meet predefined behavioral criteria (i.e., hits< 80%, and/or FA > 20%), two due to excessive noise from sensors, and one due to incorrect sensor reconstruction

fMRI
Twelve (N=12) participants were excluded from the fMRI dataset: seven due to motion artifacts, two due to insufficient coverage, and two due to incomplete data

Eye movement analysis

	Eleven (N=ll) participants/patients were excluded from the eye movement analysis (3 iEEG patients due to no eye tracking data available; 8 for insufficient quality (iEEG, N=4; MEG, N=2; fMRI, N=2)
Replication	All findings from MEG and fMRI were replicated on an independent sample and are reported in the supplementary materials. Due to the limited number of iEEG datasets, replication was not conducted on those data.<br>An initial optimization phase was used on 1/3 of the MEG (N=32) and fMRI (N=35) data. Following optimization, pipelines were preregistered and applied tot he novel datasets containing twice as much data (MEG, N=65 and fMRI, N=73).
Randomization	We used a within-participant design that does not require randomization.
Blinding	We used a within-participant design that does not require blinding.

Reporting for specific materials, systems and methods

We require information from authors about some types of materials, experimental systems and methods used in many studies. Here, indicate whether each material, system or method listed is relevant to your study. If you are not sure if a list item applies to your research, read the appropriate section before selecting a response.

Materials & experimental systems

n/a	Involved in the study
☒	☐ Antibodies
☒	☐ Eukaryotic cell lines
☒	☐ Palaeontology and archaeology
☒	☐ Animals and other organisms
☒	☐ Clinical data
☒	☐ Dual use research of concern
☒	☐ Plants

Methods

n/a	Involved in the study
☒	☐ ChIP-seq
☒	☐ Flow cytometry
☐	☒ MRI-based neuroimaging

Plants

Seed stocks	N/A
Novel plant genotypes	N/A
Authentication	N/A

Magnetic resonance imaging

Experimental design

Design type	task, event related
Design specifications	fMRI Sites: DCCN & Yale<br>Stimuli were presented for one of three durations (0.5 s, 1.0 s or 1.5 s), followed by a blank period of a variable duration to complete an overall trial length fixed at 2.0 s. Random jitter was added at the end of each trial (mean inter-trial interval of 3 s, jittered 2.5-10 s, with truncated exponential distribution), with each trial lasting approximately 5.5 s. There were 8 runs containing 4 blocks each with 17-19 trials per block, 16 non-targets (4 per category) and 1-3 targets, for a total of 576 trials. Rest breaks between runs (at discretion) and blocks (12 seconds) were included.<br><br>*all other sites collected structural MRIs only; no design specifications to report
Behavioral performance measures	fMRI sites: DCCN & Yale<br>Log-linear corrected d'prime, false alarms (FA) and reaction times (RT) were computed per category and stimulus foration, separately (FAs were also calculated per task relevance, without duration), and per modality (iEEG, MEG, 'MRI). These measures were compared with Linear/Logistic mixed models, where appropriate. For the former, we ·eport ANOVA omnibus F tests, and for the latter, omnibus x' test from an analysis of deviance. We approximated fogrees of freedom using the Satterthwaite method. Pairwise t-tests following significant interactions were Bonferroni :orrected. To estimate Bayesian Information Criterion (BIC) differences between the original and null logistic models, we used the p-values and sample size (Wagenmakers: https://psyarxiv.com/egydq; p_to_bf package in R).

*all other sites collected structural MRIs only; no behavioral performance measures to report

Acquisition

Imaging type(s)

anatomical T1 scan (MRI) - NYU, Harvard, WI, BU, PKU, DCCN, Yale
functional MRI (fMRI) - DCCN and Yale

Field strength

3T

Sequence & imaging parameters

MEG - BU:
(Tl); 32-channel head coil (TR/TE= 2000/2.03; Tl= 880ms; Flip angle=S degrees; FOV=256 x 256 x 208; slices= 208; 1mm isotropic voxels)

MEG-PKU:
(Tl); 64-channel head coil (TR/TE= 2530/2.98ms; Tl = 1100 ms; 7° flip angle; FOV = 256x256x208 mm; 198 slices;l mm isotropic voxels, GRAPPA)

fMRI (DCCN & Yale):
Tl; 32-channel head coil, anatomical Tlw MPRAGE images (GRAPPA acceleration factor= 2, TR/TE= 2300/3.03 ms, 8° flip angle, 192 slices, 1 mm isotropic voxels)T2; whole-brain T2*-weighted multiband-4 sequence (TR/TE= 1500/39.6 ms, 75° flip angle, FOV = 210 mm, 68 slices, voxel size 2 mm isotropic, A/P phase encoding direction, BW = 2090 Hz/Px) EPI sequence; CMRR MB-4, TR/TE= 1500/39.6 ms, 68 slices, voxel size 2 mm isotropic, 75° flip angle, A/P phase encoding direction, FOV = 210 mm, BW = 2090 Hz/Px

Area of acquisition

whole-brain scan

Diffusion MRI ☐ Used ☒ Not used

Preprocessing

Preprocessing software

MEG Sites (BU & PKU):
Freesurfer 6.0.1

fMRI Sites (DCCN & Yale):
Source DICOM data were converted to BIDS using BIDScoin v3.6.3.
(f)MRI data was preprocessed using fMRIPrep 20.2.3, based on Nipype 1.6.1. In addition, analysis specific preprocessing were performed using FSL 6.0.2 and custom Python scripts using the following packages: NumPy 1.19.2, Pandas 1.1.3, NiBabel 3.2.2, SciPy 1.8.0, Matplotlib 3.3.2 and Scikit-learn 0.23.2.
fMRIPrep anatomical data preprocessing
The Tl-weighted (Tlw) image was corrected for intensity non-uniformity (INU) with N4BiasFieldCorrection, distributed with ANTs 2.3.3 [RRID:SCR_004757], and used as Tlw-reference throughout the workflow. The Tlw-reference was then skull-stripped with a Nipype implementation of the antsBrainExtraction.sh workflow (from ANTs), using OASIS30ANTs as target template.

Brain tissue segmentation of cerebrospinal fluid (CSF), white-matter (WM) and gray-matter (GM) was performed on the brain-extracted Tlw using fast [FSL 5.0.9, RRID:SCR_002823].
Brain surfaces were reconstructed using recon-all [FreeSurfer 6.0.1, RRID:SCR_001847], and the brain mask estimated previously was refined with a custom variation of the method to reconcile ANTs-derived and FreeSurfer-derived segmentations of the cortical gray-matter of Mindboggle [RRID:SCR_002438].

Analysis-specific functional preprocessing
Additional, analysis-specific, fMRI data preprocessing was performed using FSL 6.0.2 (FMRIB Software Library; Oxford, UK; Smith et al., 2004], Statistical Parametric Mapping (SPM 12) software (Penny et al., 2007), and custom Python scripts. Functional data for univariate data analyses will be spatially smoothed (Gaussian kernel with full-width at half-maximum of 5 mm), grand mean scaled, and temporal high-pass filtered (128 s). No spatial smoothing was applied for multivariate analyses.

Normalization

BU & PKU:
All steps included in Freesurfer reconall

DCCN & Yale:
Volume-based spatial normalization to one standard space (MNl152NLin2009cAsym) was performed through nonlinear registration with antsRegistration (ANTs 2.3.3), using brain-extracted versions of both Tlw reference and the Tlw template.

Normalization template

BU & PKU:
Freesurfer "fsaverage"

DCCN & Yale:
ICBM 152 Nonlinear Asymmetrical template version 2009c (Fonov et al., (2009); RRID:SCR_008796; TemplateFlow ID: MNl152NLin2009cAsym).

Noise and artifact removal

BU & PKU:
-all steps included in Freesurer recon all

DCCN & Yale:
MRI data quality control was performed using MRIQC 0.16.1.
fMRIPrep functional data preprocessing
Head-motion parameters with respect to the BOLD reference (transformation matrices, and six corresponding rotation and translation parameters) were estimated before any spatiotemporal filtering using mcflirt [FSL 5.0.9].

A reference volume and its skull-stripped version were generated using a custom methodology of fMRIPrep. Several confounding time-series were calculated based on the preprocessed BOLD: framewise displacement (FD), DVARS and three region-wise global signals.
FD was computed using two formulations following Power (absolute sum of relative motions, Power et al., 2014) and Jenkinson (relative root mean square displacement between affines, Jenkinson et al., 2002).
FD and DVARS were calculated for each functional run, both using their implementations in Nipype (following the definitions by Power et al., 2014). The three global signals were extracted within the CSF, the WM, and the whole-brain masks.
Additionally, a set of physiological regressors were extracted to allow for component-based noise correction (CompCor, Behzadi et al., 2007).
Principal components were estimated after high-pass filtering the preprocessed BOLD time-series (using a discrete cosine filter with 128s cut-off) for the two CompCor variants: temporal (tCompCor) and anatomical (aCompCor). tCompCor components were then calculated from the top 2% variable voxels within the brain mask. For aCompCor, three probabilistic masks (CSF, WM and combined CSF+WM) were generated in anatomical space.
The implementation differs from that of Behzadi et al. in that instead of eroding the masks by 2 pixels on BOLD space, the aCompCor masks are subtracted a mask of pixels that likely contain a volume fraction of GM. This mask is obtained by dilating a GM mask extracted from the FreeSurfer's aseg segmentation, and it ensures components are not extracted from voxels containing a minimal fraction of GM. Finally, these masks are resampled into BOLD space and binarized by thresholding at 0.99 (as in the original implementation).
Components are also calculated separately within the WM and CSF masks. For each CompCor decomposition, the k components with the largest singular values are retained, such that the retained components' time series are sufficient to explain 50 percent of variance across the nuisance mask (CSF, WM, combined, or temporal). The remaining components are dropped from consideration. The head-motion estimates calculated in the correction step were also placed within the corresponding confounds file. The confound time series derived from head motion estimates and global signals were expanded with the inclusion of temporal derivatives and quadratic terms for each (Satterthwaite et al., 2013).
Frames that exceeded a threshold of 0.5 mm FD or 1.5 standardised DVARS were annotated as motion outliers. All resamplings were performed with a single interpolation step by composing all the pertinent transformations (i.e. head-motion transform matrices, susceptibility distortion correction when available, and co-registrations to anatomical and output spaces). Gridded (volumetric) resamplings were performed using antsApplyTransforms (ANTs), configured with Lanczos interpolation to minimize the smoothing effects of other kernels (Lanczos, 1964). Non-gridded (surface) resamplings were performed using mri_vol2surf (FreeSurfer).

Volume censoring

DCCN & Yale: The first three volumes of each run were discarded to allow for signal stabilization.

Statistical modeling & inference

Model type and settings

Mass univariate: Averaged across runs per participant using FSL's fixed effects analysis and subsequently averaged across participants using FSL's FLAME mixed effect analysis.

Univariate Connectivity Analyses: gPPI analyses were performed on the participant level and contrast maps were averaged across participants.

Multivariate decoding Analyses: Single trial estimates were obtained per each participant and decoding was performed on the participant level using these estimates as features. Decoding accuracies was averaged across participants.

Effect(s) tested

Conjunction analyses:
Areas sensitive to task goal:
targets> bsl & task relevant = bsl & task irrelevant = bsl

Areas sensitive to task-relevance:
targets> bsl & task relevant " bsl & task irrelevant = bsl

Putative NCCs:
(task relevant> bsl & task irrelevant> bsl) V (task relevant< bsl & task irrelevant< bsl)

Decoding analyses:
Areas with decoding accuracy higher than the chance level

Connectivity analyses:
Areas showing significant task-related connectivity

Specify type of analysis: ☐ Whole brain ☐ ROI-based ☒ Both

Anatomical location(s) Freesurfer automatic parselation using the Destrieux 2010 and Wang 2015 atlases

Statistic type for inference

(See Eklund et al. 2016)

Univariate analyses: Gaussian random-field cluster thresholding was used to correct for multiple comparisons, using the default settings of FSL, with a cluster formation threshold of one sided $p < 0.001$ ($z \geq 3.1$,) and a cluster significance threshold of $p < 0.05$. Also JZS Bayes factor analyses.
Univariate task-related connectivity analyses: group level gPPI analyses were performed using cluster-based permutation testing ($p < 0.05$).
Multivariate analyses: Group level searchlight decoding analysis was performed using cluster-based permutation testing ($p < 0.05$) while group level ROI decoding was performed using one sample permutation test.

Correction	Multivariate analyses: We performed FDR correction ($p < 0.05$) across ROIs for ROI decoding.

Models & analysis

n/a	Involved in the study	
☐	☒	Functional and/or effective connectivity
☒	☐	Graph analysis
☐	☒	Multivariate modeling or predictive analysis

Functional and/or effective connectivity	functional connectivity
Multivariate modeling and predictive analysis	Single trial estimates were obtained per participant and used as features for a support vector machines classifier. No feature selection was employed. Performance was evaluated using leave-one-run-one and through training on one condition and testing on the other condition.
